# Epoxide Alcoholysis over M‑BEA Zeolites: Effects of Alcohol Chain Length on Rates and Regioselectivities

**DOI:** 10.1021/acscatal.5c04379

**Published:** 2025-10-02

**Authors:** Huston Locht, David S. Potts, Zahra Rangoonwala, David W. Flaherty

**Affiliations:** 1 School of Chemical and Biomolecular Engineering 1372 Georgia Institute of Technology, Atlanta, Georgia 30332, United States; 2 Department of Chemical and Biomolecular Engineering University of Illinois Urbana−Champaign, Urbana, Illinois 61801, United States

**Keywords:** nucleophilicity, ring opening, acidic zeolites, regioselectivity, solvation, hydrogen bonding

## Abstract

The structures of nucleophilic reactants affect their coordination behavior among solvent molecules and kinetics of reactions with surface intermediates within the confines of fluid-filled pores of zeolites and other microporous materials. Consequently, rates and regioselectivities of diverse chemistries may depend sensitively on nucleophile identity in manners not observed for classic fluid phase reactions. Here, we examine the impact of varying the primary alcohol (ROH) chain length on the kinetics of 1,2-epoxybutane (C_4_H_8_O) ring-opening within Brønsted (Al-BEA) and Lewis acid (Zr-BEA) zeolites. Turnover rates increase by factors of ∼6 (Al-BEA) and 4-fold (Zr-BEA) between methanol and 1-hexanol, yet the reaction mechanisms remain comparable. Despite modest rate differences, apparent activation enthalpies calculated from rates and activities of solvated reactants decrease linearly by 12 (Al-BEA) to 33 kJ mol^–1^ (Zr-BEA) with increased proton affinity, which suggests bond formation energies for the nucleophile strongly influence rate increases. The molecular interpretation of these trends demonstrates, however, that the solvation of ring-opening transition states by zeolite pore structures and solvent molecules also governs rates. The impact of local solvating interactions appears most directly as changes in regioselectivities, which tend to enhance terminal alcohol formation with increasing ROH chain length. Regioselectivities largely do not vary with differences in fluid composition for a given ROH. The addition of H_2_O increases the number of hydrogen bonds among reactive species, and trends in regioselectivities imply that the decreased hydrogen bonding ability of longer chain ROH, and not the nucleophile strength or steric bulk, determines the regioselectivities of the resulting products. This work provides direct experimental evidence that nucleophilicity and hydrogen bonding influence reaction barriers and regioselectivities in zeolite-catalyzed epoxide ring-opening, offering pathways to better control reaction kinetics.

## Graphical abstract

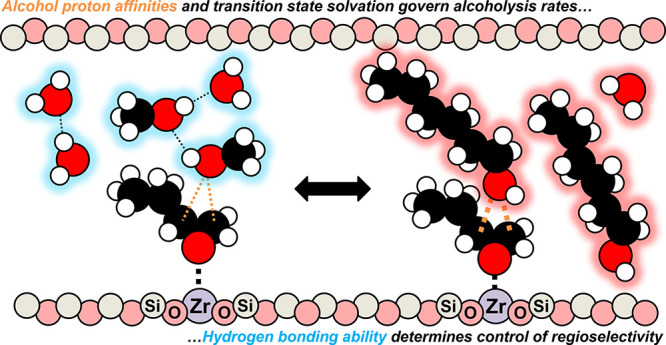

## 1 Introduction

Epoxide ring-opening with alcohol (ROH) nucleophiles yields products with unique applications in the pharmaceutical, solvent, and polymer industries.^1–3^ Nucleophilic attack can occur at either carbon-atom of the oxirane ring to produce distinct regioisomers as products (Scheme 1). The chemical properties and commercial values of the terminal ethers and terminal alcohols that form by these steps differ significantly, consequently, controlling the reaction regioselectivities of epoxide ring-opening reactions remains crucial. Regioselectivities depend upon the local environment that surrounds active sites within catalysts such as homogeneous complexes,^4–6^ metal salts,^7–10^ ion-exchange resins,^11–13^ metal organic frameworks,^14–16^ and zeolites.^17–23^ Among these materials, zeolites provide advantages including facile separation from products, high rates at modest temperatures, and stable structures with diverse topologies among commercially available materials.^24^ The composition of the liquid phase remains important because the nature of the interactions among solvating molecules, reactive intermediates, and the solid catalyst can impact rates and regioselectivities.

**Scheme 1:**
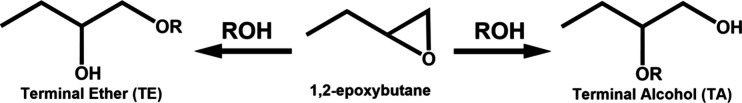
Primary Reaction Products of 1,2-Epoxybutane (C_4_H_8_O) Ring-Opening with Alcohols (ROH)

The structure of liquid-phase reactants alters the complex interactions among solvent molecules, reactive species, and catalytic sites within the micropores of zeolites and thereby modifies the thermodynamics of adsorption and the kinetics of reactions in these environments. Specifically, alkyl chain length affects alkane cracking,^25,26^ alkene epoxidation,^27–29^ and alcohol dehydration^30,31^ reactions through combinations of specific (e.g., hydrogen bonding) and nonspecific (e.g., dispersive) interactions. These interactions introduce nonidealities (excess contributions, *G* ^ε^) that alter the stability of reactive species.^32^ In the absence of solvent, increasing alkane chain length leads to stabilizing enthalpic interactions with the zeolite pore and concomitant entropy losses upon adsorption.^25^ The authors directly attribute entropy losses upon adsorption to large entropic gains when forming alkane cracking transition states, which yield increased activation entropies and rates with increased alkane carbon number. However, the introduction of a condensed solvent phase that interacts with reactive intermediates complicates the free energy profile for catalytic reactions. For example, Bregante et al. showed H_2_O molecules rearrange to form structures that depend on the size of alkene reactants during epoxidations in zeolite pores.^29^ These rearrangements of H_2_O clusters confer significant excess enthalpic and entropic contributions, which reflect both the zeolite pore structure and polarity as well as direct interactions among H_2_O molecules and epoxidation transition states.^27^ Potts et al. clarified the balance between these interactions through comparisons between experimental activation barriers, grand canonical Monte Carlo simulations, and Born–Haber analyses that revealed the reorganization of H_2_O clusters cause enthalpic epoxidation barriers to increase with increased alkene chain length: these contributions modify rates by an order of magnitude for C_6_ to C_18_ alkenes.^27^ Similarly, Pfriem et al. demonstrated cyclohexanol dehydration rates increase linearly with increasing ionic strength between hydronium ion clusters ((H_3_O^+^)_*x*_) and charged intermediates for cyclohexanol dehydration.^33^ The authors also concluded adjacent (H_3_O^+^)_*x*_ exert destabilizing van der Waals forces that increase with increasing proximity, and these interactions offset the benefits of ionic interactions over Brønsted acidic MFI zeolites. The alkyl chain length of spectating molecules also affects the stabilities of reactive intermediates significantly *via* changes to the quantity and arrangement of intrapore species. For instance, Torres et al. demonstrated the alkyl chain length of *n*-alcohol (ROH) solvents affects their intrapore organization and the hydrogen bonding ability of ROH corresponds directly to alkene epoxidation rates.^28^ Taken together, the literature demonstrates that kinetics of reactions that occur in liquid-filled pores of zeolites sense interactions among the extended pore structure and reactive intermediates and the dense fluid within these environments represents a crucial component for mediating these interactions.^34^

Prior reports indicate that rates of epoxide ring-opening reactions depend upon the identity and structure of the ROH nucleophile whereas regioselectivities often remain constant. Yet, these studies conducted rate measurements in neat or highly concentrated solutions of the ROH reactant, which convolutes the effects of the intrinsic reactivity of the nucleophile (e.g., covalent, ionic effects) with excess properties of the solution and catalyst used. For example, Ogawa and Mori showed 1,2-epoxybutane (C_4_H_8_O) ring-opening rates increase from C_1_ to C_6_ primary ROH and regioselectivities remain fixed during reactions with Al-MOR zeolites at solution reflux.^35^ These authors also investigated reactions over Al-FAU zeolites: rates decreased from C_1_ to C_3_ before increasing from C_3_ to C_6_ and product regioselectivities to the terminal ether increased across the entire ROH range.^35^ Takeuchi et al. reported similar trends during ring-opening reactions of propylene oxide with primary ROH over Al-FAU: product yields obtained during a fixed period increased from C_1_ to C_4_ before decreasing from C_4_ to C_12_ while regioselectivities remained similar.^36^ The same authors observed ring-opening reactions with styrene oxide exhibited product yields that decreased across the entire range of ROH tested (C_2_ to C_6_) and regioselectivities did not change significantly.^36^ Finally, Deshpande et al. also reported increased rates and invariant product regioselectivities during epichlorohydrin ring-opening with C_1_ to C_4_ ROH over Sn-BEA zeolites.^18^ Recent work by our group demonstrated liquid-phase epoxide ring-opening over zeolites senses solvating effects from the acid type (Brønsted or Lewis), (SiOH)_*x*_ density, and fluid composition, which were not considered in explanations for effects of alcohol chain length.^17,23^ These studies demonstrated changes to the BEA* zeolite structure and fluid phase impart *G* ^ε^ contributions that cause rates to span 2 orders of magnitude. Taken together, prior works establish the sensitivity of epoxide ring-opening reactions to ROH chain length but do not explain the underlying solvating phenomena or distinguish among the attributes of the reactants and catalyst microenvironment responsible for rate and regioselectivity trends.

Here, we implicate the solvating phenomena and ROH attributes that govern the rates and regioselectivities of C_4_H_8_O ring-opening by varying the ROH nucleophile chain length (C_1_–C_6_) over Brønsted (Al-BEA) and Lewis acidic (Zr-BEA) *BEA zeolites in acetonitrile (CH_3_CN) solvent. Turnover rate measurements as functions of reactant concentrations follow similar dependencies regardless of ROH identity, which suggests all ROH nucleophiles and both acid types perform C_4_H_8_O ring-opening through similar mechanisms. Rates generally increase with increasing ROH chain length and do so to a lesser extent over Zr-BEA, which imply reactive intermediates possess coordination structures distinct from Al-BEA. Apparent activation enthalpies corrected for activity decrease linearly (i.e., become less positive) with increasing proton affinities for both materials, which demonstrates that rate increases stem primarily from the nucleophile strength of the ROH. However, interpretation of measured trends in apparent activation free energies (and entropies) strongly suggest the reorganization of solvent molecules to accommodate transition states and the interactions of transition states with zeolite pore structures also contribute to rate differences. Moreover, solvent mediated interactions with transition states predominantly dictate differences in regioselectivities, which appear most clearly in comparisons of regioselectivities achieved following the addition of H_2_O as a cosolvent. Regioselectivities differ more with the addition of H_2_O than for other changes in fluid composition examined, which implies the hydrogen bonds among solvated ROH provide the greatest differentiator among transition states that form either terminal alcohol or terminal ether products. These findings demonstrate the individual roles of the nucleophile strength for ROH reactants and hydrogen bonding among solvents and reactants on ring-opening kinetics in zeolite catalysts.

## 2 Methods and Materials

### 2.1 Catalyst Synthesis

Postsynthetic modifications of commercial Al-BEA-20 (TOSOH, lot #94HA6X92Y; Si/Al = 20) were used to remove Al atoms and incorporate Zr atoms into the zeolite framework. For Zr-BEA, thorough dealumination was achieved by thrice repeated treatments of parent Al-BEA-20 in HNO_3_ (70 wt %, Macron Chemicals, 20 cm^3^ g_zeolite_^–1^) at 433 K for 24 h. Between acid treatments, solids are recovered by vacuum filtration and rinsed with deionized H_2_O (18.2 MΩ cm, Elga Purelab Flex 2, 50 cm^3^ g_zeolite_^–1^). This process removes Al atoms from the *BEA framework by forming soluble Al­(NO_3_)_3_ complexes and repeating the treatment ensures a fully siliceous material (Si-BEA; Si/Al > 1200). For Al-BEA, partial dealumination was achieved by treating Al-BEA-20 in 1 M HNO_3_ at 433 K for 3 h, followed by vacuum filtration and rinses with deionized H_2_O. After acid treatments, wet solids were placed in a convection oven (Yamato, DKN602C) at 343 K to dry overnight. The dry zeolites were loaded into a quartz boat and placed into a three-zone horizontal furnace (Applied Test Systems, 3210), which was heated to 823 K (5 K min^–1^) and held for 6 h in flowing dry air (Airgas, Ultra Zero grade, 200 cm^3^ min^–1^) to remove residual H_2_O and organic molecules.

Zr atoms were incorporated into the siliceous zeolite by solid-state ion-exchange of zirconocene dichloride (Cp_2_ZrCl_2_; 97%, TCI) following previously reported procedures^19,37^ with minor changes. Briefly, Si-BEA and Cp_2_ZrCl_2_ were ground for 600 s with a mortar and pestle. The solid mixture was loaded into a quartz boat, placed in a three-zone horizontal furnace, heated to 823 K (2 K min^–1^) under an Ar atmosphere (Airgas, Ultra Zero grade, 200 cm^3^ min^–1^), and held for 8 h to decompose the zirconium precursor and to allow for Zr migration into the framework. After cooling to ambient temperature, the mixture was heated to 823 K (5 K min^–1^) in flowing air (200 cm^3^ min^–1^) to remove residual organic molecules. In line with previous reports with Sn materials,^38^ performing separate Ar and air treatments was observed to avoid extra-framework ZrO_2_ formation. All resulting Si-BEA, Al-BEA, and Zr-BEA powders appear white.

### 2.2 Catalyst Characterization

Zeolite crystallinity was examined using an X-ray diffractometer (Rigaku, MiniFlex600) with Cu Kα radiation source (λ = 1.54 Å) at ambient conditions using a Si zero-background holder. X-ray diffractograms (Figure S1) for all final and intermediate zeolites match established diffraction patterns of *BEA.

Metal contents were quantified by inductively coupled plasma optical emission spectroscopy (ICP-OES; PerkinElmer, Optima 8300) of zeolite powders. The metal loadings and Si to metal ratios for each zeolite are summarized in Table 1. Reported turnover rates are calculated using the metal contents from ICP-OES.

**Table 1:** Characterization of BEA Zeolite Catalysts

catalyst	metal wt %^a^	Si/metal ratio^a^	active metal %^b^	**ϕ**_IR_ ^c^
Al-BEAa	0.64	71	97 ± 4	1.44
Al-BEAb	0.54	82	106 ± 7	1.52
Zr-BEA	2.15	69	92 ± 9	1.26

a Determined with ICP-OES.

b Determined from in situ 1,2-diphenylethylenediamine site titrations.

c Calculated from infrared spectra of dehydrated zeolite samples.

The dispersion of Zr atoms was examined using diffuse reflectance UV–vis spectroscopy (DRUV–vis; Varian, Cary 5000; Internal DRA-2500) and Raman spectroscopy (Renishaw, InVia). For DRUV–vis measurements, Zr-BEA or zirconium oxide (ZrO_2_; Sigma-Aldrich, 5 μm 99% trace metals basis) samples were mixed with magnesium oxide (MgO; Sigma-Aldrich, 99.995%) at a 20:1 mass ratio, pure MgO was used as a background, and spectra were obtained between 200–800 nm. DRUV–vis spectra were converted to Tauc plots and leading edges were linearly extrapolated to the horizontal axis to determine the band gap of each material (Figure S2). The Zr band gap energy of Zr-BEA exceeds the measured band gap of bulk ZrO_2_, indicating Zr atoms remain highly dispersed. For Raman measurements, pressed zeolite pellets were placed under a 532 nm laser and spectra were collected under *ex situ* ambient conditions. A power density of ∼ 2 mW μm^–2^ (Gentec-EO, PRONTO-SI) was used, and 10 scans at 60 s accumulation time were averaged to yield the spectra in Figure S3. Raman spectra indicate the absence of features corresponding to Zr–O–Zr linkages, and together with band gap measurements, evidence that Zr atoms reside within the zeolite framework and not in oligomeric oxide clusters.

The coordination of Al atoms within the zeolite structure was evaluated with Raman spectroscopy (Renishaw, InVia) and ^27^Al single pulse direct excitation magic-angle spinning nuclear magnetic resonance spectroscopy (^27^Al MAS NMR; Bruker AVIII 400). Raman spectra were collected using an analogous procedure described above and confirm the absence of Al–O–Al bonds (i.e., like those of Al_2_O_3_ phases or intraporous clusters comprised of extraframework alumina, Al_*x*_O_*y*_) within the Al-BEA material. ^27^Al MAS NMR spectra used 4 mm rotors (Bruker, Zirconia body, Kel-F cap) packed with zeolite powders (∼50 mg), spun at 10 kHz, and maintained at 298 K. NMR spectra were obtained using a 0.6 μs pulse, 1 s delay, and 1024 scans. Al­(NO_3_)_3_ was used as a standard to calibrate chemical shifts. NMR spectra of ambient (Figure S4a) and hydrated (Figure S4b) Al-BEA show the majority of Al exists in tetrahedral coordination environments (∼54 ppm) and only 3–6% of the Al resides in octahedral coordination positions (∼0 ppm) (Section S1.4). Spectra of Zr-BEA (Figure S4) confirm the absence of measurable quantities of Al in the zeolite framework after a complete dealumination.

Relative densities of (SiOH)_*x*_ groups were measured using an infrared spectrometer (Bruker, Tensor 37) equipped with a liquid nitrogen-cooled HgCdTe detector, as described previously.^39^ The transmission cell was equipped with CaF_2_ windows, connected to a gas manifold, and temperature controlled to maintain a flowing Ar atmosphere (100 cm^3^ min^–1^) and a temperature of 573 K. The system was held at 573 K for 2 h with the intent to desorb H_2_O and other volatile compounds before acquiring background spectra (128 scans, 4 cm^–1^). Heating to 773 K did not significantly alter the spectra, confirming the dehydration of the zeolite (Figure S5). Spectra of an empty transmission cell with CaF_2_ windows was used as a background to account for infrared light absorption due to windows of the cell. Infrared spectra of zeolite pellets are shown in Figure S5, with vibrational features at 3300–3750 cm^–1^ and 1800–2100 cm^–1^ representing the ν­(O–H) of (SiOH)_*x*_ groups and ν­(Si–O–Si) overtones of the *BEA framework, respectively.^40,41^ The Al-BEA spectrum contains an additional peak corresponding to the Brønsted acid proton stretching mode (∼3600 cm^–1^),^42^ which is deconvoluted and removed from the ν­(O–H) area (Figure S6). Relative densities of (SiOH)_*x*_ are defined by the ratio of ν­(O–H) and ν­(Si–O–Si) areas in the term Φ_IR_:

$$\phi_{IR}=\frac{A_{\nu \left(O-H\right)}}{A_{\nu \left(Si-O-Si\right)}}$$ 1

Postsynthetically modified Al-BEA and Zr-BEA catalysts exhibit similar ϕ_IR_ values, which remain similar up to at least 773 K (Figure S5), indicating similar densities of (SiOH)_*x*_ and pore polarities.

The acid site character (Brønsted or Lewis) was evaluated by infrared spectra of adsorbed pyridine (C_5_H_5_N; Sigma-Aldrich, > 99%). Following the procedure described above, zeolite samples were pelletized, loaded into the spectrometer, and dehydrated at 573 K. The cell was then cooled to 393 K where a background spectrum was collected prior to introducing C_5_H_5_N. A syringe pump (Legato 100, KD Scientific) was used to introduce C_5_H_5_N into flowing Ar (100 cm^3^ min^–1^). Spectra collected after absorbance features reached steady state and then purged with Ar to remove physisorbed species. Analysis of the spectra in Section S1.6 show the presence of Brønsted and Lewis acid sites in Al- and Zr-BEA materials, respectively.

Two different Al-BEA materials were used in this study, and both materials give analogous characterization results and kinetic performance (Figures S8 and S9). The material denoted Al-BEAa in Table 1 was used for rate measurements at various reagent concentrations, and Al-BEAb was used to determine activation barriers, calorimetrically assessed enthalpies of adsorption, and the impacts of H_2_O cosolvent on rates. Taken together, the suite of characterization techniques discussed here indicate Al-BEA and Zr-BEA possess crystalline *BEA frameworks containing well-dispersed, primarily tetrahedral heteroatoms with similar densities of (SiOH)_*x*_ and distinct acid site character.

### 2.3 Turnover Rate Measurements

Turnover rates for 1,2-epoxybutane (C_4_H_8_O; TCI, > 99.0%) alcoholysis with methanol (MeOH; Sigma-Aldrich, ≥ 99.9%), ethanol (EtOH; Sigma-Aldrich, HPLC, ≥ 99.5%), 1-butanol (1-BuOH; Sigma-Aldrich, 99.9%), and 1-hexanol (1-HxOH; Thermo Scientific, 99%) in acetonitrile (CH_3_CN; Sigma-Aldrich, HPLC Plus, ≥ 99.9%) were measured using batch reactors consisting of three-necked, 100 cm^3^ round-bottomed flasks equipped with reflux condensers cooled with running H_2_O (295 K) and submerged in a temperature-controlled H_2_O bath on a hot plate (Thermo Scientific, Cimarec+ SP88857100). Benzene (Sigma-Adrich, analytical standard) in the case of MeOH, EtOH, and 1-HxOH or octane (Sigma-Aldrich, analytical standard) in the case of 1-BuOH were used as internal standards. In a typical reaction, 30 cm^3^ of reagents, solvent CH_3_CN (and H_2_O for cosolvent measurements), and internal standard were combined in the reactor flask and stirred (700 rpm, 0.5 h) to equilibrate before taking an initial aliquot (∼0.5 cm^3^). Reactions were initiated by adding zeolite (15–50 mg), and aliquots were taken as a function of time and filtered (Tisch Scientific, 0.22 μm, SF14704) into sealed vials (2 cm^3^).

The concentrations of epoxide, internal standard, and products (terminal ether and terminal alcohol) were quantified with a gas chromatograph (Agilent, 8890) equipped with a flame ionization detector, liquid autosampler (Agilent, G4513A), and HP-1 column (Agilent, 19091Z-236). Product assignments and calibration factors were determined using commercially obtained samples of the methanol terminal ether (1-methoxy-2-butanol; TCI, > 93%) and terminal alcohol (2-methoxy-1-butanol; TCI, > 98%) products. Higher chain length alcohol product peak assignments were assumed to follow the order of methanol products and calibration factors were estimated using an effective carbon number method (Section S3). Carbon selectivity to alcoholysis products is greater than 90% for all measurements and residual H_2_O in the CH_3_CN solvent was estimated to be 0.022 M using ^1^H NMR (Section S16). Two minor side products appear with similar GC elution times to MeOH and EtOH products and are observed in all chromatograms regardless of alcohol identity or presence. Comparisons to known standards excluded both side products as representing 1,2-butanediol, dibutyl ether, butanal, or butanone. We previously attributed these peaks to epoxide oligomers or epoxide reactions with trace species (e.g., CO_2_).^23^ All rates were measured at differential conversion (<10%; with the vast majority of measurements <5%) and regioselectivities are not a strong function of conversion (Section S17).

Turnover rate values were determined by taking the derivative of a second-order polynomial fit to turnover numbers of primary products as a function of time with fixed zero turnover at time equal to zero. Uncertainty from replicate experiments was ∼ 15% for all measurements. Site titration experiments with (1R,2R)-(+)-1,2-diphenylethylenediamine (DPED; Sigma-Aldrich, 97%) probed the quantity of active sites in Al-BEA and Zr-BEA catalysts by blocking metal atoms from performing chemistry. Active metal percentages are summarized in Table 1 and a full analysis of the titration is provided in Section S4. Hot filtration of a large reaction volume (∼5 cm^3^ taken ∼ 600 s after initiation with catalyst and stirred in a separate vessel) was used to determine if framework metal atoms are removed from the *BEA framework and become homogeneous complexes active for ring-opening. Aliquots were taken from both vessels as a function of time and analyzed to confirm product concentrations change negligibly without the zeolite catalyst (Figure S14). Turnover rates measured for Si-BEA created through dealumination are at least 1 order of magnitude lower than Zr-BEA turnover rates,^17,23^ demonstrating residual Al do not contribute meaningfully to measured rates in Zr-BEA. Rate measurements do not depend on Al or Zr metal loading (Figure S15), satisfying the Madon–Boudart criterion for mass transport.^43^ Additionally, rate measurements at limiting concentrations of [C_4_H_8_O] and [ROH] show combinations of zero- and first-order dependencies (Section 3.1). These dependencies imply a lack of mass transport limitations as reactant concentrations in a mass-transfer limited material would be sublinear due to concentration gradients.^44^ Collectively, these experiments suggest C_4_H_8_O ring-opening occurs solely at zeolite framework-bound metal atoms without mass transport limitations.

### 2.4 Liquid-Phase Reactant Adsorption Enthalpies

The heats of ROH or C_4_H_8_O adsorptions into Al-BEA and Zr-BEA catalysts were measured with isothermal titration calorimetry (ITC; TA Instruments, NanoITC SV). Notably, our previous contributions used a low volume instrument to conduct titrations rather than the present standard volume instrument.^17,28,45,46^ Nonetheless, analyses herein follow an analogous set of steps reported recently which avoid harsh HNO_3_ and NaOH.^17^ Briefly, the sample cell was cleaned by flowing a cleaning solution (1000 cm^3^ of 2 vol% detergent (Micro90) in deionized H_2_O) through the cell before flowing 2000 cm^3^ of deionized H_2_O to remove residual detergent. The sample cell, reference cell, and 0.1 cm^3^ ITC syringe were then filled with 1.25 cm^3^ or 0.1 cm^3^ of deionized H_2_O and assumed clean when the H_2_O–H_2_O titration released negligible amounts of heat (±3 μJ per 2.5 μL injection; example in Figure S29).

In a typical experiment, ∼ 10–30 mg of zeolite was suspended in 1.8 cm^3^ CH_3_CN by sonication for 30 min. Part of the zeolite slurry was loaded into the sample cell (1.25 cm^3^) with the remaining slurry being evaporated and dried catalyst weighed to calculate the mass of zeolite that entered the cell. The reference cell was filled with 1.25 cm^3^ of CH_3_CN and the 0.1 cm^3^ ITC syringe was filled with a 0.005 M solution of reactant in CH_3_CN. Titrations were conducted at reaction temperature (308 K) and while mixing at 250 rpm. Adsorption enthalpies were calculated by averaging integrated heats released upon injecting 0.0025 cm^3^ of titrant at low active site coverages (<0.2 mol titrant (mol metal)^−1^). In this regime, constant heats imply measured enthalpies are isosteric.^46^

## 3 Results and Discussion

### 3.1 Epoxide Alcoholysis Rates Suggest Analogous Mechanisms

Turnover rates for C_4_H_8_O alcoholysis measured across a range of fluid compositions and alcohol identities depend on the concentration of alcohol (Figure 1) and epoxide (Figure 2). Rates increase linearly with [ROH] and depend weakly on [C_4_H_8_O] at sufficiently low alcohol excess ([ROH]:[C_4_H_8_O] < 80). Similarly, rates become linear in [C_4_H_8_O] and constant with changes in [ROH] when [ROH]:[C_4_H_8_O] exceed 300 for most alcohols. However, Figure 1 shows rates over Al-BEA for methanol and ethanol do not approach constant values with [ROH] at the conditions evaluated. We have previously shown turnover rates on Al-BEA linearly depend on [C_4_H_8_O] and are constant with [ROH] when [ROH]:[C_4_H_8_O] values exceed 5000.^17^ Therefore, differences in the position at which rates transition between dependency regimes depend on the combination of ROH and catalyst and likely depend on the relative magnitudes of rate constants (*vide infra*). These rate relationships suggest C_4_H_8_O-derived surface species dominate at low [ROH]:[C_4_H_8_O] and ROH-derived species dominate at sufficiently high [ROH]:[C_4_H_8_O]. Further, these observations (Figures 1 and 2) suggest epoxide ring-opening proceeds *via* steps that do not require both reactive species to bind simultaneously to the same active site (i.e., an Eley–Rideal mechanism).

**Figure 1:**
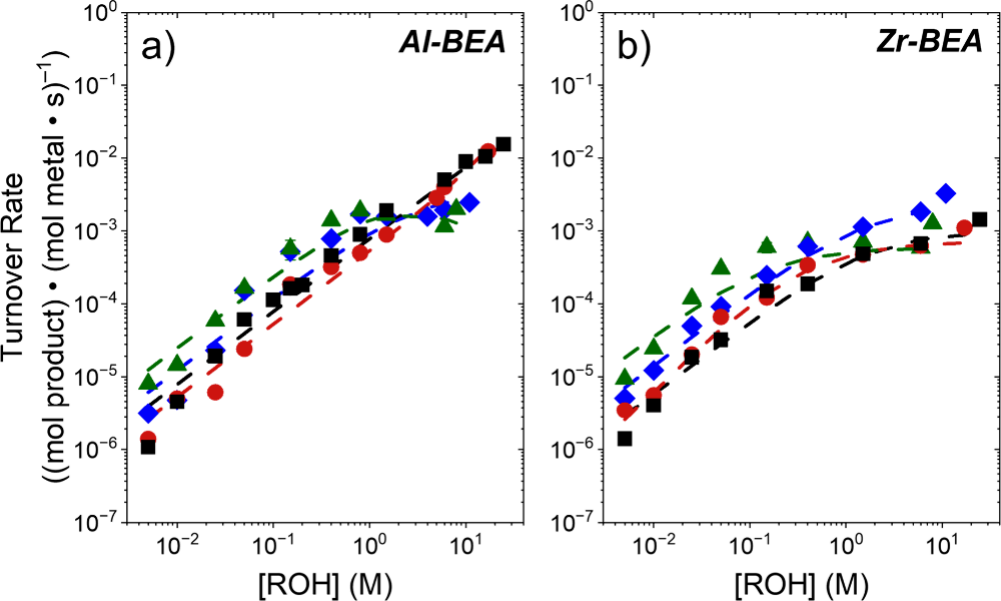
Turnover rates for C_4_H_8_O alcoholysis as functions of methanol (MeOH, black square), ethanol (EtOH, red circle), 1-butanol (1-BuOH, blue diamond), and 1-hexanol (1-HxOH, green triangle) concentrations over (a) Al-BEA and (b) Zr-BEA (0.005 M C_4_H_8_O, CH_3_CN solvent, 308 K). Dashed lines represent global fits of the combined data from Figures 1 and 2 to eq 3. Methanol Al-BEA adapted from previous work.^17^ Error bars represent the standard deviation of replicate experiments.

**Figure 2:**
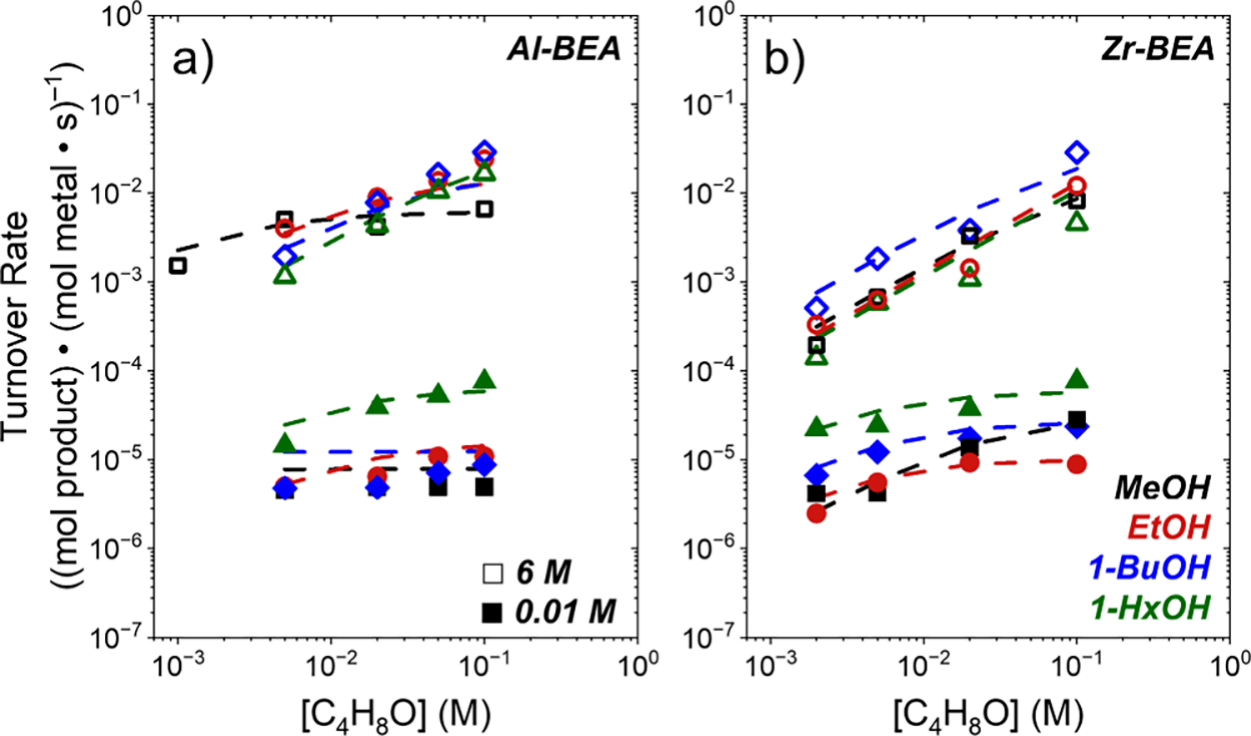
Turnover rates for C_4_H_8_O alcoholysis as functions of C_4_H_8_O concentrations at 0.01 M (filled) and 6 M (hollow) methanol (MeOH, black square), ethanol (EtOH, red circle), 1-butanol (1-BuOH, blue diamond), and 1-hexanol (1-HxOH, green triangle) over (a) Al-BEA and (b) Zr-BEA (CH_3_CN solvent, 308 K). Dashed lines represent global fits of the combined data from Figures 1 and 2 to eq 3. Methanol Al-BEA adapted from previous work.^17^

Similarities in reaction orders for Al-BEA and Zr-BEA across the range of C_1_–C_6_ primary alcohols indicate these materials and alcohol nucleophiles perform epoxide alcoholysis through analogous sets of elementary steps. Scheme 2 depicts a general reaction mechanism consistent with our previous proposal for this reaction.^17,23^ Briefly, an unoccupied active site reversibly adsorbs CH_3_CN (step 1), C_4_H_8_O (step 2), or ROH (step 3). The complementary reagent reacts with the surface-bound species (ROH for C_4_H_8_O*, C_4_H_8_O for ROH*; * denotes surface species) in a kinetically relevant step (steps 4 and 5, respectively) to form either one of the two possible regioisomers. The terminal ether (TE) and terminal alcohol (TA) products then desorb (steps 6 and 7) to recover the bare active site and complete the catalytic cycle. Presumably, both the TE and TA products derive from similar surface intermediates due to the individual turnover rates following nearly the same [C_4_H_8_O] and [ROH] dependencies (Section S7). Consequently, we denote the rate constants for these parallel pathways as *k*_*x,y*_, where *x* denotes the step and *y* denotes the product formed (e.g., *k*_4, *TE*_).

**Scheme 2:**
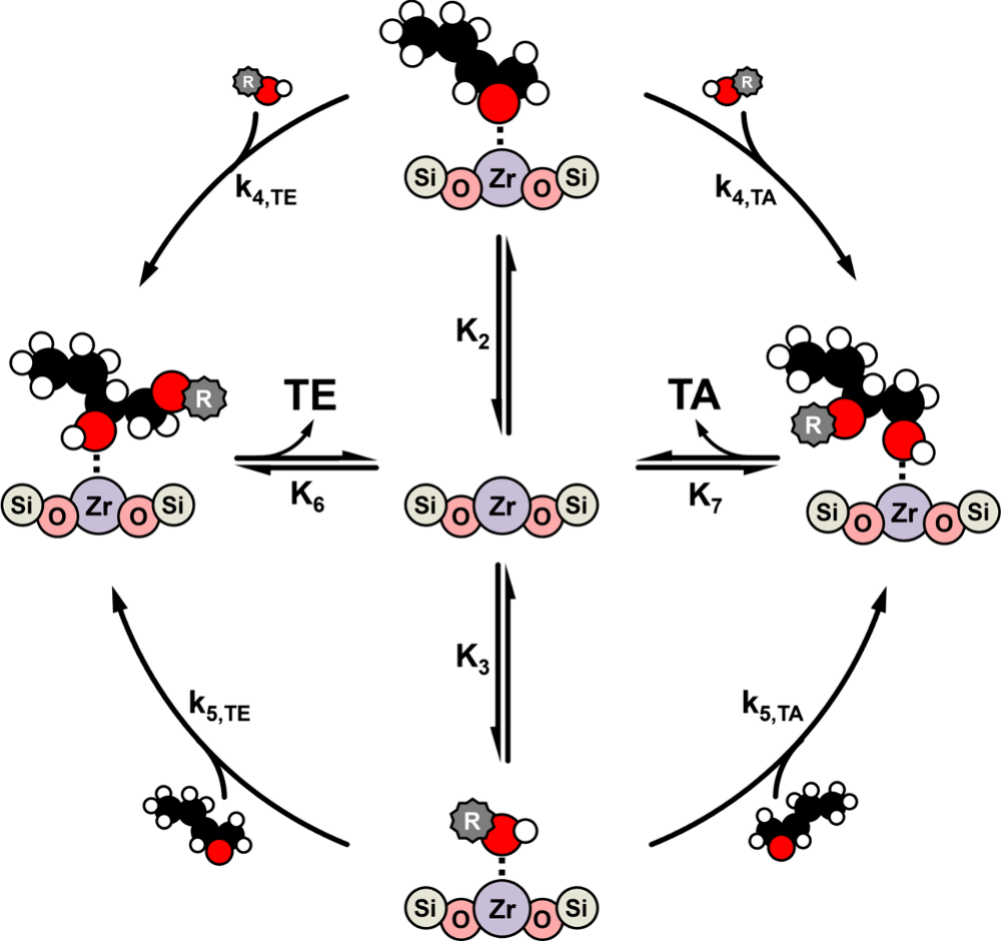
Proposed Reaction Mechanism for C_4_H_8_O Ring-Opening with Alcohols over Lewis Acidic Zr-BEA Catalysts^a^

a Brønsted acidic Al-BEA catalysts proposed to follow a related mechanism that involves proton-transfer steps (Scheme S1). For clarity, we omit reversible adsorption of CH_3_CN (step 1), spectating solvent molecules, and the possibility of adsorbed species to bind in dissociative configurations—though these binding modes may be possible. All adsorption steps are assumed to be reversible (denoted by a double arrow), while a single arrow represents an irreversible step.

Epoxide ring-opening rates (*r*_*RO*_) equal the sum of reactions that involve either bound C_4_H_8_O* (*k*_4_) or bound ROH* (*k*_5_) to form the regioisomer products:

$$r_{RO}=r_{4}+r_{5}=k_{4}\left[C_{4}H_{8}O^{*}\right]\left[ROH\right]+k_{5}\left[C_{4}H_{8}O\right]\left[ROH^{*}\right]$$ 2

A general turnover rate expression emerges after applying the pseudo steady state hypothesis and a site balance (Section S8 for full derivation). Simplifying the general turnover rate expression to include relevant surface species yields:

$$\frac{r_{RO}}{\left[L\right]}=\frac{\frac{k_{2}k_{4}\left[C_{4}H_{8}O\right]\left[ROH\right]}{k_{-2}+k_{4}\left[ROH\right]}+\frac{k_{3}k_{5}\left[C_{4}H_{8}O\right]\left[ROH\right]}{k_{-3}+k_{5}\left[C_{4}H_{8}O\right]}}{\left(1+K_{1}\left[CH_{3}CN\right]+\frac{k_{2}\left[C_{4}H_{8}O\right]}{k_{-2}+k_{4}\left[ROH\right]}+\frac{k_{3}\left[ROH\right]}{k_{-3}+k_{5}\left[C_{4}H_{8}O\right]}\right)}$$ 3

where the numerator contains two groups of terms that signify products formed by reaction of either a C_4_H_8_O*- or ROH*-derived surface intermediate (left-hand and right-hand collections of variables, respectively) and the series of terms within the denominator represent the numbers of unoccupied sites, CH_3_CN*, C_4_H_8_O*, and ROH* surface species, respectively. eq 3 simplifies further when a single surface species saturates active sites. When C_4_H_8_O* species prevail and reactions proceed via an ROH molecule reacting with the C_4_H_8_O*-derived species (such that *r*_*5*_ approaches zero), the expression reduces to the form:

$$\frac{r_{RO}}{\left[L\right]}=k_{4}\left[ROH\right]$$ 4

Similarly, surfaces dominated by ROH* facilitate reactions that occur primarily by combination of C_4_H_8_O with an ROH*-derived surface complex (*r*_4_ = 0) yields:

$$\frac{r_{RO}}{\left[L\right]}=k_{5}\left[C_{4}H_{8}O\right]$$ 5

Note, these assumptions arise from coupled relationships between rate constants and the concentration regime in which each surface species dominates (Section S8). For instance, epoxide molecules bind 140 kJ mol^–1^ (Al-BEA) or 109 kJ mol^–1^ (Zr-BEA) more exothermically than 1-hexanol, the most exothermic ROH (Figure S21). Therefore, the rate constants for epoxide adsorption likely exceed those for alcohol adsorption (*k*_2_ ≫ *k*_3_), leading to the C_4_H_8_O* species most likely reacting directly with fluid-phase ROH. Further, ROH adsorption enthalpies become more exothermic with increasing ROH chain length over both Al- (−0.2 ± 0.1 for methanol to −3.6 ± 0.4 kJ mol^–1^ for 1-hexanol) and Zr-BEA (−0.3 ± 0.1 for methanol to −4.8 ± 0.4 kJ mol^–1^ for 1-hexanol). Similarly, the adsorption enthalpies of gas-phase *n*-alkanes over Brønsted acid zeolites increase in exothermicity with increasing alkyl chain length due to van der Waals interactions with zeolite pore structures.^47,48^ More-exothermic binding of larger ROH species agrees with gas-phase proton affinities for ROH species (-ΔH_rxn, gas_ ; ROH + H^+^ → ROH_2_^+^) which increase with increasing ROH chain length (754–799 kJ mol^–1^; Table S2).^49,50^ Together, trends in adsorption enthalpies and proton affinities suggest 1-HxOH competes more effectively for the Brønsted proton and benefits to a greater extent from zeolite pore interactions to saturate active sites at lower [ROH] than in the case of MeOH, as shown in Figure 1. Similar phenomena seem likely for Lewis acid active sites, however, proton affinities do not provide a direct assessment for adsorption thermodynamics when proton transfer does not occur upon binding.

eqs 4 and 5 predict that turnover rates for C_4_H_8_O alcoholysis depend linearly on the concentration of one reactant and do not depend on the other in the limit that either C_4_H_8_O*- or ROH*-derived species saturate sites. Measured turnover rates (Figures 1 and 2) agree with these predictions for all alcohols examined. Furthermore, the dashed lines in Figures 1 **and** [Fig fig2] represent best fits of the combined data set for each ROH to eq 3 (details in Section S8) and agree with experimental data (Figure S20). These comparisons suggest reactions between C_4_H_8_O and C_1_–C_6_ primary alcohols over Al-BEA and Zr-BEA zeolites share analogous reaction mechanisms and collections of surface intermediates. Mathematically equivalent rate expressions also describe C_4_H_8_O ring-opening turnover rates for epoxide ring opening upon Al-, Zr-, Ti-, and Sn-BEA zeolites with varied (SiOH)_*x*_ densities with methanol,^17,23^ which indicates the mechanisms described here for C_1_–C_6_ primary alcohols extend to framework substituted zeolites with distinct metal identities and polarity. Finally, hydrolysis of C_4_H_8_O (i.e., reaction with H_2_O as the nucleophile) follows similar trends with reagent concentrations (Figure S22), which implies water reacts by a mechanism similar to Scheme 2. Although the mechanisms of these reactions follow similar pathways, changes to the reactant structure do lead to differences in rates and regioselectivities due to solvating interactions within zeolite pores (*vide infra*).

### 3.2 Consequences of Alcohol Identity on Epoxide Alcoholysis Rates and Energetics

Epoxide ring-opening turnover rates depend on the identity of the ROH and typically increase with increasing alcohol chain length. Figure 3a shows that turnover rates for ring-opening with 1-HxOH are 6- (Al-BEA) to 4-times (Zr-BEA) greater than for MeOH (0.005 M C_4_H_8_O, 0.15 M ROH, 308 K), and these trends agree with previous reports of alcoholysis of aliphatic epoxides in solutions of neat alcohols over Brønsted acidic MOR^35^ and FAU.^35,36^ However, the reactions performed here (Figures 1 **-** [Fig fig3]) contain large mole fractions of CH_3_CN as a cosolvent, and thus, the thermodynamic activities of the reactants differ with the identities of the alcohol reactant. Calculations of intrinsic rate constants using the activity of the alcohol reagent (*a*_*ROH*_, determined using activity coefficients γ_*C*_4_ *H*_8_ *O*_ and γ_*ROH*_; detailed discussion in Section S12) account for these complications. Consequently, values for the intrinsic rate constant for ring-opening on C_4_H_8_O* saturated sites (*k*_*4*_) derive from:

$$k_{4}=\frac{r_{RO}}{\left[L\right]a_{ROH}}$$ 6

**Figure 3:**
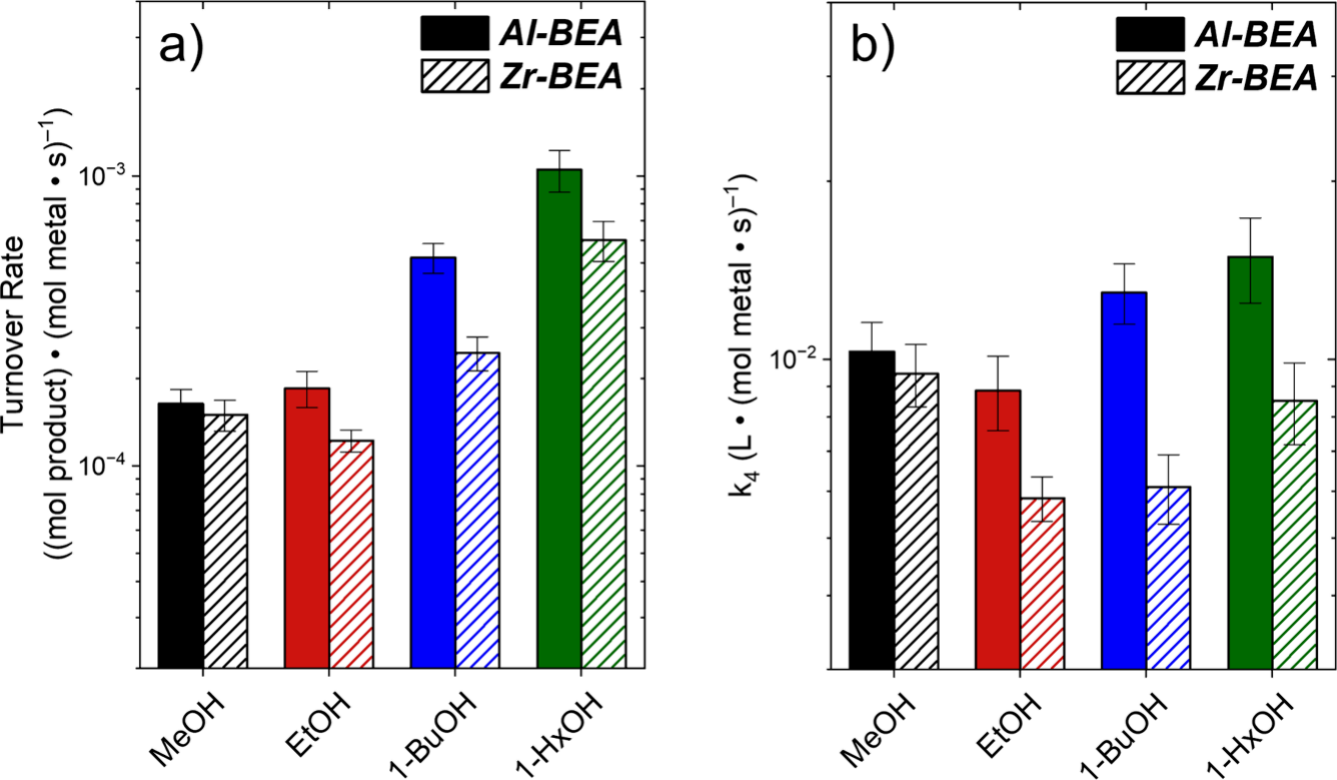
Ring-opening (a) turnover rates and (b) intrinsic rate constants (*k*_4_) for conversion of C_4_H_8_O as functions of primary alcohol identity over Al-BEA (solid) and Zr-BEA (hashed) (0.005 M C_4_H_8_O, 0.15 M ROH, CH_3_CN solvent, 308 K). Alcohol activities used in calculating rate constants were estimated using UNIFAC (Section S12). Error bars represent the standard deviation of replicate experiments.

An analogous form of this equation emerges for ROH* dominated sites, in which epoxide activities replace alcohol activities. Figure 3b shows that calculated values of *k*_*4*_ (from eq 6) change nonmonotonically with ROH chain length for both Al-BEA and Zr-BEA. While *a*_*ROH*_ account for a large fraction of the changes in turnover rates, statistical analyses (Section S12) and large changes in reaction barriers (*vide infra*) suggest intrapore interactions additionally affect the kinetics of epoxide consumption. These intrapore interactions evidently result in a balance between opposing effects among alcohol species (e.g., inductive and steric) to yield the nonmonotonic trends observed in Figure 3b.^51,52^

To probe the molecular phenomena responsible for these trends in rate constants, these rate constants are restated in the form suggested by Eyring that reflects activation free energies:

$$k_{4}=\frac{k_{B}T}{h}\exp\left(-\frac{\Delta G_{4}^{‡}}{RT}\right)$$ 7

where *k*_*B*_, *h*, and *R* are the Boltzmann, Planck, and universal gas constants, respectively, and *T* is temperature (K). Scheme 3 molecularly portrays the apparent activation free energy (Δ*G*_4_^‡^) obtained in the limit that C_4_H_8_O* species saturate all sites, which corresponds to the differences among the following terms:

$$\Delta G_{4}^{‡}=G_{4}^{‡}-G_{C_{4}H_{8}O^{*}}-G_{ROH}$$ 8

where this relationship shows Δ*G*_4_^‡^ depends on the free energies of the transition state (*G*_4_^‡^), bound C_4_H_8_O* (*G*_*C*_4_ *H*_8_ O*_), and liquid-phase ROH (*G*_*ROH*_). Each state experiences intermolecular forces with solvating molecules (i.e., CH_3_CN and spectating ROH) or the zeolite pore. Restating the free energy terms as the combination of the standard state (*G* ^0^) contributions that stem from covalent interactions and excess (*G* ^ε^) contributions, which arise from solution nonidealities and interactions at the zeolite pore wall, decouples these factors in further analysis:^32^

$$\Delta G_{4}^{‡}=\left(G_{4}^{‡,0}+G_{4}^{‡,\varepsilon }\right)-\left(G_{C_{4}H_{8}O^{*}}^{0}+G_{C_{4}H_{8}O^{*}}^{\varepsilon }\right)-\left(G_{ROH}^{0}+G_{ROH}^{\varepsilon }\right)$$ 9

**Scheme 3:**
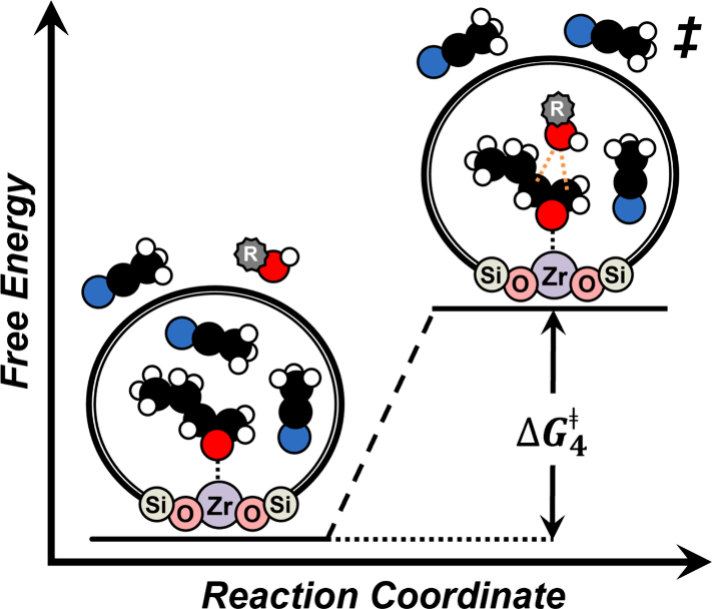
Reaction coordinate diagrams for C_4_H_8_O ring-opening with alcohols over Zr-BEA catalysts when C_4_H_8_O* intermediates saturate active sites^a^

a The activation free energy (Δ*G*_4_^‡^) reflects changes in stability caused by fluid-phase ROH entering the zeolite pore and reacting with C_4_H_8_O* species to form a transition state that forms a C–O bond. An analogous reaction coordinate involving proton transfer is expected for Al-BEA catalysts.

Analogous forms of eqs 8 and 9 exist for activation enthalpy (Δ*H*_4_^‡^) and entropy (Δ*S*_4_^‡^).

Figure 4 shows apparent activation enthalpies (Δ*H*_4_^‡^) and entropies (Δ*S*_4_^‡^) calculated using *a*_*ROH*_ (detailed calculation in Section S13) decrease linearly as the proton affinity of the reactant ROH increases. In Al-BEA, values of Δ*H*_4_^‡^ decrease by 12 ± 2 kJ mol^–1^ from MeOH to 1-HxOH. This trend suggests that stronger alcohol-epoxide linkages^53^ formed in the transition state contribute to lower enthalpic barriers (captured in *H*_4_^‡,0^), although molecular interpretation of barrier terms suggests changing the ROH chain length may also alter interactions with solvent molecules and the zeolite pore structure (i.e., through *H*_4_^‡, ε^; Section S14). Concomitant decreases in Δ*S*_4_^‡^ (35 ± 6 J mol^–1^ K^–1^) reflect the combined loss of entropy associated with ROH coordinating to form the transition state (captured in *S*_4_^‡,0^) as well as the associated reorganization of the molecules that solvate the C_4_H_8_O* intermediate and zeolite pore interactions with the transition state (combined in *S*_4_^‡, ε^). Values of both Δ*H*_4_^‡^ and Δ*S*_4_^‡^ vary across a greater range within Zr-BEA (33 ± 7 kJ mol^–1^ and 111 ± 21 J mol^–1^ K^–1^), which indicates reactions at Lewis acid sites respond more sensitively to differences in the structure of the nucleophile. Intuition suggests proton affinities carry lesser significance during reactions upon Lewis acid sites, yet, alcohol mediated ring-opening may proceed by intermolecular proton transfer.^18,54,55^ Thus, Figure 4 suggests either intermolecular proton transfers hold greater energetic significance than proton transfer steps involving the catalyst surface or significant differences exist in the solvation of transition states (extended discussion in Section S14). Quantitative comparisons among values of Δ*H*_4_^‡^ and Δ*S*_4_^‡^ for these materials demonstrate that the enthalpic benefits associated with greater proton affinities (and related changes in reactant structure) confer a greater impact on values of Δ*G*_4_^‡^ than entropic effects such that turnover rates (and rate constants) increase with chain length at the conditions examined here (290–323 K).

**Figure 4:**
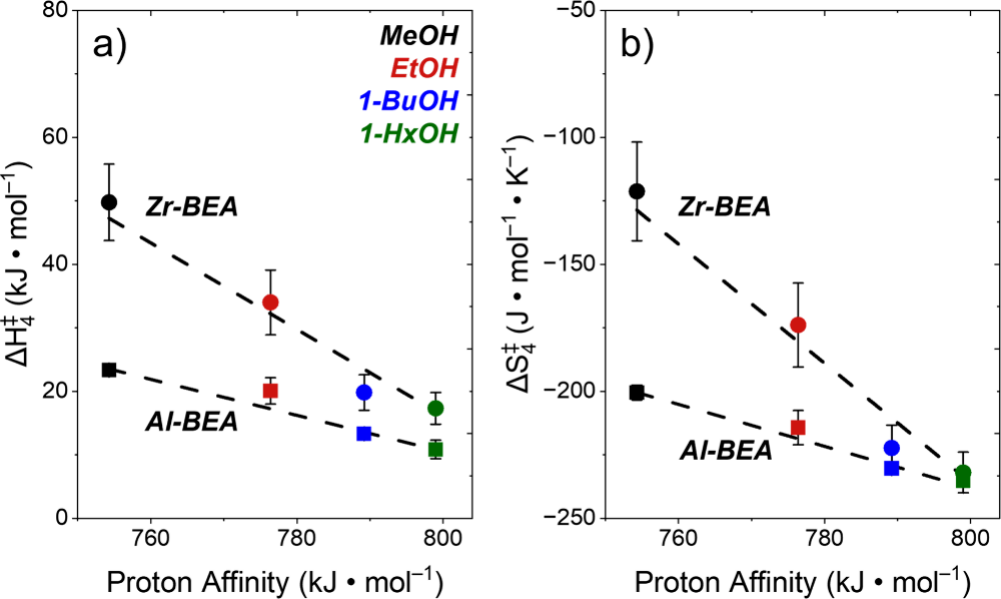
Values of apparent activation (a) enthalpies (Δ*H*_4_^‡^) and (b) entropies (Δ*S*_4_^‡^) for C_4_H_8_O ring-opening over Al-BEA (squares) and Zr-BEA (circles) as functions of primary alcohol proton affinity when C_4_H_8_O-derived intermediates saturate sites (0.005 M C_4_H_8_O, 0.15 M ROH, CH_3_CN solvent, 290–323 K).

The enthalpic and entropic significance of interactions between solvent molecules, zeolite pore structures, and reactive intermediates and transition states depend strongly on the fluid composition and the associated mechanistic regime. In the following section, the outcomes of these interactions on product distributions are investigated.

### 3.3 Ring-Opening Regioselectivities Depend on the Extent of Hydrogen-Bonded Alcohol Clusters

The transition states to form each regioisomer product may sense solvation differently, and consequently, solvating interactions within the confines of zeolite pores could offer opportunities to manipulate regioselectivities. Rate differences between ROH primarily reflect changes in *G* ^‡^ values, and analogous reasons indicate that *G*_4_^‡^ specific to each regioisomer distinguish turnover rates of formation for the products and determine regioselectivities. Scheme 4 depicts the transition states for these parallel reaction pathways and the distinct sensitivity of each to excess contributions.

**Scheme 4:**
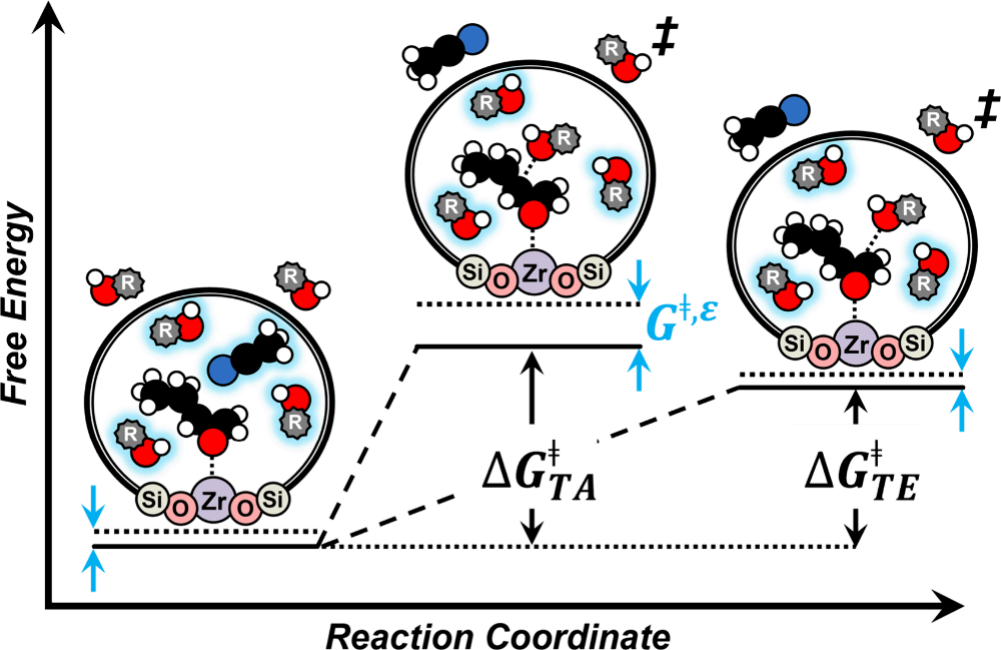
Proposed Reaction Coordinate Diagram for Terminal Alcohol (TA) and Terminal Ether (TE) C_4_H_8_O Ring-Opening Products over Zr-BEA^a^

a An analogous diagram is expected for Al-BEA. Conditions of an epoxide MARI are depicted as well as qualitative estimations of excess free energies (*G* ^‡,ε^) resulting from interactions by spectating solvent molecules (blue highlights) and the zeolite pore structure.

Ring-opening regioselectivity defined in the form of a rate ratio allows for straightforward assessment of the differences in free energies for the transition states of the parallel reaction pathways:

$$\beta =\frac{r_{TE}}{r_{TA}}=\exp\left(\frac{G_{TA}^{‡}-G_{TE}^{‡}}{RT}\right)=\exp\left(\frac{G_{TA}^{‡,0}-G_{TE}^{‡,0}}{RT}\right)\cdot \exp\left(\frac{G_{TA}^{‡,\varepsilon }-G_{TE}^{‡,\varepsilon }}{RT}\right)$$ 10

where the rates to form the TE and TA possess identical dependence on reactant concentrations (Section S7) and form from identical reference states in the catalytic cycle.

Figure 5 shows β values for C_4_H_8_O ring-opening with most alcohols remain nearly constant with changes in fluid composition represented by values of [ROH]:[C_4_H_8_O] that span nearly 5 orders of magnitude and capture both C_4_H_8_O* and ROH* kinetic pathways. Over Al-BEA, the average values of β (β_*avg*_) approach unity (e.g., 1.0 ± 0.2 for EtOH; Table 2) and imply the protonated transition states for both regioisomers participate in similar interactions with active sites (i.e., *G*_*TA*_^‡,0^ – *G*_*TE*_^‡,0^ ≪ *RT*) or solvent molecules and zeolite pore structures (i.e., *G*_*TA*_^‡, ε^ – *G*_*TE*_^‡, ε^ ≪ *RT*). In the case of Zr-BEA, β_*avg*_ values remain systematically higher than Al-BEA analogs (e.g., 1.5 ± 0.3 for EtOH; Table 2) and reflect more favorable interactions of the less sterically hindered TE over TA transition states with active sites, solvent molecules, or zeolite pores. Increasing the ROH chain length marginally decreases both the values of β_*avg*_ and the deviation from those values across the range of [ROH]:[C_4_H_8_O] explored (statistical discussion in Section S15). These data imply transition states of larger ROH species weakly sense zeolite pore interactions (e.g., van der Waals forces) in favor of the TA product yet do so to a greater extent than interactions related to the solvent (e.g., hydrogen bonding). Therefore, increasing the ROH chain length alone is an ineffective strategy to modify ring-opening regioselectivities.

**Figure 5:**
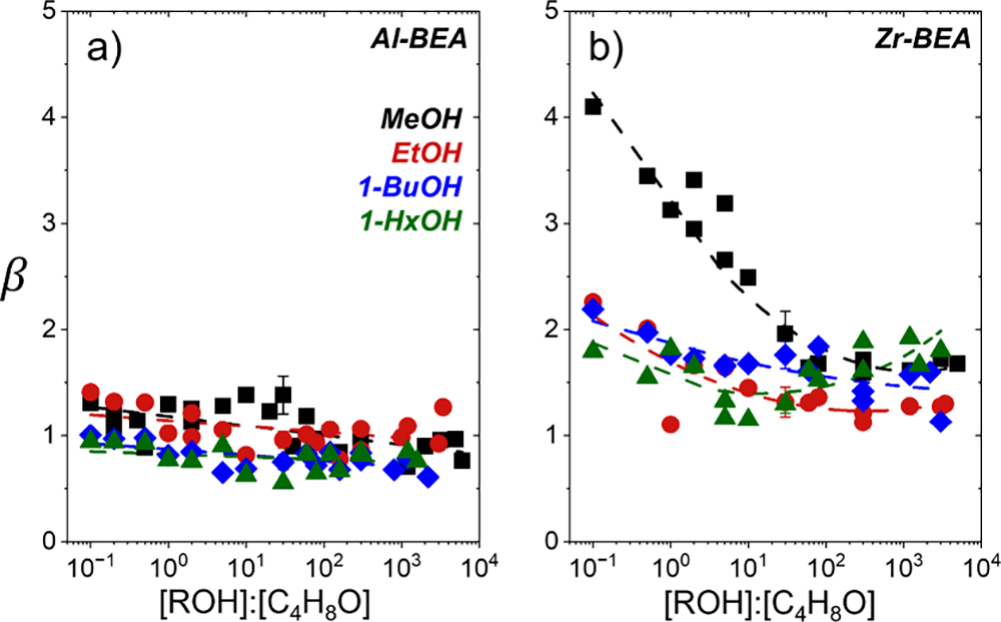
Regioselectivity β values for C_4_H_8_O alcoholysis with methanol (MeOH, black square), ethanol (EtOH, red circle), 1-butanol (1-BuOH, blue diamond), and 1-hexanol (1-HxOH, green triangle) as functions of alcohol-to-epoxide concentration ratio over (a) Al-BEA and (b) Zr-BEA (CH_3_CN solvent, 308 K). Methanol Al-BEA adapted from previous work.^17^ Error bars represent the standard deviation of replicate experiments. Percentage-based regioselectivities are shown in Figure S24.

**Table 2:** Average β Values for C_4_H_8_O Alcoholysis over Al-BEA and Zr-BEA

ROH	Al-BEA^a^	Zr-BEA^a^
MeOH	1.0 ± 0.2	2.4 ± 0.8
EtOH	1.0 ± 0.2	1.5 ± 0.3
1-BuOH	0.8 ± 0.1	1.6 ± 0.2
1-HxOH	0.8 ± 0.1	1.6 ± 0.2

a Average β values and standard deviation error calculated from Figure 5.

Previous reports suggest hydrogen bonding interactions of solvent molecules with transition states offer opportunities to influence regioselectivities.^5,6^ Methanol, with the greatest hydrogen bonding ability and smallest van der Waals volume of the ROH tested (Table S2), yields the greatest changes to β values across [ROH]:[C_4_H_8_O] over Zr-BEA compared to other ROH in Figure 5b. However, hydrogen bonding ability decreases with increasing ROH chain length and makes direct comparisons difficult. Thus, the role of hydrogen bonding in determining β values was examined through the addition of small fractions of H_2_O to reactions (Figure 6 **)**.

**Figure 6:**
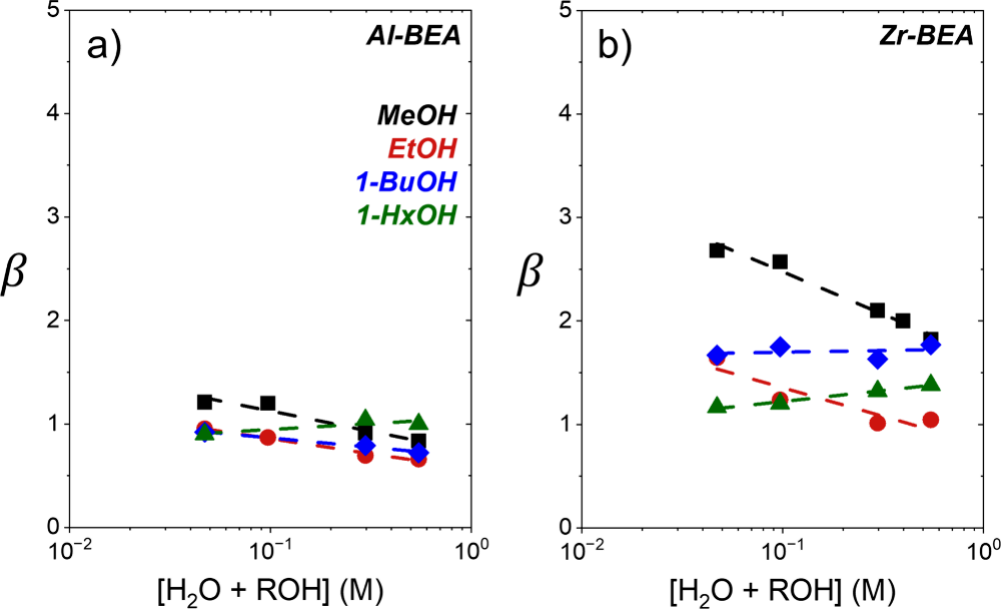
C_4_H_8_O ring-opening regioselectivity β values as functions of total hydrogen-bonding species concentration with methanol (MeOH, black square), ethanol (EtOH, red circle), 1-butanol (1-BuOH, blue diamond), and 1-hexanol (1-HxOH, green triangle) over (a) Al-BEA and (b) Zr-BEA (0.005 M C_4_H_8_O, 0.025 M ROH, 0–0.5 M intentional H_2_O, CH_3_CN solvent, 308 K). Adventitious H_2_O from the CH_3_CN solvent is estimated to be 0.022 M (Section S16) and is accounted for in the *x*-axis.

Figure 6 shows β values differ slightly for reactions conducted in the presence of cosolvating H_2_O, which indicates hydrogen bond interactions influence regioselectivity. H_2_O may react with C_4_H_8_O-derived intermediates to form 1,2-butanediol (Section S11), but hydrolysis turnover rates remain 100-times slower than alcoholysis turnover rates and do not impact the following analysis and interpretations (Figure S22). The addition of small quantities of H_2_O (≤ 0.5 M H_2_O) elicits linear responses to β values in all cases, highlighting the systematic changes in *G*_*TA*_^‡, ε^ – *G*_*TE*_^‡, ε^ that occur due to hydrogen bonding interactions on ring-opening transition states. Over Al-BEA, β values decrease for MeOH, EtOH, and 1-BuOH and change negligibly for 1-HxOH (Figure 6a). Equivalent reactions over Zr-BEA yield β values which decrease for MeOH and EtOH, change negligibly for 1-BuOH, and increase for 1-HxOH (Figure 6b). Quantitative analysis of the trends in Figure 6 reveals the impact of hydrogen bonding interactions on β values depends on the ROH chain length (i.e., the slope changes; numerical discussion in Section S16). The ability for H_2_O to access the transition state depends on the ROH chain length. For example, MeOH and EtOH remain completely soluble in H_2_O while 1-BuOH and 1-HxOH are partially soluble and completely insoluble in H_2_O, respectively.^56,57^ Therefore, repulsion between larger ROH species and H_2_O may reduce the ability for H_2_O to hydrogen bond at the corresponding transition states in zeolite pores. Sterically hindered TA transition states may sense cosolvent repulsion to a greater extent than the more-accessible TE analogs, leading to the observed differences in β value trends. Regardless, differences in in the directions of β trends between ROH in Figure 6 imply the hydrogen bonding interactions of H_2_O must stem from the ROH component of transition states since interactions at the C_4_H_8_O* component should influence β trends in the same direction regardless of ROH. When fluid compositions do not contain H_2_O and instead contain only the ROH species (Figure 5), the size and hydrogen bonding ability of ROH likely inhibit large chain ROH from hydrogen bonding at transition states and leads to the invariant values of β observed, except in the case of methanolysis over Zr-BEA. We posit methanolysis regioselectivities over Al-BEA are less sensitive than those over Zr-BEA due to differences in the propensity of methanol molecules to discriminate between reactive intermediates (i.e., protonation may enhance interactions with both transition states). In summary, altering the stability of ROH molecules in ring-opening transition states *via* hydrogen bonding interactions allows for control of C_4_H_8_O ring-opening regioselectivities. Broadly, these findings additionally indicate the potential for tuning nucleophile and solvent hydrophobicities to elicit desired epoxide ring-opening product distributions.

## 4 Conclusions

Epoxide ring-opening with alcohol nucleophiles proceeds through an analogous set of elementary steps, regardless of zeolite acid site type (Brønsted or Lewis) or alcohol chain length (C_1_–C_6_ primary ROH). Reactant concentration rate dependencies suggest two kinetic regimes, whereby either a C_4_H_8_O- or ROH-derived species predominantly occupy active sites. Despite these mechanistic similarities, turnover rates typically increase 6 (Al-BEA) to 4 times (Zr-BEA) from MeOH to 1-HxOH due to differences in transition state stabilities (*G* ^‡^) and changes to bulk fluid properties (i.e., reactant activities). Apparent enthalpic barriers calculated from rates and ROH activities decrease linearly with increasing proton affinity, which directly shows the nucleophilicity of ROH species largely governs rate differences between ROH species. Yet, the molecular interpretation of apparent activation free energies and entropies gives evidence that the solvation of transition states by zeolite pore structures and solvent molecules also influence rates.

Ring-opening regioselectivities presented as product rate ratios (β) reflect differences in regioisomer transition state stabilities (i.e., *G*_*TA*_^‡, ε^ versus *G*_*TE*_^‡, ε^). Values of β respond directly to differences between the solvation of these transition states, which slightly enhance terminal alcohol product formation with increasing ROH chain length. However, regioselectivities for a given ROH remain largely invariant when changing the composition of the fluid due to the decreased hydrogen bonding ability and larger size of longer chain ROH species. The addition of H_2_O, with a lesser kinetic diameter, circumvents the challenges of hydrogen bonding among long chain ROH and provides an opportunity to influence regioselectivities by modifying the stability of ROH components of transition states. These findings agree with hydrogen bonding effects with homogeneous complexes,^4,5^ highlighting the potential to adapt molecular understanding from such systems (e.g., molecular orientations).

This work demonstrates how specific attributes of alcohol nucleophiles influence rates to ring-open epoxides at acid sites in microporous materials. The structure of the alkyl substituent to the alcohol affects total rates for epoxide consumption but also regioselectivities. Both the proton affinity and hydrogen bonding character of the nucleophile (and the propensity of the surrounding solvent to participate in hydrogen bonding) influence these outcomes, however, the span of these properties remain limited among aliphatic alcohols. These findings suggest that other classes of nucleophiles (e.g., amines, thiols, and substituted alcohols) may exhibit similar dependence on these attributes but offer wider ranges of properties, and in turn, greater rates or regioselectivities. Similarly, the topology of the microporous zeolite catalyst influences kinetics as the void dimensions influence the organization of the reactive species and solvating molecules. These factors offer a diverse set of parameters with potential to influence ring-opening catalysis.

## Supplementary Material



## Notes

X-ray diffraction; diffuse-reflectance UV–vis; ex-situ Raman spectroscopy; ^27^Al NMR spectroscopy; transmission FT-infrared spectroscopy; GC sensitivity factors; 1,2-diphenyl-1,2-ethylenediamine site titrations; hot filtrations; Madon–Boudart tests; supplemental kinetics; rate expression derivation; isothermal titration calorimetry and adsorption enthalpies; alcohol properties; reactant activity and excess energy; activation barriers; statistical analysis of uncertainties (PDF)
