# Supplementary material for: Epoxide Alcoholysis over M‑BEA Zeolites: Effects of Alcohol Chain Length on Rates and Regioselectivities
Source: ACS Catal. 2025 Oct 2;15(20):17280–91. doi: 10.1021/acscatal.5c04379 (PMC12538543; doi:10.1021/acscatal.5c04379)
Supplement: Supplementary file 1 [file cs5c04379_si_001.pdf]

Supporting Information

Epoxide Alcoholysis over M-BEA Zeolites: Effects of Alcohol Chain Length on Rates and Regioselectivities

Huston Locht[1], David S. Potts[2], Zahra Rangoonwala[1], David W. Flaherty[1,2*]

[1]School of Chemical and Biomolecular Engineering
Georgia Institute of Technology, Atlanta, Georgia, 30332 (USA)

[2]Department of Chemical and Biomolecular Engineering
University of Illinois Urbana-Champaign, Urbana, Illinois 61801 (USA)

*Corresponding author email: dflaherty3@gatech.edu

S1. Catalyst Characterization

S1.1 X-Ray Diffraction to Confirm Zeolite Crystallinity

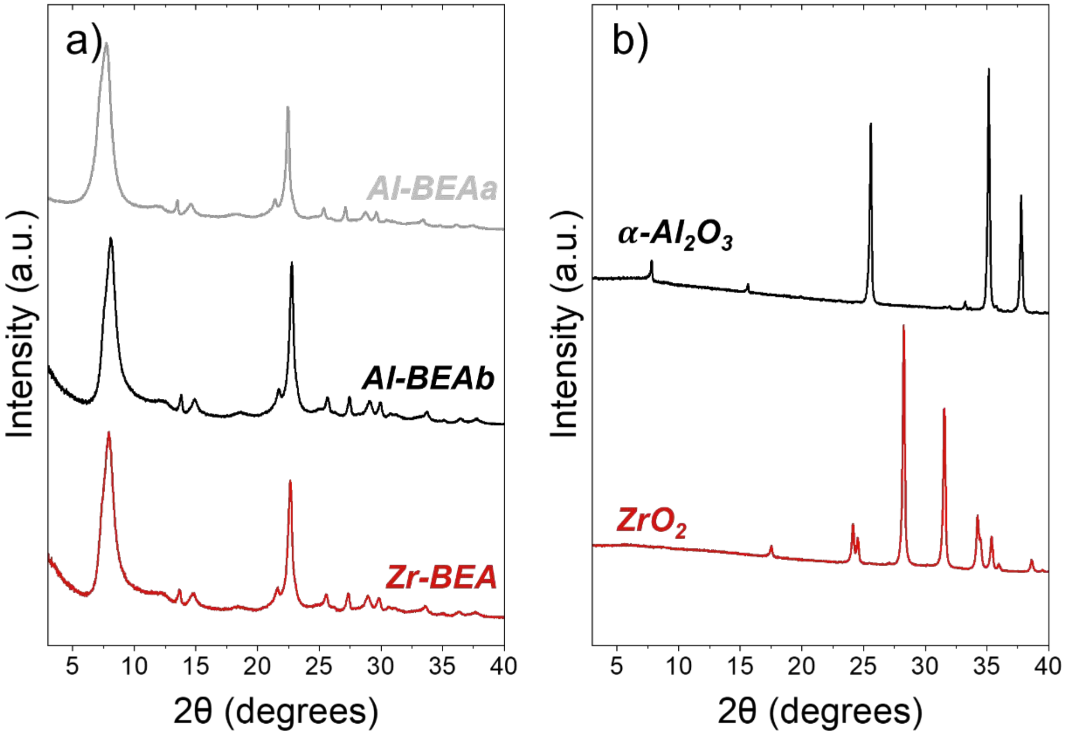

Figure S1. X-ray diffractograms for a) Al-BEAa (grey), Al-BEAb (black), and Zr-BEA (red) and b) corresponding bulk metal oxides (α-Al_2O_3 (black), ZrO_2 (red)). Spectra obtained with Cu Kα radiation (1.54 Å) under ambient conditions and vertically offset for clarity.

The crystallographic features of the zeolite materials in **Figure S1a** correspond to the features of the *BEA zeolite framework.[1] The presence of weak features above 25 degrees (25.5, 27.5, 29, and 30 degrees) are shown for all zeolite materials. **Figure S2b** shows at least one metal oxide feature (α-Al_2O_3, Sigma Aldrich, nanopowder 135 nm 99.9% trace metal basis; ZrO_2, Sigma Aldrich, powder 5 µm 99% trace metal basis) overlaps with each zeolite. However, the presence of features in all zeolite samples suggests that features are from the *BEA framework instead of metal oxide modalities. Overall, the diffractograms in **Figure S1** support the zeolites examines have no detectable metal oxide crystallites and the presence of crystalline *BEA framework.

S1.2 Diffuse Reflectance UV-Vis to Examine Metal Dispersity

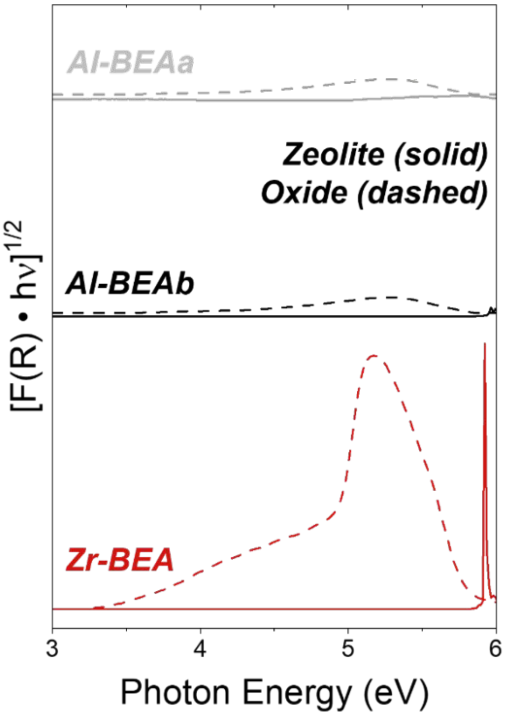

Figure S2. Tauc plots obtained with a UV-Vis spectrophotometer at ambient conditions for zeolites (solid lines; Al-BEAa (grey), Al-BEAb (black), Zr-BEA (red)) and corresponding metal oxides (dashed lines; α-Al_2O_3 (grey or black), ZrO_2 (red)). Tauc plots were normalized to the most intense features of UV-Vis spectra obtained from 200 to 800 nm and vertically offset for clarity.

The band gaps for each zeolite and the corresponding metal oxide were obtained by extrapolating the linear absorbance region in **Figure S2** to the baseline. The band gap is the intersection between the baseline and the linear region. Notably, reported band gaps of Al_2O_3 depend on the phase but are greater than 7 eV,[2, 3] consistent with the Al-BEA materials and α-Al_2O_3 showing negligible features within the photon energies examined. The band gap for Zr-BEA is calculated to be 5.9 eV and is greater than the band gap for ZrO_2 (4.9 eV), evidencing the Zr atoms are well dispersed in the *BEA framework.

The photon energy (E) in electron volts (eV) was obtained from wavelength (λ) using the following relation:

$$E = \frac{3 * 10^8 \left(\frac{m}{s}\right)}{\lambda\ (nm) * \frac{1}{10^9}\left(\frac{m}{nm}\right)} * h\ (J * s) * \left(6.242 * 10^{18}\left(\frac{eV}{J}\right)\right) \tag{S1}$$

where h is Planck's constant. The ordinate of **Figure S2** was obtained via Kubelka-Munk function ($F(R)$) of the raw percent reflectance ($\%R$) spectra:

$$F(R) = \frac{\left(1-\frac{\%R}{100}\right)^2}{2*\left(\frac{\%R}{100}\right)} \tag{S2}$$

The photon energy was then multiplied by $F(R)$ and the square root of the resulting quantity yielded the ordinate of **Figure S2**.

S1.3 *Ex situ* Raman Spectra to Confirm Metal Coordination

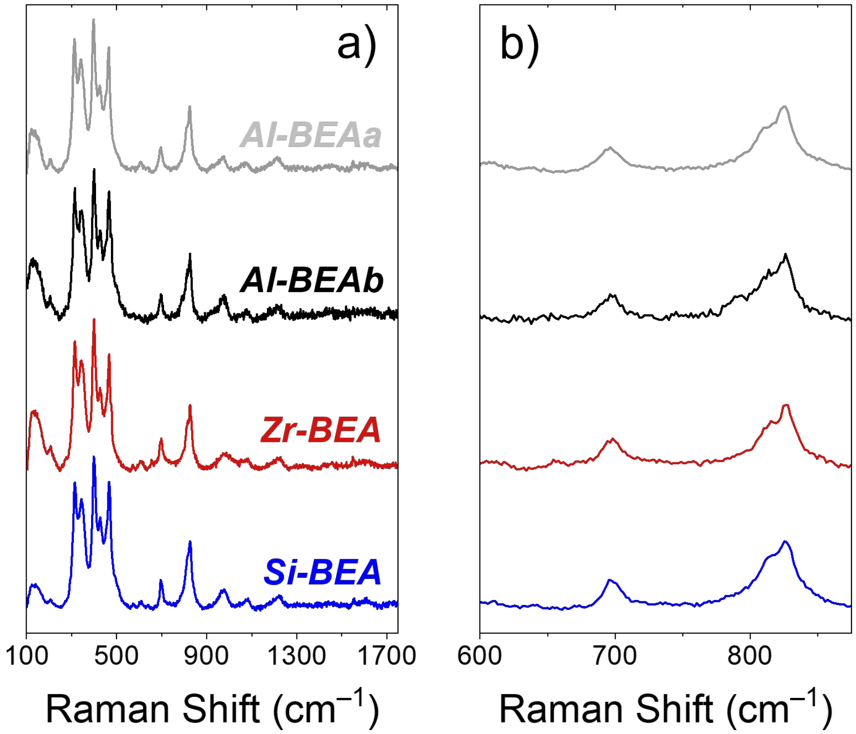

Figure S3. *Ex situ* Raman spectra of zeolites (Al-BEAa (grey), Al-BEAb (black), Zr-BEA (red), and Si-BEA (blue)) at a) the full range of Raman shifts examined and b) the range of 600-875 cm^{-1} for greater clarity. All spectra averaged 10 scans with an exposure time of 60 s by a 532 nm laser at ambient conditions. Spectra are normalized by maximum feature ~400 cm^{-1} and vertically offset for clarity.

Figure S3a shows all evaluated zeolites possess similar Raman spectra, regardless of active metal (or lack thereof in the case of Si-BEA). Intense peaks in the range of 250 to 550 cm^{-1} represent characteristic features of the *BEA framework: 315 and 345 cm^{-1} correspond to six-membered rings, 400 and 425 cm^{-1} correspond to five-membered rings, and 465 cm^{-1} represents four-membered rings.[4, 5] Some reports additionally attribute the peak at 695 cm^{-1} to the *BEA framework.[6, 7] The peak at 820 cm^{-1} corresponds to a symmetrical skeletal mode of microporous silicates.[8, 9] Finally, the peak at 144 cm^{-1} is present in both metal containing and Si-BEA, implying the feature corresponds to the *BEA framework or is an artifact from data collection.

Figure S3b provides greater magnification of the region in which metal-oxygen-metal features have been reported. All zeolites possess nearly identical spectra in the region and have no notable features corresponding to Al_2O_3 (640, 751 cm^{-1})[10-12] or ZrO_2 (610, 640 cm^{-1}).[13-15] Together, these data support the zeolite materials do not contain oligomeric metal oxide structures.

Raman spectra cannot directly confirm the presence of Al or Zr atoms in the zeolite framework (as reported for Ti-containing materials),[16-19] but the absence of metal oxide features indirectly suggests the metals reside at tetrahedral framework positions.

S1.4 ^{27}Al MAS NMR Spectra to Evaluate Al Coordination

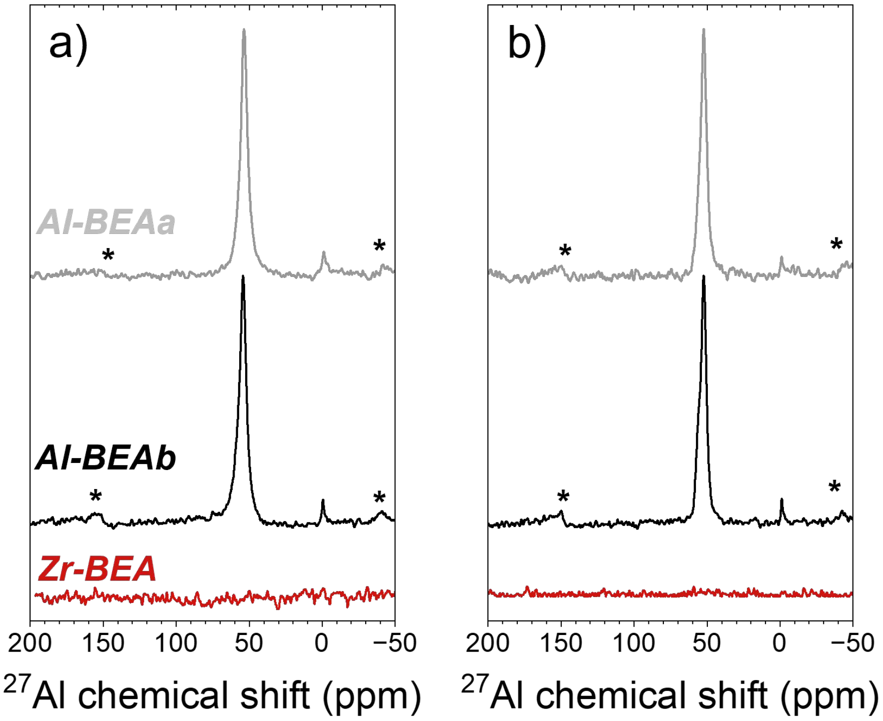

Figure S4. ^{27}Al MAS NMR spectra of a) untreated and b) hydrated zeolites (Al-BEAa (grey), Al-BEAb(black), Zr-BEA (red)). All spectra were collected at ambient conditions with a spin speed of 10 kHz. Asterisks represent spinning side-band features.

The coordination of Al in Al-BEA zeolites was evaluated in **Figure S4**. NMR spectral features centered ~50-60 ppm correspond to Al in tetrahedral coordination (commonly assumed to be framework-bound Brønsted acidic Al) whereas 0 ppm features correspond to octahedral coordination (commonly assumed to be extra-framework Lewis acidic Al).[20] The ambient conditions of the untreated zeolites (**Figure S4a**) include atmospheric H_2O that may bind in pairs to tetrahedral Al atoms and give rise to octahedral ^{27}Al NMR features.[20-22] The hydrated zeolites in **Figure S4b** similarly possess these features. In all cases, zeolites have measurable quantities of octahedral Al (6% (ambient) or 3% (hydrated) Al-BEAa; 3% (ambient and hydrated) Al-BEAb) that reflect similar quantities to active metal percentages estimated by site titration measurements (**Figure S13**).

No discernable ^{27}Al features exist for the Zr-BEA zeolite synthesized from a completely dealuminated parent Al-BEA-20 material, implying the complete removal of Al atoms from the framework prior to the incorporation of Zr atoms.

Overall, the ^{27}Al spectra in **Figure S4** support that Al-BEA materials contain minor fractions of octahedral Al and are unlikely to convolute kinetic measurements.

S1.5 Infrared Spectra of Zeolites to Determine Silanol Density

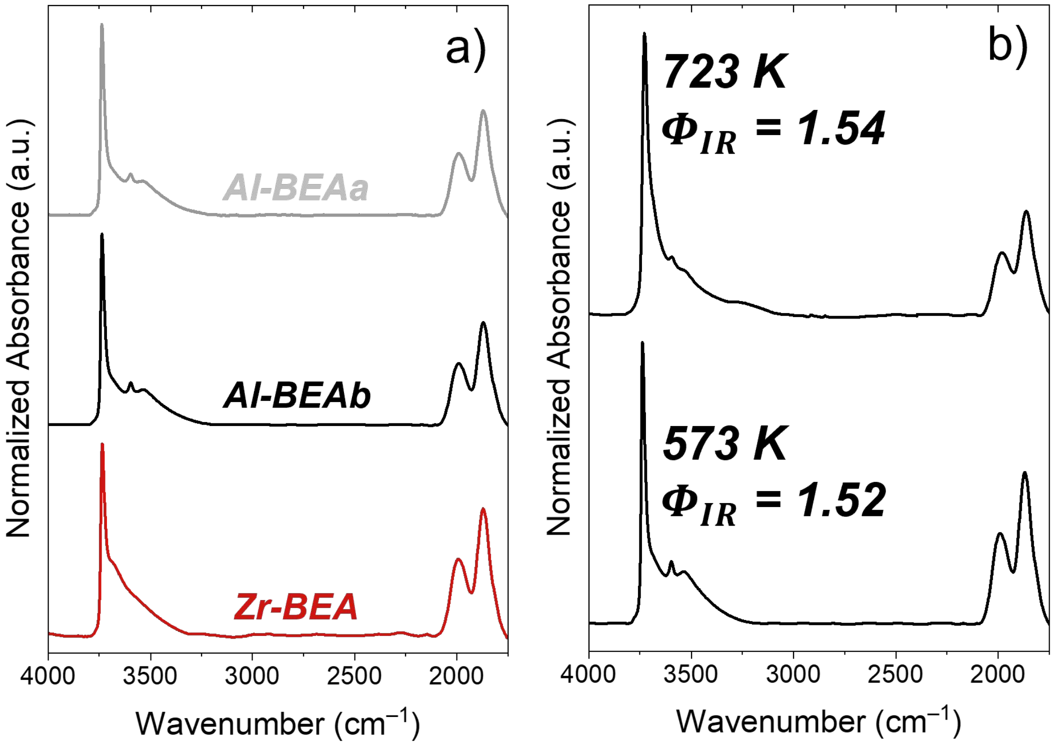

Figure S5. Infrared spectra of dehydrated zeolite samples (Al-BEAa (grey), Al-BEAb (black), Zr-BEA (red)). a) Spectra of samples dehydrated at 573 K. b) Spectra of Al-BEAb at 723 K and 573 K demonstrating near-complete dehydration was achieved. All spectra were collected under flowing Ar (101 kPa, 100 cm^3 min^{-1}) and are vertically offset for clarity.

All dehydrated zeolites in **Figure S5** possess similar features in infrared spectra. Similar ratios of ν(O-H) area (3300–3800 cm^{-1}) to ν(Si-O-Si) area (1800–2100 cm^{-1}) indicate all materials have similar densities of $(SiOH)_x$ groups associated with framework hydrophilicity.

To quantify the values of Φ_{IR} found in **Table 1** of the main text, peak fitting was performed on the spectra in **Figure S5** using Origin 2024 (OriginLab Corporation). Peak areas corresponding to the ν(O-H) and ν(Si-O-Si) region were found using Gaussian curves, an example of which is shown below in **Figure S6**.

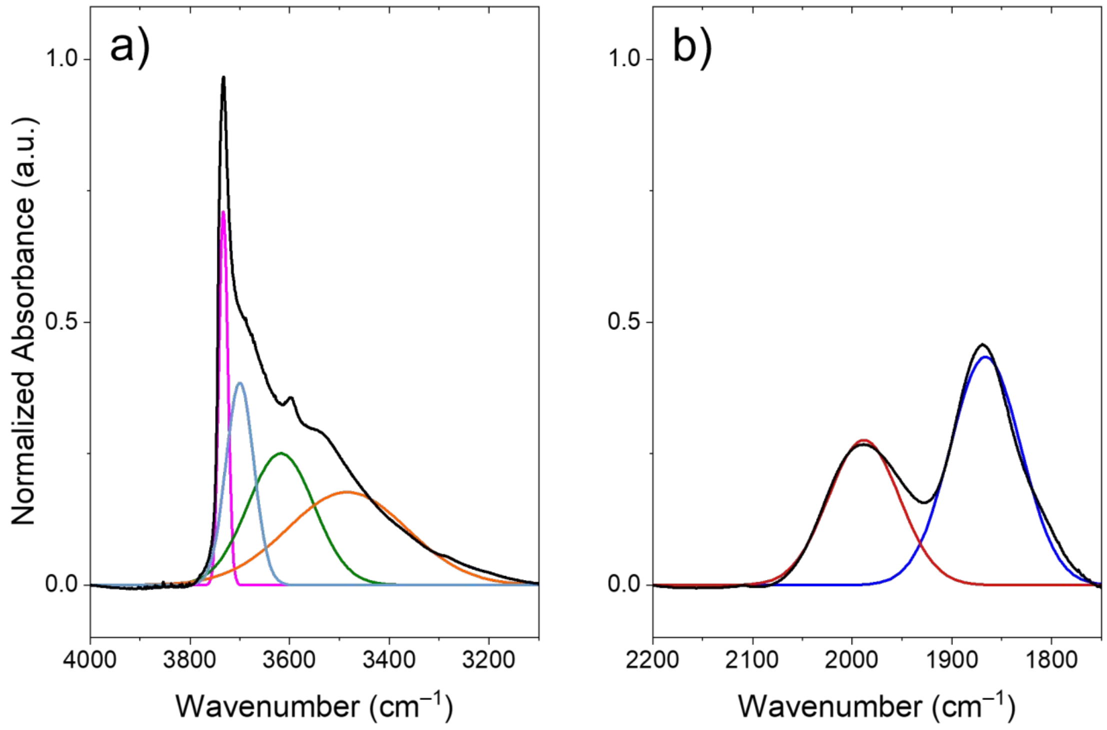

Figure S6. Peak fitting of a) the ν(O-H) region at ~3300-3800 cm^{-1} (pink, light blue, green, and orange) and b) the ν (Si-O-Si) region at ~1800-2100 cm^{-1} (red and blue) for Al-BEAb.

The equation to calculate Φ_{IR} is shown in Equation 1 in the main text and is:

$$\Phi_{IR} = \frac{A_{\nu(O-H)}}{A_{\nu(Si-O-Si)}} \quad \text{(S3)}$$

The pink peak (3733 cm^{-1}) in **Figure S6a** represents isolated SiOH which were excluded from Φ_{IR} calculations. The peak relating to Brønsted acidic protons (only observed in the Al-BEA samples ~3600 cm^{-1})[23, 24] was additionally excluded from Φ_{IR} calculations for Al-containing zeolite samples. The light blue (3700 cm^{-1}), green (3618 cm^{-1}), and orange peaks (3485 cm^{-1}) from **Figure S6a** represent the remaining O-H features. Light blue and orange peaks were summed to determine $A_{\nu(O-H)}$, since the green peak was assigned to the Brønsted proton. The red (1988 cm^{-1}) and blue peaks (1866 cm^{-1}) from **Figure S6b** represent Si-O-Si overtones and are summed to determine $A_{\nu(Si-O-Si)}$.

S1.6 Infrared Spectra of Pyridine in Zeolites to Elucidate Acid Site Character

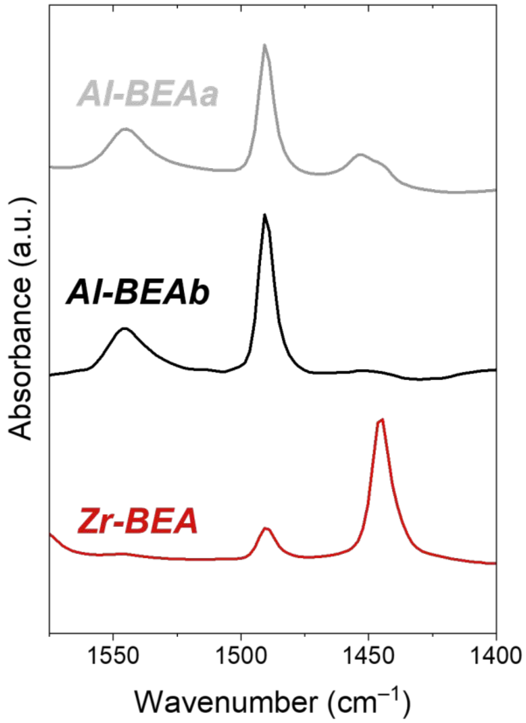

Figure S7. Infrared spectra of pyridine (C_5H_5N) bound to zeolites (Al-BEAa (black), Zr-BEA (red)) (1.4 kPa C_5H_5N, 101 kPa Ar, 393 K). Spectra are normalized to the largest feature and vertically offset for clarity.

Zeolite materials possess features corresponding to distinct binding modes of C_5H_5N in **Figure S7**. The feature shared by all materials at 1490 cm^{-1} corresponds to both Lewis and Brønsted acid sites.[25, 26] Only Al-BEAa and Al-BEAb possess features at 1550 cm^{-1} which corresponds to protonated C_5H_5N at Brønsted acid sites.[25-28] Finally, Al-BEAa and Zr-BEA possesses a feature at 1450 cm^{-1} which corresponds to Lewis acid bound C_5H_5N.[25, 27, 28]

Overall, the spectra of adsorbed C_5H_5N demonstrate the presence of Brønsted and Lewis active sites in the Al- and Zr-BEA materials, respectively. The Al-BEAb spectra does not possess features that correspond to Lewis acid-bound C_5H_5N and therefore the Al-BEAb material does not contain an observable concentration of Lewis acid sites. Similarly, the Zr-BEA spectra does not possess features that correspond to Brønsted acid-bound C_5H_5N and therefore the Zr-BEA material does not contain an observable concentration of Brønsted acid sites. However, the Al-BEAa material does possess features corresponding to both Brønsted and Lewis acid-bound C_5H_5N, suggesting this material may have a small quantity of Lewis acid character. Overall, the spectra of adsorbed C_5H_5N in **Figure S7** offers insight into the distribution of acid sites in the zeolite materials.

S2. Comparison of Rates Measured on Al-BEAa and Al-BEAb

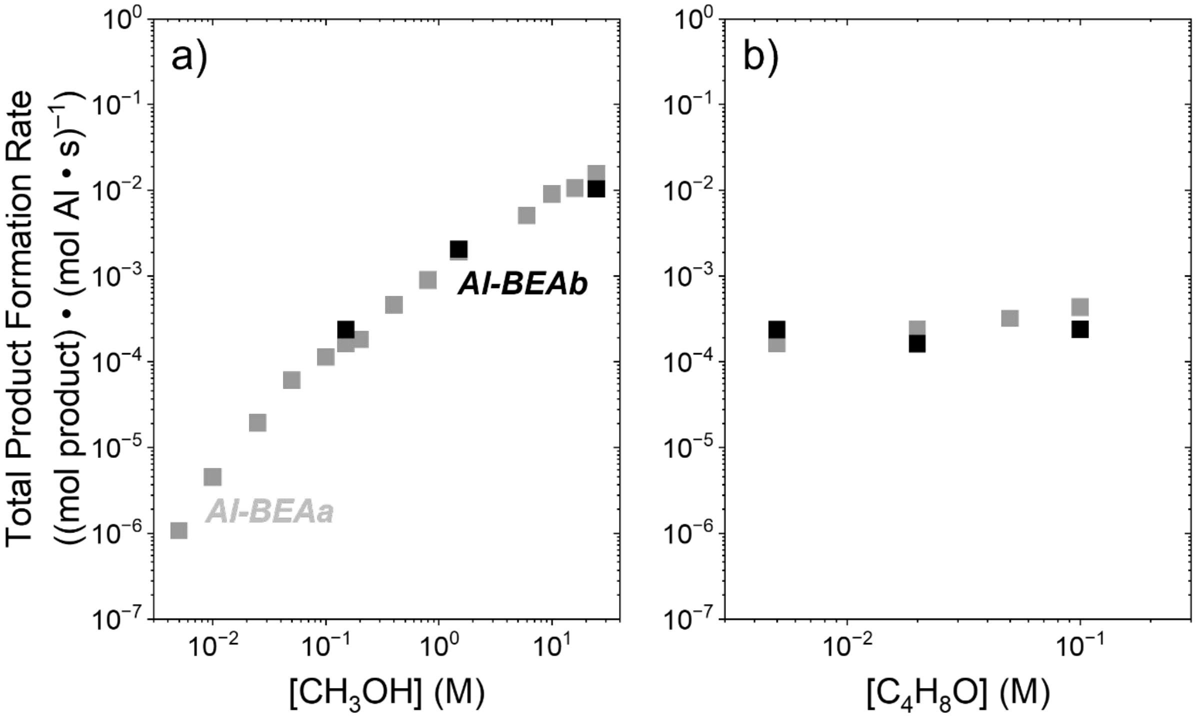

Figure S8. Total product turnover rates for C_4H_8O methanolysis as functions of a) methanol concentration (0.005 M C_4H_8O, CH_3CN solvent, 308 K) and b) epoxide concentration (0.15 M CH_3OH, CH_3CN solvent, 308 K) over Al-BEAa (grey) and Al-BEAb (black). Al-BEAa data adapted from previous work.[29]

Epoxide ring-opening rates are similar between both Al-BEAa and Al-BEAb across a range of reactant concentrations (**Figure S8**). Both materials show a roughly linear dependence in [CH_3OH] at lower concentrations and rates that remain constant with [C_4H_8O] in the regimes examined. The regioselectivities associated with the above reaction rates are shown in **Figure S9** below and are defined using **Equation 10** in the main text.

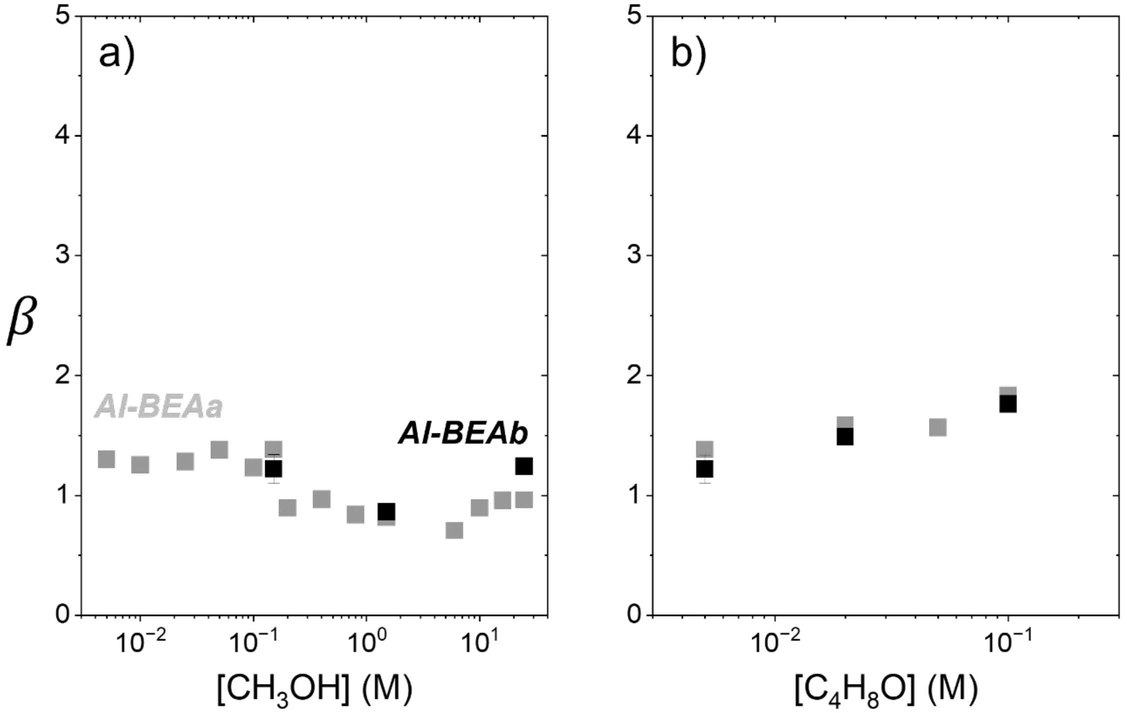

Figure S9. Regioselectivity (β) values for C_4H_8O methanolysis as functions of a) methanol concentration (0.005 M C_4H_8O, CH_3CN solvent, 308 K) and b) epoxide concentration (0.15 M CH_3OH, CH_3CN solvent, 308 K) over Al-BEAa (grey) and Al-BEAb (black). Al-BEAa data adapted from previous work.[29]

S3. Product Calibration Factors from Effective Carbon Number Method

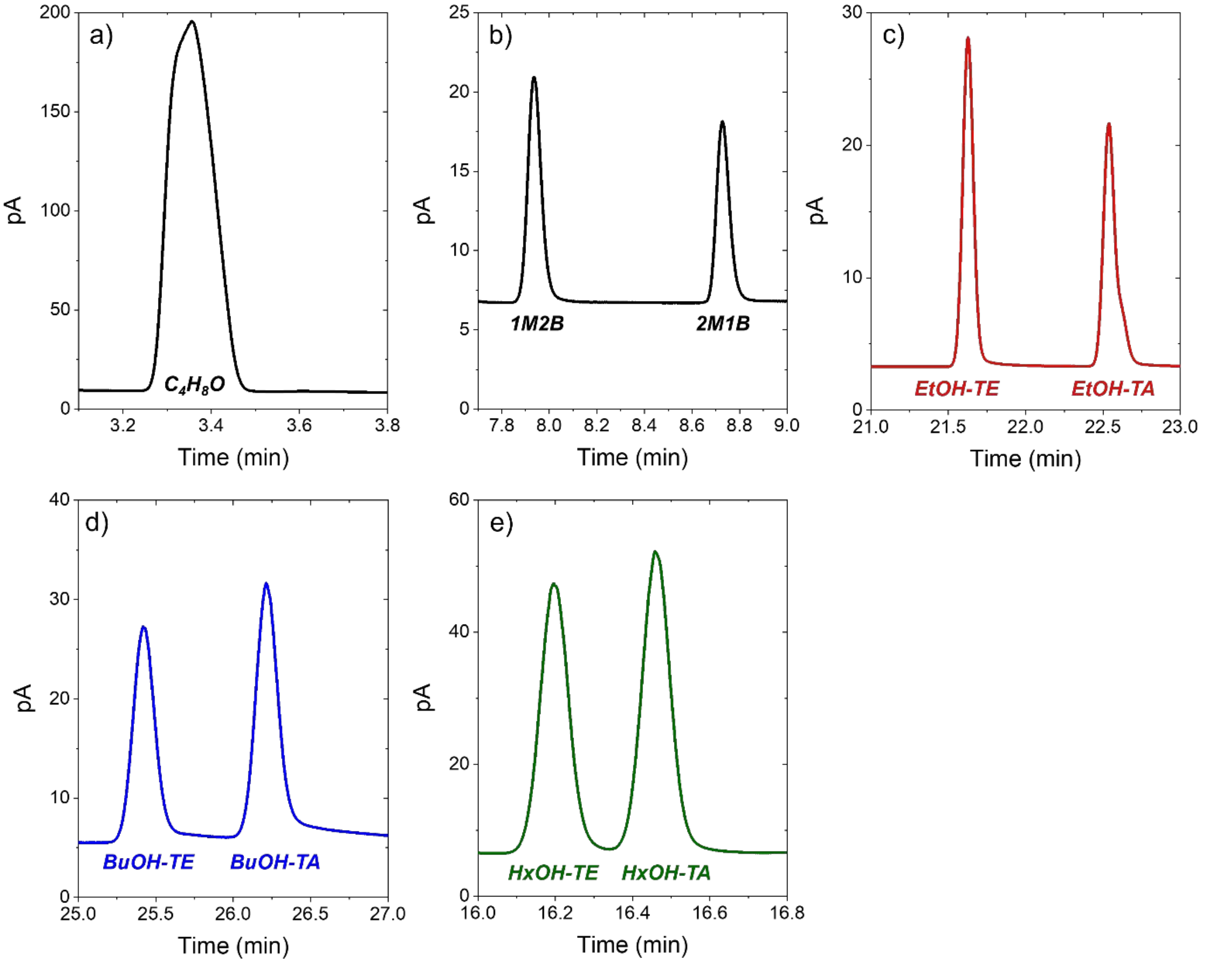

Figure S10. Raw GC chromatograms displaying peaks corresponding to a) C_4H_8O as well as b) methanolysis (1-methoxy-2-butanol (1M2B) and 2-methoxy-1-butanol (2M1B)), c) ethanolysis (1-ethoxy-2-butanol and 2-ethoxy-1-butanol), d) butanolysis (1-butoxy-2-butanol and 2-butoxy-1-butanol), and e) hexanolysis (1-hexoxy-2-butanol and 2-hexoxy-1-butanol) products formed via C_4H_8O ring-opening over Zr-BEA (a-b) or Al-BEAa (c-e) for 1 hour (0.005 M C_4H_8O, 6 M ROH, CH_3CN, 308 K). Zr-BEA data adapted from previous work.[29]

The GC chromatograms in **Figure S10** show the high signal-to-noise ratio and product peak separation achieved in the GC analysis. Separate GC methods were used for each alcohol which differ only in temperature profile. All other GC method parameters were identical, enabling the following sensitivity factor estimation (*vide infra*). We acknowledge the existence of two minor side products that elute at times similar to the MeOH and EtOH ring-opening products (~7.4 and 10.3 min) even in the absence of any alcohol in the system. While these peaks have not been identified, plausible side products like 1,2-butanediol and dibutyl ether have been ruled out in our previous contribution.[32] Furthermore, **Figure S11** demonstrates side product peak areas remain < 1% of the area of alcoholysis products and do not contribute to the analyses herein.

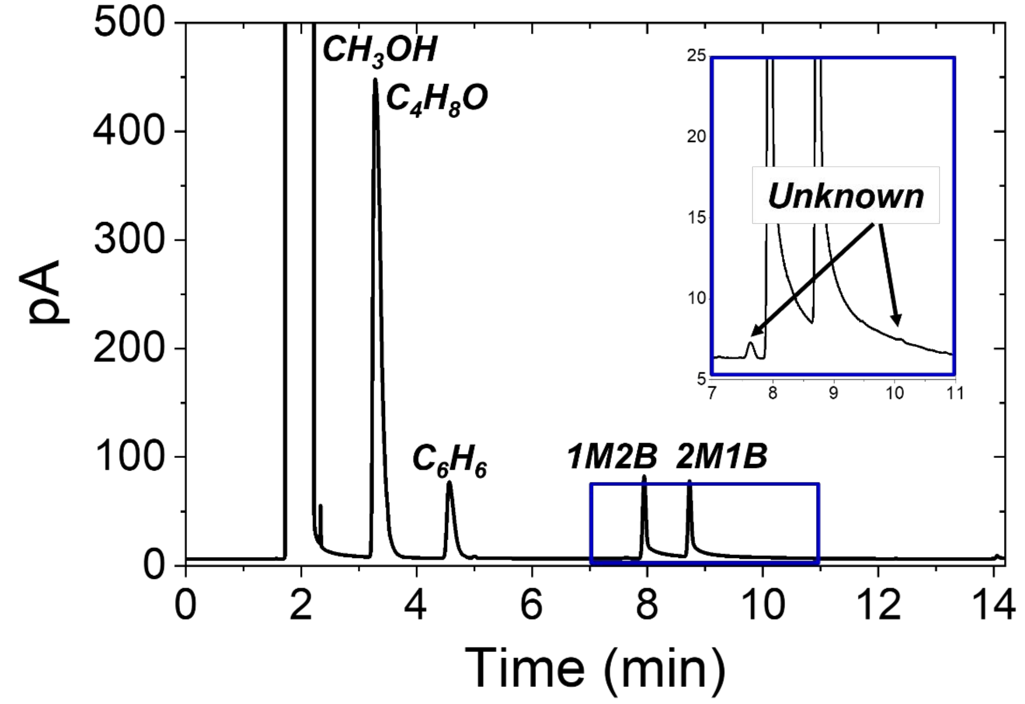

Figure S11. Raw GC chromatogram demonstrating minor side products (< 1% major product areas) that elute at times similar to methanolysis products (0.005 M C_4H_8O, neat M CH_3OH, 308 K).

Chromatograms for C_4H_8O and methanol ring-opening products (1-methoxy-2-butanol (1M2B) and 2-methoxy-1-butanol (2M1B)) were collected as functions of their concentration to determine GC sensitivity factors (SF) for the salable chemicals (**Figure S10**). The sensitivity factors are tabulated in **Table S1**.

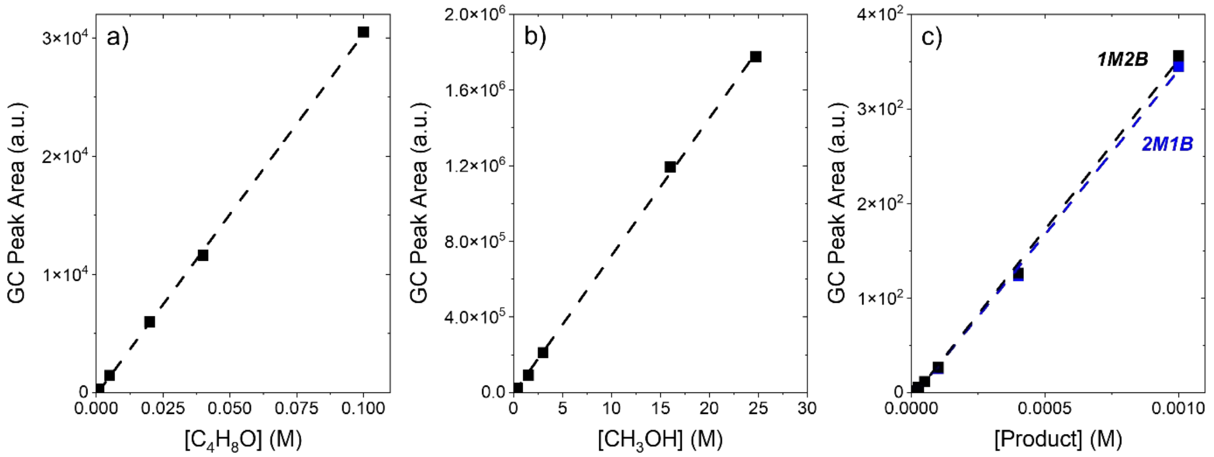

Figure S12. Integrated GC peak areas as functions of a) C_4H_8O concentration and b) 1M2B (black) and 2M1B (blue) concentrations in CH_3CN. Adapted from previous work.[29]

Ring-opening products for the C_2-C_6 alcohols cannot be readily purchased and instead were calculated using an effective carbon number (ECN) method.[30, 31] Based on this analysis, the ECN

of a species with one additional carbon would be one greater (e.g., ethanol is ~1 ECN greater than methanol).

To estimate the SF of higher chain length alcohol products, a relative weight response factor (F_{ij}) was first calculated for combinations of 1M2B or 2M1B and methanol.[30] For brevity, only 1M2B is shown.

$$F_{1M2B-CH_3OH} = \frac{SF_{CH_3OH} * MW_{1M2B}}{SF_{1M2B} * MW_{CH_3OH}} \quad \text{(S4)}$$

where SF_i and MW_i are the sensitivity factor and molecular weight of species i. From this value, an effective carbon number for each product is calculated:[30]

$$ECN_{1M2B} = \frac{MW_{1M2B} * ECN_{CH_3OH}}{MW_{CH_3OH} * F_{1M2B-CH_3OH}} \quad \text{(S5)}$$

where the ECN of methanol is 0.52.[30] The ECN values for all higher chain length alcohol products are therefore known by adding carbon equivalents to each product (e.g., adding 1 to ECN_{1M2B} to get $ECN_{1-ethoxy-2-butanol}$). With the ECN values of all products known, **Equations S5 and S4** are solved in reverse using methanol as the reference to yield $SF_{products}$ which have been summarized in **Table S1** below.

Table S1. Sensitivity factors for GC analyses.

Compound	Sensitivity Factor (peak area M^{-1})
CH_3OH	7.26×10^{4} [a]
C_4H_8O	3.03×10^{5} [a]
1M2B	3.49×10^{5} [a]
2M1B	3.39×10^{5} [a]
1-ethoxy-2-butanol	4.89×10^{5} [b]
2-ethoxy-1-butanol	4.78×10^{5} [b]
1-butoxy-2-butanol	7.68×10^{5} [b]
2-butoxy-1-butanol	7.57×10^{5} [b]
1-hexoxy-2-butanol	1.05×10^{6} [b]
2-hexoxy-1-butanol	1.04×10^{6} [b]

[a] Determined from calibration curves. [b] Determined from effective carbon number method.

S4. Site Counting with 1,2-Diphenylethylenediamine Titrations

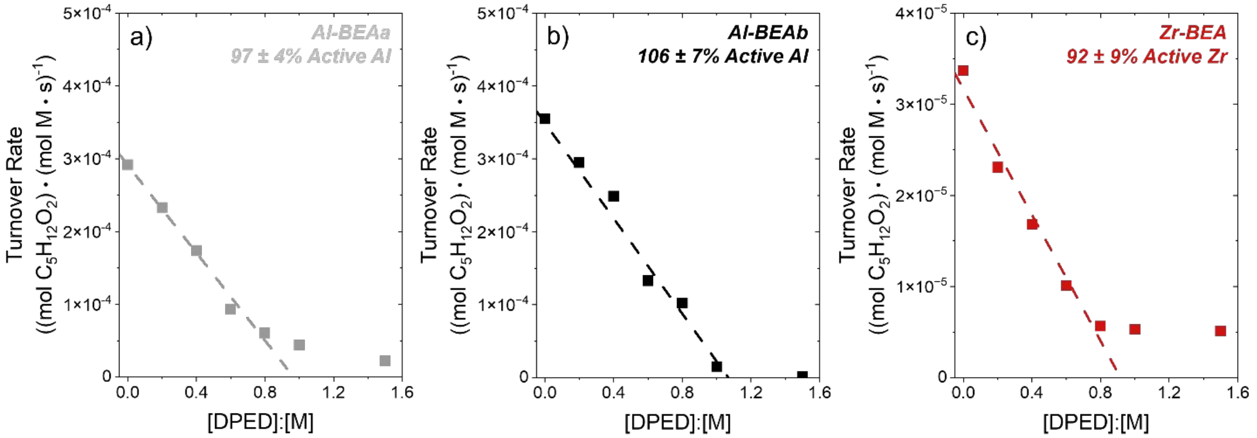

Figure S13. Total product turnover rates of C_4H_8O methanolysis over a) Al-BEAa, b) Al-BEAb, and c) Zr-BEA as a function of 1,2-diphenylethylenediamine (DPED) to active metal ratio (0.005 M C_4H_8O, 0.4 M CH_3OH, CH_3CN solvent, 308 K). Al-BEAa data adapted from previous work.[32]

The titration of active sites within zeolite catalysts has been used extensively to count acid sites in the materials and can be carried out with a variety of phosphonic acids or amines. The titrant 1,2-diphenylethylenediamine (DPED) was selected to poison active sites and was added in measured quantities related to active metal content to systematically poison active sites. Turnover rate inhibition by DPED was observed for low [DPED]:[M] for all catalysts. However, turnover rates remain measurable at [DPED]:[M] greater than unity, suggesting a small fraction of sites are unable to be titrated by DPED (e.g., due to proximity or other factors). Regardless, lines were fitted to the linear portion of curves at low [DPED]:[M] in **Figure S13** and extrapolated to the x-axis to estimate the number of active sites in each material. All materials are within error of 100% active metal atoms, validating their use in turnover rate calculations.

The change in DPED concentration ([DPED]) before and after reactions was tracked by GC FID to quantify the amount of active site poison in each reaction. A separate DPED adsorption experiment was conducted in metal-free Si-BEA to estimate the portion of DPED bound to framework moieties (e.g., silanol groups or Si-O-Si). The quantity of DPED in the Si-BEA test was equivalent to the largest volume in **Figure S13**. The mass of Si-BEA was similar to the mass of Al- and Zr-BEA measurements in **Figure S13**. In all cases (including the Si-BEA test), the [DPED] decreases by > 95% after 1 h, suggesting that DPED preferentially enters the zeolite to bind regardless of metal presence. The similar uptake in the Si-BEA material suggests DPED that does not bind to acid sites may bind to framework moieties. Nevertheless, the mechanism for the systematic, and stoichiometric, titration of Al and Zr active sites by DPED is most simply described by DPED preferentially binding to active metal when present, suppressing C_4H_8O ring-opening, and validating the quantity of active metal does not contribute to rate differences. We do not exclude the possibility that DPED may bind elsewhere in the absence of Al and Zr.

S5. Hot Filtration to Verify Metal Remains in Framework

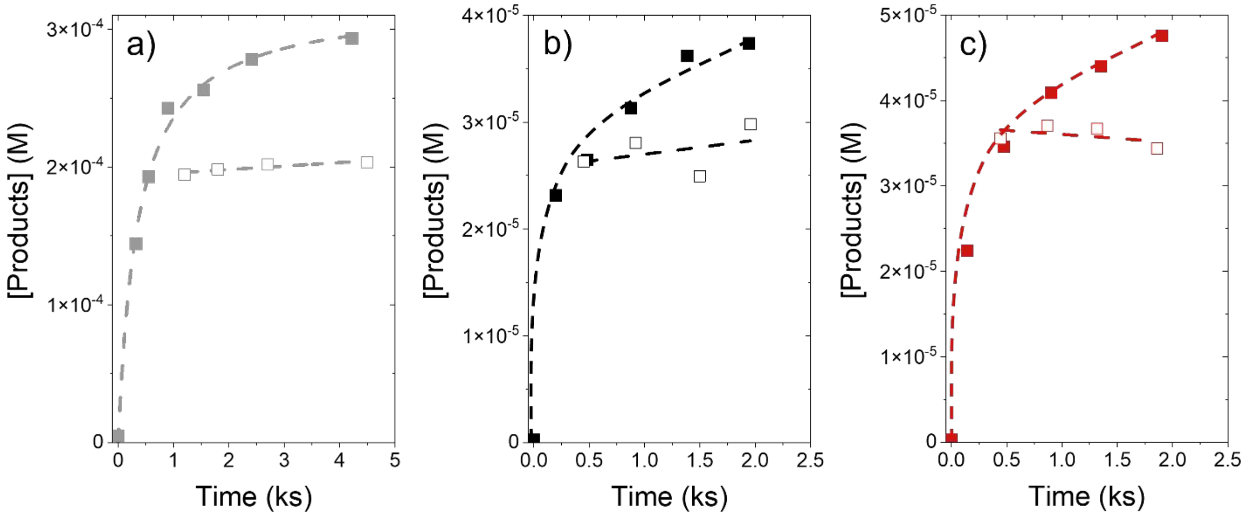

Figure S14. Total product concentrations as functions of time (0.005 M C_4H_8O, 6 M CH_3OH, CH_3CN solvent, 308 K) over a) Al-BEAa (grey), b) Al-BEAb (black), and c) Zr-BEA (red). Catalyst containing samples (filled points) and aliquots separated from catalyst after some time (hollow points) are shown. Al-BEAa data adapted from previous work.[32]

Figure S14 shows product formation ceases after hot filtration for all zeolites evaluated. These data therefore evidence that metal atoms do not leach out of the zeolite framework and into the solvent during ring-opening reactions. Furthermore, GC analyses are conducted on reaction solutions after large amounts of time waiting on other samples in queue (regularly over 24 hours). If there were leachable sites, GC peak areas would likely not follow the typical batch reaction concentration curvature shown in **Figure S14** due to continuing to convert epoxide between filtration and GC analysis.

S6. Madon-Boudart Criterion Tests to Verify Freedom from Mass Transport Limitations

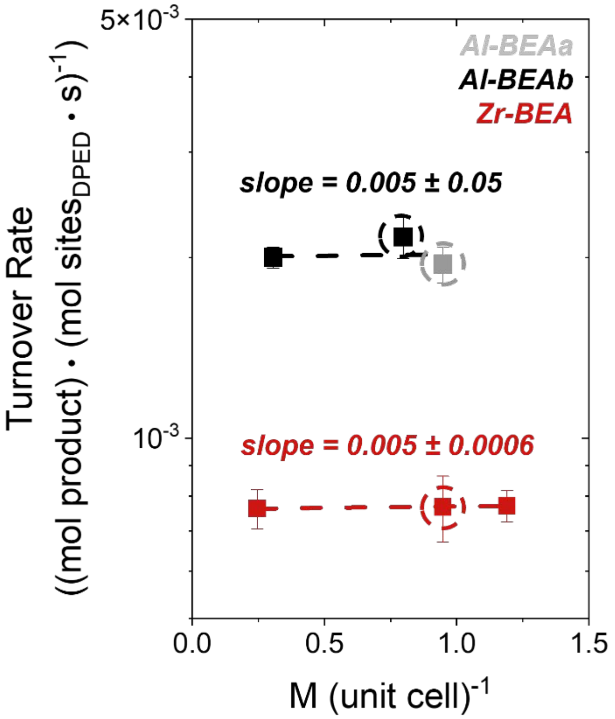

Figure S15. Total product turnover rates for C_4H_8O alcoholysis as a function of active metal per unit cell over Al-BEA (grey, black) and Zr-BEA (red) (0.005 M C_4H_8O, 0.4 1-HxOH, CH_3CN solvent, 308 K). Circled points signify zeolites used for study. Error bars represent error from turnover rate measurements and active metal estimated by DPED site titrations. For samples not used in this study, 100 ± 4% active metal was assumed in agreement with the minimum error from **Figure S13**.

Turnover rates change negligibly as a function of active metal loading (**Figure S15**), validating the Madon-Boudart criterion and ruling out contributions from internal mass transport artifacts on kinetic analyses (which would yield a negative slope).

We acknowledge slope fitting in **Figure S15** suggests turnover rates over Zr-BEA may increase slightly (0.005 ± 0.0006 mol product • $n_{unit\ cell}$ • mol $sites_{DPED}^{-1}$ • s^{-1} • n_{metal}^{-1}) as a function of increased Zr loading. The observed behavior for Zr-BEA samples may stem from error in estimating active site percentages (estimated for unused catalysts to be 100 ± 4%, in line with the smallest error in **Figure S13**) or site-site interactions which become apparent above ~1 metal atom per unit cell (e.g., due to overlapping solvent structures). However, we presume site-site interactions in the Zr-BEA sample used in this study would be minor due to Zr-BEA containing < 1 Zr per unit cell.

S7. Individual Product Formation Rates for C_4H_8O Alcoholysis

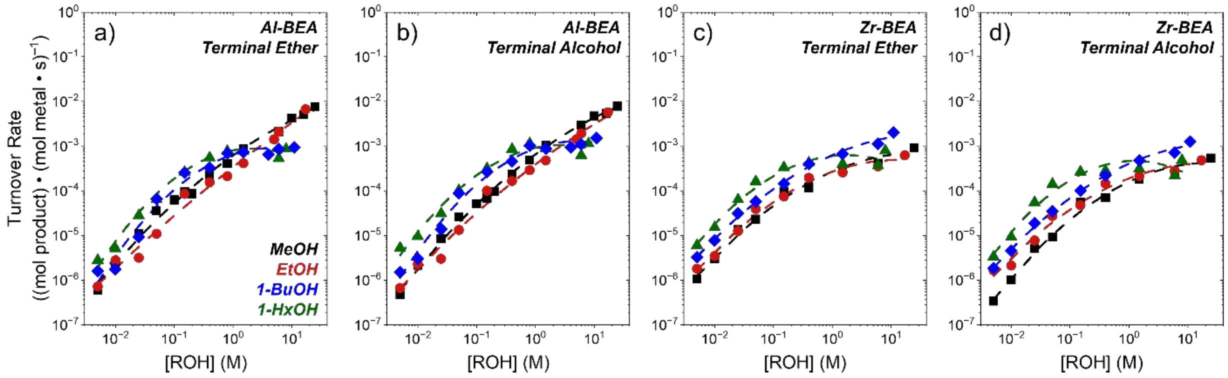

Figure S16. Turnover rates of a) Al-BEA terminal ether products, b) Al-BEA terminal alcohol products, c) Zr-BEA terminal ether products, and d) Zr-BEA terminal alcohol products for C_4H_8O ring opening as functions of methanol (black), ethanol (red), 1-butanol (blue), and 1-hexanol (green) concentration (0.005 M C_4H_8O, CH_3CN solvent, 308 K). Data for methanol over Al-BEA adapted from previous work.[29]

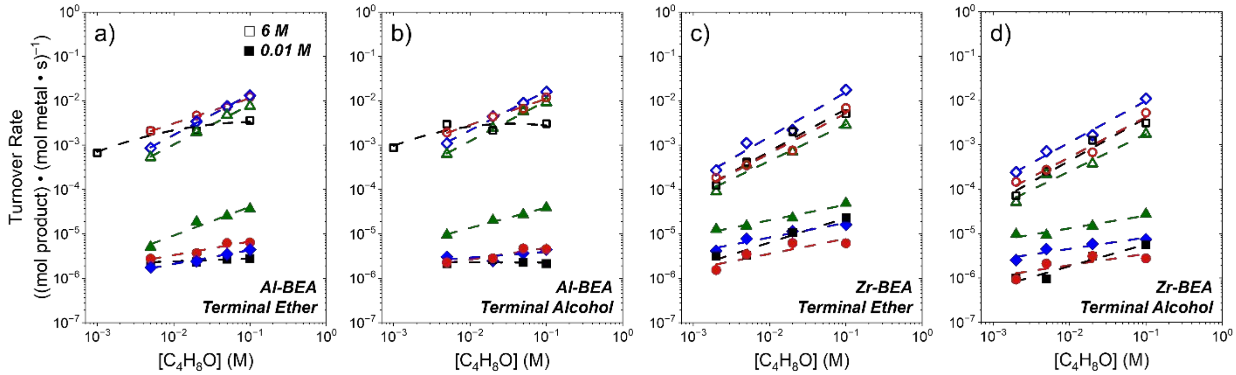

Figure S17. Turnover rates of a) Al-BEA terminal ether products, b) Al-BEA terminal alcohol products, c) Zr-BEA terminal ether products, and d) Zr-BEA terminal alcohol products for C_4H_8O ring opening with methanol (black), ethanol (red), 1-butanol (blue), and 1-hexanol (green) as functions of C_4H_8O concentration (0.01 (solid) or 6 M (hollow) ROH, CH_3CN solvent, 308 K). Data for methanol over Al-BEA adapted from previous work.[29]

Figures S16 and S17 show the individual product formation rates for C_4H_8O alcoholysis over Al- and Zr-BEA catalysts. Total product formation rates shown in **Figures 1 and 2** in the main text. In all cases, nearly identical turnover rate dependencies on reactant concentrations are observed. Together, these data provide evidence both products form through analogous pathways involving similar sets of intermediates.

S8. Turnover Rate Expression Derivation for C_4H_8O Alcoholysis

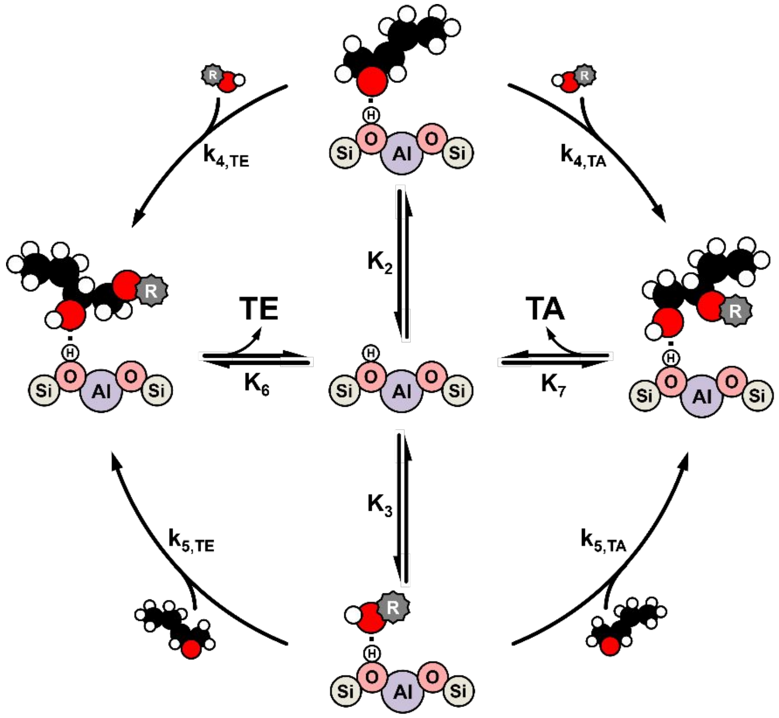

Scheme S1. Proposed reaction mechanism for C_4H_8O ring-opening with alcohols over Brønsted acidic Al-BEA catalysts. For clarity, we omit reversible adsorption of CH3CN (step 1), spectating solvent molecules, and the possibility of adsorbed species to bind in dissociative configurations—though these binding modes may be possible. All adsorption steps are assumed to be reversible (denoted by a double arrow), while a single arrow represents an irreversible step.

Scheme S1 and **Scheme 2** in the main text visualize a proposed mechanism for C_4H_8O alcoholysis over Brønsted and Lewis acid zeolites. Both mechanisms include a bare zeolite surface that may reversibly adsorb CH_3CN (step 1), C_4H_8O (step 2), or ROH (step 3). The complementary reagent reacts with the surface-bound species (ROH for C_4H_8O*, C_4H_8O for ROH*; * denotes surface species) in a kinetically relevant step (steps 4 and 5, respectively) to form either regioisomer. The terminal ether (TE) and terminal alcohol (TA) products then must desorb (steps 6 and 7) to return the bare active site and complete the catalytic cycle. Epoxide ring-opening rates for the sum of both pathways equal **Equation 2** from the main text:

$$r_{RO} = r_4 + r_5 = k_4[C_4H_8O^*][ROH] + k_5[C_4H_8O][ROH^*] \tag{S6}$$

where r_4, r_5, k_4, and k_5 are the sum of the rates and rate constants to form product from both pathways (i.e., $k_4 = k_{4,TE} + k_{4,TA}$) and $[C_4H_8O^*]$ and $[ROH^*]$ are the concentrations of surface bound intermediates from each reactant. Applying the pseudo-steady state hypothesis to the bound intermediates yields the following form which only depends on measurable concentrations:

$$r_{RO} = r_4 + r_5 = k_4[C_4H_8O^*][ROH] + k_5[C_4H_8O][ROH^*] \tag{S7}$$

A site balance $[L]$ can be written for the system of possible surface species:

$$[L] = [\,*\,] + [CH_3CN^*] + [C_4H_8O^*] + [ROH^*] + [TE^*] + [TA^*] \tag{S8}$$

where [$*$] denotes the concentration of empty sites and other terms are bound solvent, reactant, and product species. Substituting expressions for each of the terms in **Equation S8** yields:

$$\frac{[L]}{[*]} = 1 + K_1[CH_3CN] + \frac{k_2[C_4H_8O]}{k_{-2}+k_4[ROH]} + \frac{k_3[ROH]}{k_{-3}+k_5[C_4H_8O]} + \frac{k_4[ROH]+k_5[C_4H_8O]+k_{-6}[TE]}{k_6} + \frac{k_4[ROH]+k_5[C_4H_8O]+k_{-7}[TA]}{k_7} \quad \text{(S9)}$$

Substituting **Equation S9** into **Equation S7** yields the full rate expression (simplified into **Equation 3** in the main text):

$$\frac{r_{RO}}{[L]} = \frac{\frac{k_2k_4[C_4H_8O][ROH]}{k_{-2}+k_4[ROH]} + \frac{k_3k_5[C_4H_8O][ROH]}{k_{-3}+k_5[C_4H_8O]}}{1+K_1[CH_3CN]+\frac{k_2[C_4H_8O]}{k_{-2}+k_4[ROH]}+\frac{k_3[ROH]}{k_{-3}+k_5[C_4H_8O]}+\frac{k_4[ROH]+k_5[C_4H_8O]+k_{-6}[TE]}{k_6}+\frac{k_4[ROH]+k_5[C_4H_8O]+k_{-7}[TA]}{k_7}} \quad \text{(S10)}$$

At sufficiently low excess of alcohol, rates are 1st order in ROH and 0th order in C_4H_8O, suggesting sites covered by C_4H_8O* species. In this case, the 3rd term in the denominator (corresponding to [$C_4H_8O^*$]) dominates and **Equation S10** can be simplified:

$$\frac{r_{RO}}{[L]} = \frac{k_2k_4[ROH](k_{-3}+k_5[C_4H_8O])+k_3k_5[ROH](k_{-2}+k_4[ROH])}{k_2(k_{-3}+k_5[C_4H_8O])} \quad \text{(S11)}$$

Equation S11 matches the kinetics in **Figures 1 and 2** of the main text only when the first term in the numerator dominates such that:

$$\frac{r_{RO}}{[L]} = k_4[ROH] \quad \text{(S12)}$$

The simplification to yield **Equation S12** requires the value of $k_2k_4 \gg k_3k_5$. C_4H_8O adsorbs to Al- and Zr-BEA ~10-20 times more exothermically than ROH species (*vide infra*, **Figure S18**), implying that the equilibrium constant $K_2 \gg K_3$ and thus $k_2 \gg k_3$. The strong binding strength of C_4H_8O is therefore assumed to cause the first term in **Equation S11** to dominate and lead to the similar rate trends observed at low [ROH].

At sufficient excesses of alcohol, rates tend toward 0th order in ROH and 1st order in C_4H_8O, suggesting sites covered by ROH* species. In this case, the 4th term in the denominator (corresponding to $[ROH^*]$) dominates and **Equation S10** can be simplified differently to:

$$\frac{r_{RO}}{[L]} = \frac{k_2k_4[C_4H_8O](k_{-3}+k_5[C_4H_8O])+k_3k_5[C_4H_8O](k_{-2}+k_4[ROH])}{k_3(k_{-2}+k_4[ROH])} \quad \text{(S13)}$$

Equation S13 matches the kinetics in **Figures 1 and 2** of the main text only when the second term in the numerator dominates such that:

$$\frac{r_{RO}}{[L]} = k_5[C_4H_8O] \quad \text{(S14)}$$

At sufficiently high stoichiometric excesses of ROH compared to C_4H_8O, the [ROH] term likely dominates over all other terms in the numerator and leads to the second term dominating and leads to **Equation S14**.

Equations S12 and S14 account for all kinetic regimes observed at the concentrations evaluated in **Figures 1 and 2** in the main text. To further validate the use of the proposed mechanism for all

combinations of ROH and zeolite, **Figures S18 and S19** below present the results of simultaneously fitting the data in **Figures 1 and 2** to **Equation 3** in the main text. **Figure S18** additionally includes the root mean log-squared error (RMSE) for each fit.

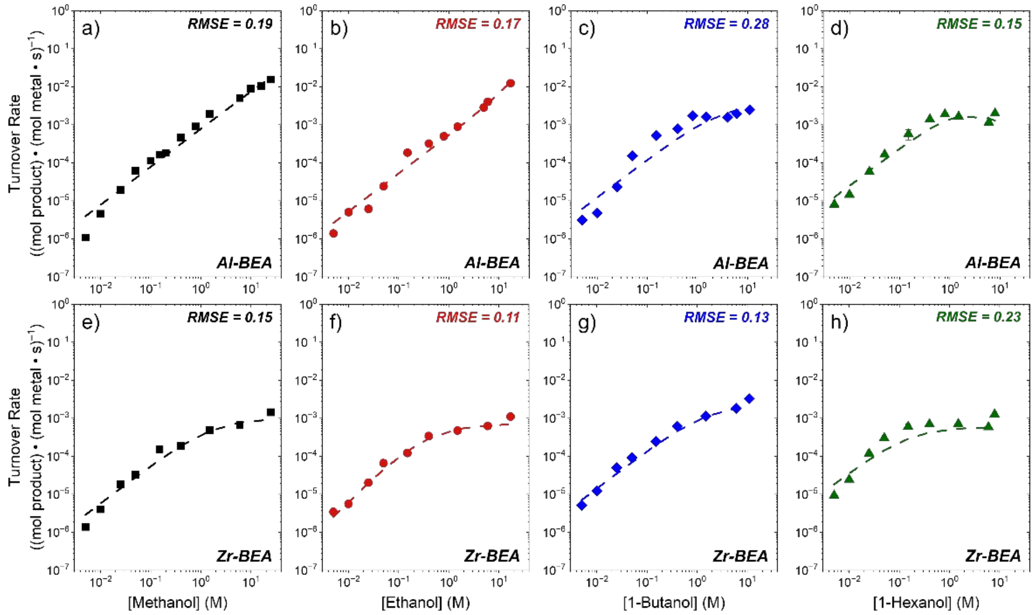

Figure S18. Turnover rates for C_4H_8O alcoholysis as functions of methanol (MeOH, black square), ethanol (EtOH, red circle), 1-butanol (1-BuOH, blue diamond), and 1-hexanol (1-HxOH, green triangle) concentrations over (a-d) Al-BEA and (e-h) Zr-BEA (0.005 M C_4H_8O, CH_3CN solvent, 308 K). Dashed lines represent global fits of the combined data from Figures 1 and 2 to Equation 3. Methanol Al-BEA adapted from previous work.[29]

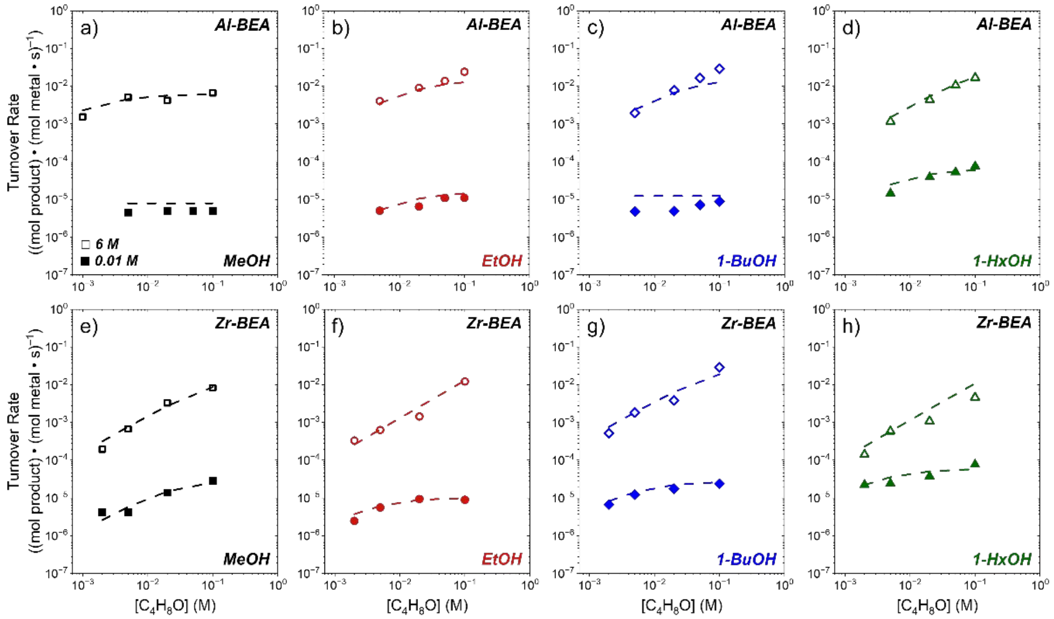

Figure S19. Turnover rates for C_4H_8O alcoholysis as functions of C_4H_8O concentrations at 0.01 M (filled) and 6 M (hollow) methanol (MeOH, black square), ethanol (EtOH, red circle), 1-butanol (1-BuOH, blue diamond), and 1-hexanol (1-HxOH, green triangle) over (a-d) Al-BEA and (e-h) Zr-BEA (CH_3CN solvent, 308 K). Dashed lines

represent global fits of the combined data from Figures 1 and 2 to Equation 3. Methanol Al-BEA adapted from previous work.[29]

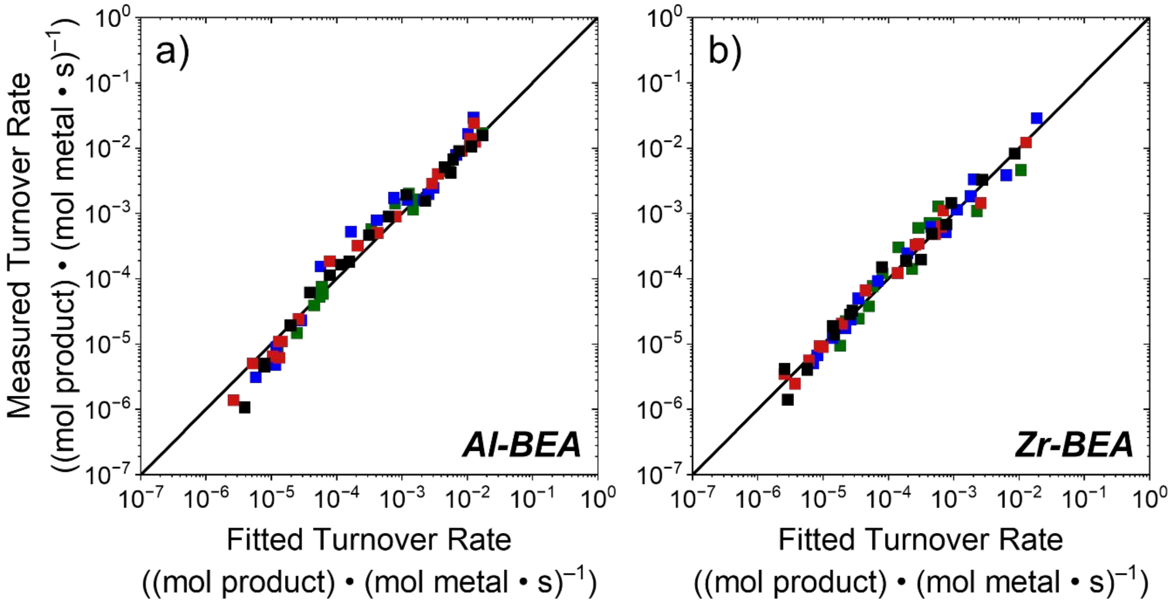

Figure S20. Parity plots of measured and fitted turnover rates for C_4H_8O ring-opening with methanol (MeOH, black), ethanol (EtOH, red), 1-butanol (1-BuOH, blue), and 1-hexanol (1-HxOH, green) over a) Al-BEA and b) Zr-BEA (0.002-0.1 M C_4H_8O, 0.005-neat M ROH, CH_3CN solvent, 308 K).

S9. Adsorption Enthalpies of Reactants onto Zeolites in CH_3CN Solvent

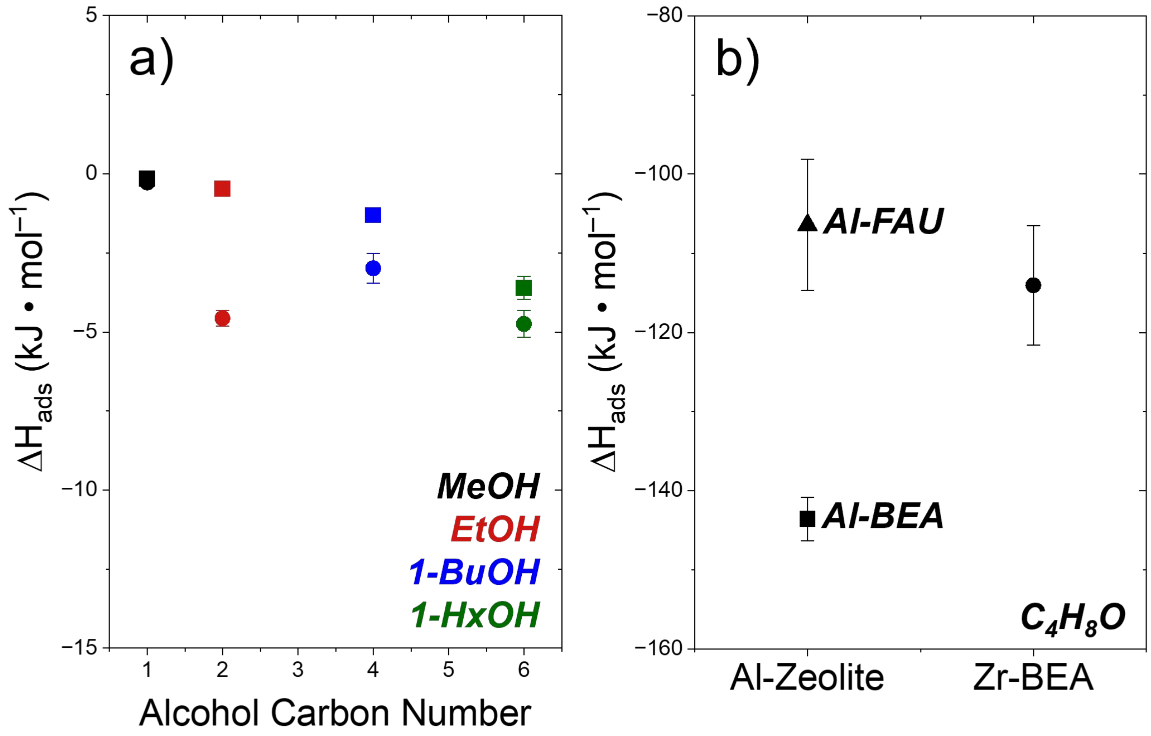

Figure S21. Adsorption enthalpies of a) ROH (methanol (black), ethanol (red), 1-butanol (blue), 1-hexanol (green)) and b) C_4H_8O over Al-BEA (square), Al-FAU (triangle, Thermo Scientific Si/Al = 120), and Zr-BEA (circle) (0.005 M ROH or C_4H_8O, CH_3CN solvent, 308 K).

Isothermal titration calorimetry was used to obtain the adsorption enthalpies shown in **Figure S21** (see **Section S18** for thermograms). In both Al- and Zr-BEA, C_4H_8O adsorbs significantly stronger (by 140 and 109 kJ mol^{-1}) than the most exothermic alcohol (1-hexanol). These data provide evidence to why ROH does not saturate active sites until in significant stoichiometric excess. Increasing the alcohol chain length typically increases the exothermicity of adsorption, suggesting 1-hexanol can compete more effectively than methanol for active sites. The increase in alcohol exothermicity with increased chain length also supports why sites are saturated by 1-hexanol at lower excesses than methanol. Finally, the increase in exothermicity with decreasing zeolite pore size supports the expected trend in the case of enthalpic barriers depending on spatial confinement in the zeolite pores when the ROH chain length increases (**Section S14**).

S10. Properties of Primary Alcohols

Table S2. Properties of primary alcohols.

Alcohol	Proton Affinity (kJ mol^{-1})	Formation Energy (kJ mol^{-1})	Kamlet-Taft Hydrogen Bond Donor Parameter[33]	Critical Volume (cm^3 mol^{-1})[34]
methanol	754[35]	-240[36]	0.98	117
ethanol	776[35]	-277[36]	0.86	168
1-butanol	789[35]	-327[37]	0.84	274
1-hexanol	799[38]	-378[37]	0.80	387

S11. Preliminary C_4H_8O Hydrolysis Rates Mimic Alcoholysis

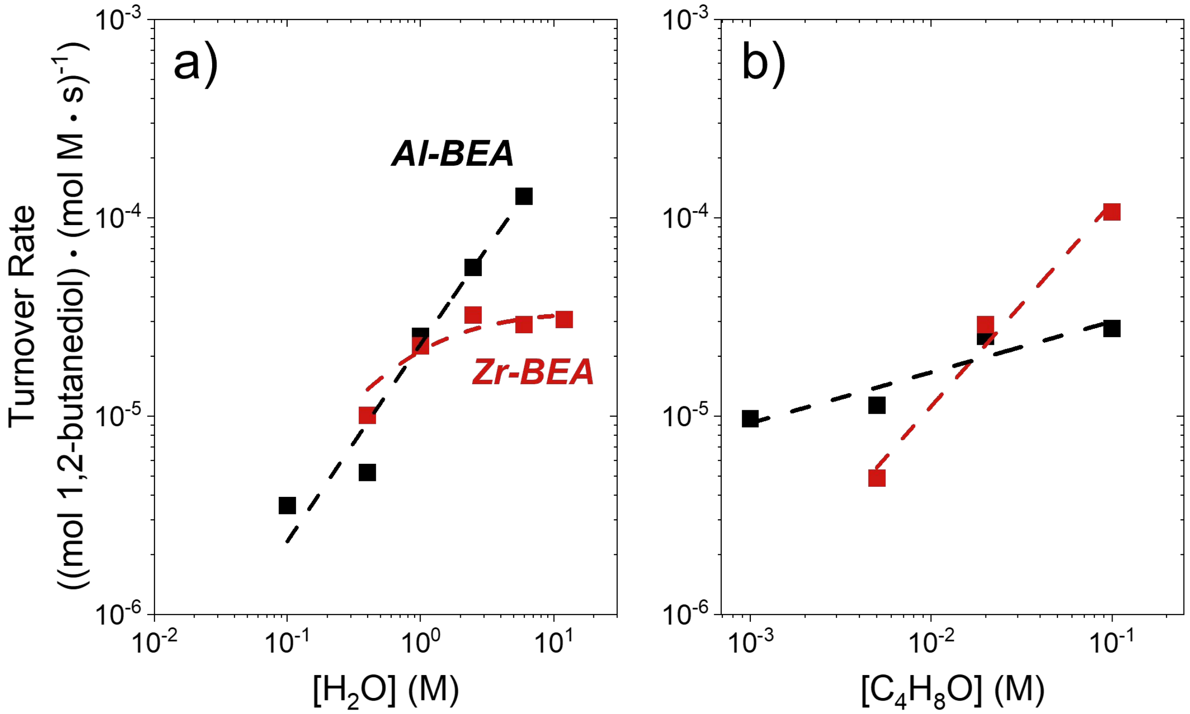

Figure S22. Turnover rates for C_4H_8O hydrolysis over Al-BEA (black) and Zr-BEA (red) as functions of a) H_2O concentration (0.02 M C_4H_8O, CH_3CN solvent, 308 K) and b) C_4H_8O concentration (1 M (Al-BEA) or 3 M (Zr-BEA) H_2O, CH_3CN solvent, 308 K).

Figure S22 shows preliminary C_4H_8O hydrolysis rate measurements exhibit combinations of 1st and 0th order on reactant concentrations, mimicking the behavior of **Figures 1 and 2** in the main text when ROH is the nucleophile. This implies that C_4H_8O hydrolysis proceeds through an analogous mechanism to that shown in **Scheme S1** and **Scheme 2** in the main text.

S12. Activity Coefficients Supplements

Activity coefficients were calculated using ChemCAD7. Briefly, reactant components (C_4H_8O, ROH, and CH_3CN) were added to the system using built-in thermophysical libraries. After choosing components, a K-value model which employs the UNIFAC method was selected to simulate a TP_{xy} diagram at a constant temperature. In any given simulation, the molar fraction of one component was fixed (e.g., C_4H_8O) while the other two components were allowed to vary. Mole fractions corresponding to subsets of experimental conditions were used to calculate mole fraction windows. Sufficient points (~5000) enabled finding the activity coefficients at the desired mole fractions. This process was repeated for each activity coefficient.

Table S3. Activity coefficients (γ), excess enthalpies (H^{ε}), excess entropies (S^{ε}), and excess free energies (G^{ε}) obtained with the UNIFAC method on ChemCAD (0.005 M C_4H_8O, 0.15 M ROH, CH_3CN solvent, 308 K).

	C_4H_8O				ROH	
ROH	γ	H^{ε} (kJ mol^{-1})	S^{ε} (J mol^{-1} K^{-1})	G^{ε} (kJ mol^{-1})	γ	G^{ε} (kJ mol^{-1})
methanol	2.22	1.01	-9.94	2.05	2.62	2.50
ethanol	2.21	1.10	-10.14	2.03	2.39	2.26
1-butanol	2.16	1.10	-9.99	1.97	2.93	2.80
1-hexanol	2.10	1.13	-9.86	1.90	3.74	3.44

Table S3 shows the activity coefficients and excess energies for ROH and C_4H_8O (in each ROH) at conditions consistent with the energetic discussion in **Section 3.2** in the main text. To compute the excess energies, the following equations were used:

$$G_i^{\varepsilon} = RT\ln(\gamma_i) \quad \text{(S15)}$$

$$H_i^{\varepsilon} = -\,RT^2\frac{d\ln(\gamma_i)}{dT} \quad \text{(S16)}$$

$$S_i^{\varepsilon} = \frac{H_i^{\varepsilon}-G_i^{\varepsilon}}{T} \quad \text{(S17)}$$

where R and T are the universal gas constant and temperature, respectively. The differential term in **Equation S16** was calculated by obtaining γ between 303-323 K in increments of 5 K. The differential term is equal to the slope of the natural log of these activity coefficients plotted against the temperature.

Alcohol activities are used to calculate rate constants (k_4) in **Figure 3** of the main text. The means and standard deviations of k_4 values were calculated to evaluate statistical significance of the data. First, t-scores were calculated using the following relation:

$$t = \frac{\bar{x}-\mu}{\frac{\sigma}{\sqrt{n}}} \quad \text{(S18)}$$

where $\bar{x}$ is the sample mean, μ is the mean for statistical testing, σ is the sample standard deviation, and n is the sample size. To evaluate the statistical significance of the k_4 values, t-score values and degrees of freedom $(n-1)$ were converted to p-values. **Table S4** summarizes the statistical test values below.

Table S4. Rate constant k_4 values from **Figure 3b** in the main text and associated statistical test values.

Catalyst	ROH	$\bar{x}_{k_4}$ (10^{-2})	σ_{k_4} (10^{-3})	t_{k_4} (ROH)	p_{k_4}*
Al-BEA	MeOH	1.03	1.25	--	--
Al-BEA	EtOH	0.89	1.27	-1.97 (MeOH)	0.094**
Al-BEA	1-BuOH	1.30	1.52	3.04 (MeOH)	0.047
Al-BEA	1-HxOH	1.49	2.44	3.26 (MeOH)	0.041
Al-BEA	1-BuOH	1.30	1.52	4.69 (EtOH)	0.021
Al-BEA	1-HxOH	1.49	2.44	4.29 (EtOH)	0.025
Al-BEA	1-HxOH	1.49	2.44	1.36 (BuOH)	0.153
Zr-BEA	MeOH	0.95	1.15	--	--
Zr-BEA	EtOH	0.58	0.51	-12.37 (MeOH)	0.003**
Zr-BEA	1-BuOH	0.61	0.82	-7.12 (MeOH)	0.010**
Zr-BEA	1-HxOH	0.85	1.33	-1.21 (MeOH)	0.175**
Zr-BEA	1-BuOH	0.61	0.82	0.54 (EtOH)	0.322
Zr-BEA	1-HxOH	0.85	1.33	3.49 (EtOH)	0.037
Zr-BEA	1-HxOH	0.85	1.33	3.16 (BuOH)	0.044

* One-tailed p-values are reported to evaluate if rate constants increase with increasing ROH chain length. ** Negative t-scores imply corresponding p-values correspond to rate constants decreasing.

Based on the p-values calculated in **Table S4**, statistically significant increases in k_4 occur from at least methanol to C_{4+} ROH and ethanol to C_{4+} ROH over Al-BEA. Similarly, statistically significant increases exist for at least ethanol to 1-hexanol and 1-butanol to 1-hexanol over Zr-BEA. Beyond these, some statistically significant decreases in rate constants exist (e.g., methanol to C_{2+} ROH over Zr-BEA). Together, these analyses highlight statistically significant changes to turnover rates which are not accounted for by alcohol activities alone.

S13. Activation Enthalpy and Entropy Calculations and Arrhenius Plots

Van't Hoff analysis was used to calculate apparent enthalpic ($\Delta H^{\ddagger}$) and entropic ($\Delta S^{\ddagger}$) barriers of total product turnover rates. The form of **Equation S12** can be expanded using transition state theory and replacing the concentration term with activity to yield:

$$\frac{r_{RO}}{[L]} = \frac{k_B T}{h} \exp\left(-\frac{\Delta G^{\ddagger}}{RT}\right) a_{ROH} \quad \text{(S19)}$$

Expanding the free energy in terms of enthalpy and entropy and rearranging yields:

$$\ln\left(\frac{r_{RO}}{[L]} \frac{h}{k_B T a_{ROH}}\right) = \frac{1}{T}\left(\frac{-\Delta H^{\ddagger}}{R}\right) + \frac{\Delta S^{\ddagger}}{R} \quad \text{(S20)}$$

The natural logarithm and inverse temperature terms of **Equation S20** are plotted below and fitted to a line. The slope and y-intercept of this line are equal to the negative of the apparent activation enthalpy divided by the ideal gas constant and the apparent activation entropy divided by the ideal gas constant, respectively (**Table S5**).

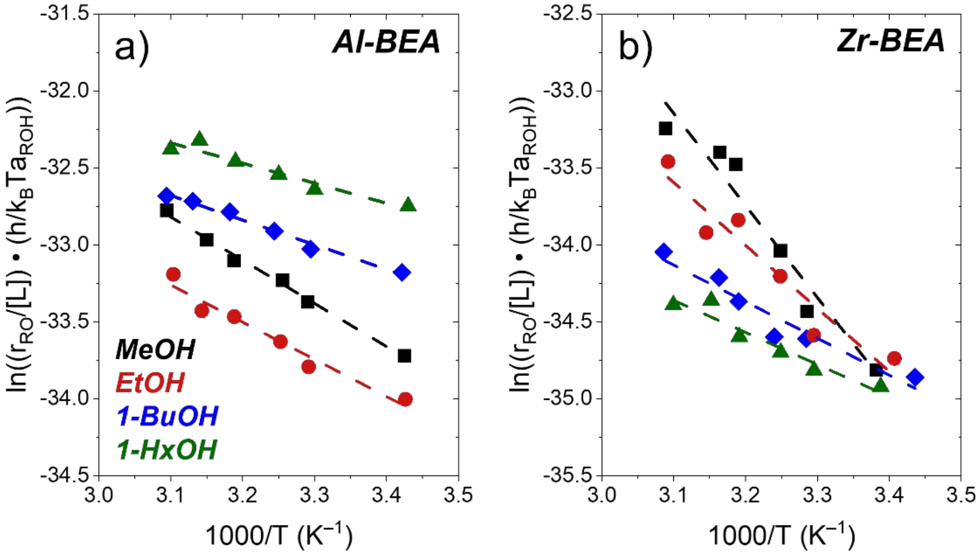

Figure S23. Eyring plots for C_4H_8O alcoholysis with methanol (black squares), ethanol (red circles), 1-butanol (blue diamonds), and 1-hexanol (green triangles) over a) Al-BEA and b) Zr-BEA (0.005 M C_4H_8O, 0.15 M CH_3OH, CH_3CN solvent, 290–323 K).

Table S5. Activation enthalpies and entropies extracted from the Eyring plots of **Figure S23**.

ROH	$\Delta H^{\ddagger}$ (kJ mol^{-1})		$\Delta S^{\ddagger}$ (J mol^{-1} K^{-1})	
--	Al-BEA	Zr-BEA	Al-BEA	Zr-BEA
MeOH	23.3 ± 0.9	49.8 ± 6.0	-200.5 ± 2.9	-121.3 ± 19.4
EtOH	20.1 ± 2.1	34.0 ± 5.1	-214.3 ± 6.7	-173.9 ± 16.5
1-BuOH	13.3 ± 0.8	19.8 ± 2.8	-230.4 ± 2.6	-222.3 ± 9.0
1-HxOH	10.9 ± 1.5	17.3 ± 2.5	-235.2 ± 4.7	-232.0 ± 8.0

S14. Extended Discussion of Energetic Contributions to Barriers

As summarized in Section 3.2 of the main text, activation enthalpies and entropies for Al- and Zr-BEA depend strongly on the ROH chain length. Here, we present an extended molecular interpretation of barriers to epoxide alcoholysis.

Differences between values of $\Delta H_4^{\ddagger}$ and $\Delta S_4^{\ddagger}$ for the distinct combinations of nucleophiles and zeolites reflect variations in individual terms in the states detailed in **Equation 9** of the main text. First, values of $G_{C_4H_8O^*}^{0}$ do not change with alcohol identity in comparisons among barriers measured over a given zeolite (e.g., Al-BEA). In these series, values of G_{ROH}^{0} depend on alcohol formation energies, which increase with alcohol size (MeOH to 1-HxOH: -240 to -378 kJ mol^{-1} ; **Table S2**).[39] Because the alcohol chain remains intact in the transition state, formation contributions are equivalently added to the transition state ($G^{\ddagger,0}$) to cancel any contribution from G_{ROH}^{0} on barrier trends. However, $G^{\ddagger,0}$ values also depend on the partial bond formed by nucleophilic attack (**Scheme 3** of main text, orange dotted lines). The strength of this bond is related to ROH nucleophilicity, which scales with basicity in aprotic solvent (omitting contributions from steric factors).[40] **Figure 4** shows enthalpic and entropic barriers follow linear dependencies on ROH proton affinities (a measure of basicity). Therefore, increasing the ROH chain length increases nucleophilicity, leading to a stronger alcohol-epoxide linkage at the transition state which causes enthalpic stabilization and concomitant entropic penalties in the standard state (i.e., within $G_4^{\ddagger,0}$).[41] However, nucleophilicity effects should be agnostic of the material, suggesting the large differences in the magnitudes of barrier trends over Al-BEA and Zr-BEA (**Figure 4**) must come from differences in solvation.

Excess free energy contributions from interactions with solvating molecules and the zeolite pore structure may also influence barrier trends in ways distinguishable from the covalent interactions of the reactive intermediates and at the transition states for ring-opening. The use of a_{ROH} to calculate rate constants accounts for non-idealities of alcohol molecules in the bulk (G_{ROH}^{ε}), and surface epoxide species likely experience negligible interactions with other ROH molecules because rate measurements utilized minimal [ROH] values and high volume fractions of CH_3CN (~99 mol% CH_3CN). Further, differences in excess enthalpy ($H_{C_4H_8O}^{\varepsilon}$) and entropy ($S_{C_4H_8O}^{\varepsilon}$) contributions of bulk fluid phase C_4H_8O molecules in reaction solutions vary much less than the $\Delta H_4^{\ddagger}$ and $\Delta S_4^{\ddagger}$ values in **Figure 4** (< 1 kJ mol^{-1} and < 1 J mol^{-1} K^{-1}, respectively; **Section S12**). As a result, values of $G_{C_4H_8O^*}^{\varepsilon}$ differ negligibly among experiments performed with different ROH reactants. With other possibilities excluded at the reaction conditions of **Figure 4**, only the interactions of solvent molecules and zeolite structures with transition states modify barrier trends (i.e., through $G_4^{\ddagger,\varepsilon}$).

Transition state solvation, captured by $G_4^{\ddagger,\varepsilon}$, includes contributions from transition states interacting with the zeolite pore and spectating solvent molecules. However, each class of interaction generally affects reaction barriers differently. For example, in alkene epoxidation over BEA zeolites it has been shown larger transition states (from longer chain ROH) interact to a greater extent with zeolite pore moieties, yielding less positive $\Delta H_4^{\ddagger}$ due to stabilizing van der Waals

interactions and more negative $\Delta S_4^{\ddagger}$ due to decreasing degrees of freedom.[42] **Figure S21** captures the same effect of more exothermic epoxide adsorption enthalpies as the zeolite pore size decreases. Conversely, larger transition states require greater rearrangement of intrapore solvent molecules and yield more positive $\Delta H_4^{\ddagger}$ and less negative $\Delta S_4^{\ddagger}$ due to interfering with solvent-zeolite interactions and increasing disorder, respectively.[42] Because measured enthalpic and entropic barriers in **Figure 4** systematically decrease with increasing ROH chain length, zeolite pore interactions are expected to dominate any excess contributions that exist beyond the effects of basicity. Notably, the dominance of zeolite pore interactions in this system contrasts with previous chain length studies that found increasing enthalpic and entropic barriers with increased chain length and attributed them to solvent rearrangement.[42] Here, active sites are covered by bulky C_4H_8O-derived intermediates which may occlude well-organized solvent species near active sites and reduce the contributions of solvent rearrangement during transition state formation. Enthalpic and entropic barriers are less sensitive to ROH chain length over Brønsted acidic Al-BEA compared to Lewis acidic Zr-BEA (**Figure 4**). Therefore, differences in enthalpic and entropic barrier sensitivity to ROH chain length may stem from either the propensity of protonated (Brønsted acid) and non-protonated (Lewis acid) transition states to interact with solvent molecules (e.g., *via* hydrogen bonding) or the zeolite pore structure (e.g., van der Waals forces). For example, protonated transition states in Al-BEA may reside at greater distances from the pore wall (due to proton linkages) than transition states in Zr-BEA, which may explain the larger entropic changes over Zr-BEA with increasing ROH chain length.

Increasing [ROH] will further influence energetics through changing the solvent composition near active sites as well as changing the dominant surface species. For example, under conditions of an ROH* MARI, excess contributions of surface ROH ($G^{\varepsilon}_{ROH^*}$) and fluid C_4H_8O ($G^{\varepsilon}_{C_4H_8O}$) are likely to contribute to reaction barriers alongside nucleophile bond strength and transition state solvation following arguments made previously.

S15. C_4H_8O Alcoholysis Regioselectivity Supplements

Regioselectivity is defined in the form of **Equation 10** in the main text and is reproduced below.

$$\beta = \frac{r_{TE}}{r_{TA}} = \frac{k_{i,TE}}{k_{i,TA}} = \exp\left(\frac{\Delta G^{\ddagger}_{TA} - \Delta G^{\ddagger}_{TE}}{RT}\right) = \exp\left(\frac{G^{\ddagger}_{TA} - G^{\ddagger}_{TE}}{RT}\right) \quad \text{(S21)}$$

Similar product rate dependencies on reactant concentrations suggest at all points where β is defined, concentration terms will cancel in **Equation S21** leaving only a ratio of rate constants. Furthermore, these rate constants are defined for steps stemming from the same reference state (i.e., either a C_4H_8O* or an ROH* species) and therefore the transition state energies are the only terms that do not cancel in the final form of **Equation S21**.

The β values shown in **Figure 5** in the main text are recast in terms of terminal ether selectivity (%, **Figure S24**) and individual reactant concentrations (**Figures S25 and S26**) below.

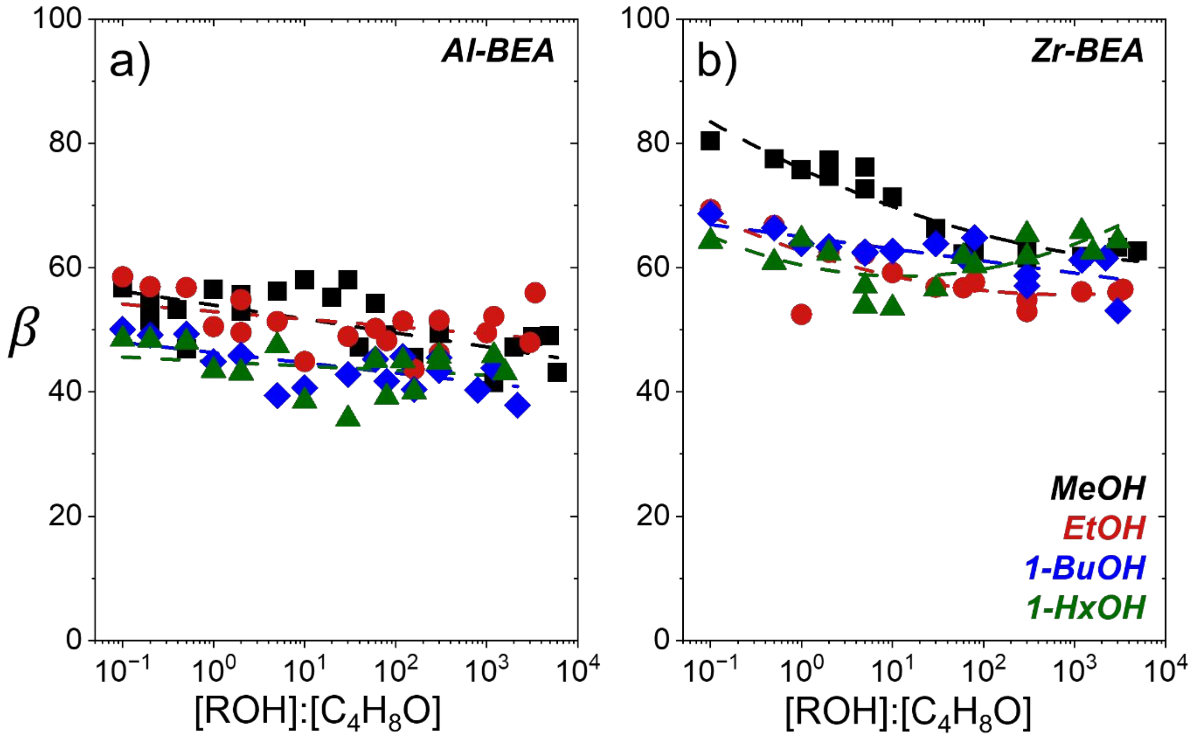

Figure S24. Terminal ether selectivities for C_4H_8O alcoholysis as functions of ROH to C_4H_8O concentration ratio (methanol (black), ethanol (red), 1-butanol (blue), 1-hexanol (green); 308 K) over a) Al-BEA and b) Zr-BEA. Methanol Al-BEA data adapted from previous work.[29]

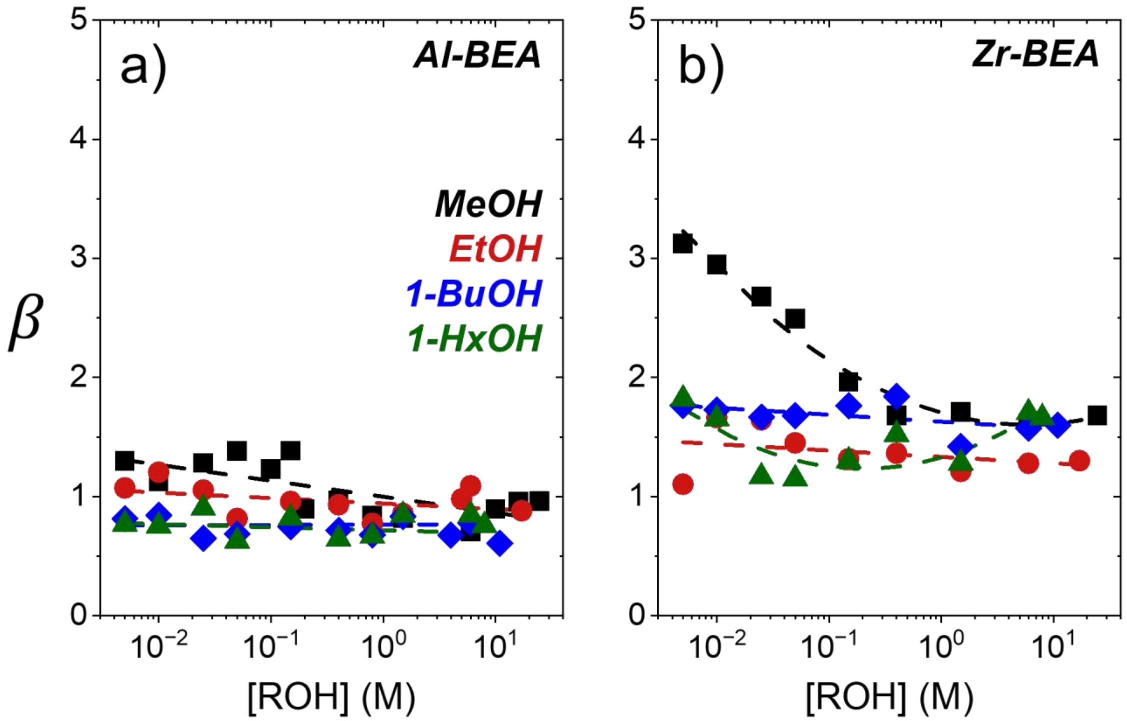

Figure S25. Regioselectivity (β) values for C_4H_8O alcoholysis as functions of ROH concentration (methanol (black), ethanol (red), 1-butanol (blue), 1-hexanol (green); 0.005 M C_4H_8O, CH_3CN solvent, 308 K) over a) Al-BEA and b) Zr-BEA. Methanol Al-BEA data adapted from previous work.[29]

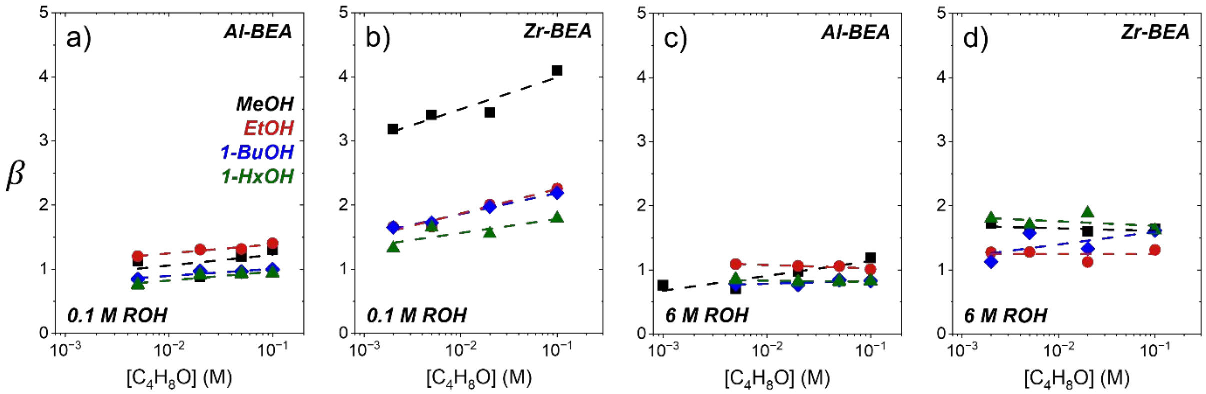

Figure S26. Regioselectivity (β) values for C_4H_8O alcoholysis as functions of C_4H_8O concentration (methanol (black), ethanol (red), 1-butanol (blue), 1-hexanol (green); 0.1 M ROH (a, b) or 6 M ROH (c, d), CH_3CN solvent, 308 K) over a,c) Al-BEA and b,d) Zr-BEA. Methanol Al-BEA data adapted from previous work.[29]

Figures S25 and S26 above show the β values for C_4H_8O alcoholysis are largely invariant with changing [ROH], except for in the case of methanol over Zr-BEA. Increasing [C_4H_8O] tends to increase β values when [ROH] is sufficiently low: at 6 M ROH (**Figure S26c,d**) β values are more constant than at 0.01 M ROH (**Figure S26a,b**).

The means and standard deviations of β values in **Figure 5** of the main text were calculated to perform statistical tests. From these data, t-scores were calculated using **Equation S18**. To

evaluate the statistical significance of the average β values, t-score values and degrees of freedom $(n-1)$ were converted to p-values. **Table S6 and S7** summarize the statistical test values below.

Table S6. Average Al-BEA β values from **Figure 5a** in the main text and associated statistical test values.

ROH	$\bar{x}_\beta$	σ_β	n	t_β (methanol)	p_β (methanol)	t_β (1-hexanol)	p_β (1-hexanol)
methanol	1.04	0.22	22	--	--	4.818	< 0.001
ethanol	1.05	0.18	19	0.318	0.754	5.683	< 0.001
1-butanol	0.81	0.11	19	-8.925	< 0.001	-0.220	0.828
1-hexanol	0.81	0.10	18	-9.466	< 0.001	--	--

Table S7. Average Zr-BEA β values from **Figure 5b** in the main text and associated statistical test values.

ROH	$\bar{x}_\beta$	σ_β	n	t_β (methanol)	p_β (methanol)	t_β (1-hexanol)	p_β (1-hexanol)
methanol	2.38	0.84	17	--	--	4.064	< 0.001
ethanol	1.47	0.31	17	-12.191	< 0.001	-1.069	0.301
1-butanol	1.62	0.24	17	-12.980	< 0.001	1.313	0.208
1-hexanol	1.55	0.24	17	-14.535	< 0.001	--	--

The p-values calculated in **Tables S6 and S7** provide evidence of statistically significant decreases in the average value of β when the ROH chain length increases. In the case of Al-BEA, β values decrease from methanol to 1-butanol and no statistically significant change occurs between methanol and ethanol or between 1-butanol and 1-hexanol. In the case of Zr-BEA, average β values decrease from methanol to ethanol. However, ethanol and 1-hexanol do not have statistically significant values of β over Zr-BEA, implying insignificant changes to β when increasing ROH chain length longer than ethanol.

S16. H_2O-Cosolvent Regioselectivity Statistical Discussion

Figure 6 of the main text depicts linear dependencies of β values on the amount of H_2O cosolvent present. **Tables S8 and S9** below summarize the linear slope and associated standard error of each data series in **Figure 6**. The 95% confidence intervals calculated in the tables below suggest statistically significant slope changes occur as the ROH chain length increases from MeOH to 1-HxOH over both Al-BEA and Zr-BEA.

Table S8. Linear slopes from **Figure 6a** in the main text and associated statistical test values.

ROH	$\overline{m}$	SE	95% CI (low)	95% CI (high)
methanol	-0.39	0.07	-0.53	-0.24
ethanol	-0.29	0.02	-0.34	-0.24
1-butanol	-0.18	0.02	-0.22	-0.14
1-hexanol	0.11	0.07	-0.02	0.24

Table S9. Linear slopes from **Figure 6b** in the main text and associated statistical test values.

ROH	$\overline{m}$	SE	95% CI (low)	95% CI (high)
methanol	-0.82	0.08	-0.97	-0.67
ethanol	-0.55	0.18	-0.90	-0.20
1-butanol	0.03	0.09	-0.15	0.22
1-hexanol	0.21	0.02	0.17	0.25

Residual H_2O content in the CH_3CN solvent which makes up >97 % of reactant mixtures in **Figure 6** was evaluated *via* ^{1}H NMR. The reactant grade of the CH_3CN suggests less than 0.005 M H_2O upon delivery. To estimate the H_2O which entered the solvent upon usage in the lab, ^{1}H spectra of CD_3CN (Sigma Aldrich, MagniSolv 99%) and CH_3CN/CD_3CN mixtures were collected. The peak areas for H_2O (2.13 ppm) were measured in relation to an internal standard (benzene, 7.37 ppm).[43] Peak areas were converted to molar quantities of H_2O *via* GC-FID analyses of internal standard concentrations. The residual H_2O content in the CH_3CN solvent was calculated to be 0.022 M. The data in **Figure 6** account for residual H_2O content in CH_3CN, however, omitting the solvent H_2O content would not remove the statistical significance of slope changes shown in **Tables S8 and S9** above.

S17. Representative Batch Reaction Plots to Validate Lack of Conversion Effects

Figures S27 and S28 below depict representative batch reaction concentration vs. time profiles to demonstrate the effect of conversion (i.e., greater time) does not greatly alter ring-opening regioselectivities in the present system. Specifically in **Figures S27 and S28**, product concentration ratios remain within less than 4% of the final product ratio and regioselectivities (calculated from 3 to 6 timepoints) have a deviation of less than 9% from the full 6 timepoint fit.

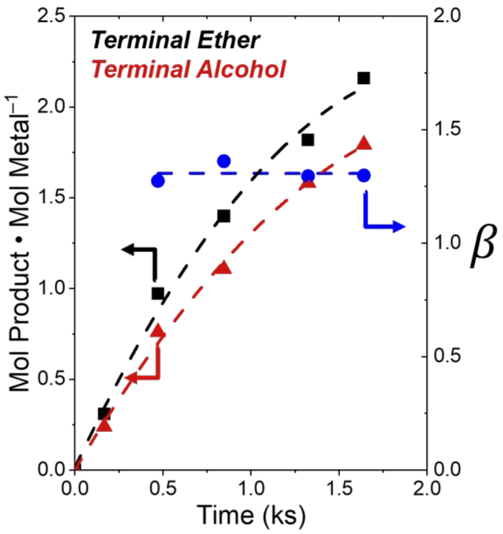

Figure S27. Representative low conversion (~0.05) batch reaction molar evolution as a function of time for the terminal ether (black, square) and terminal alcohol (red, triangle) products over Zr-BEA (0.005 M C_4H_8O, 6 M EtOH, CH_3CN solvent, 308 K). Regioselectivities (blue, circle) are calculated from subsets of timepoints ranging from 0 to the depicted time.

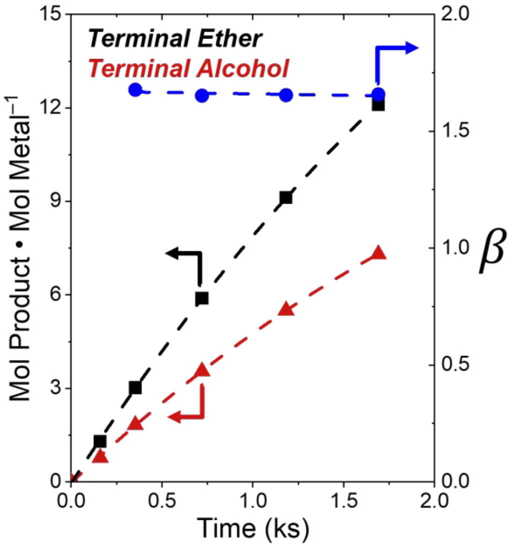

Figure S28. Representative high conversion (~0.5) batch reaction molar evolution as a function of time for the terminal ether (black, square) and terminal alcohol (red, triangle) products over Al-BEA (0.005 M C_4H_8O, 17 M EtOH, 308 K). Regioselectivities (blue, circle) are calculated from subsets of timepoints ranging from 0 to the depicted time.

S18. Isothermal Titration Calorimetry Thermograms and Heats per Injection

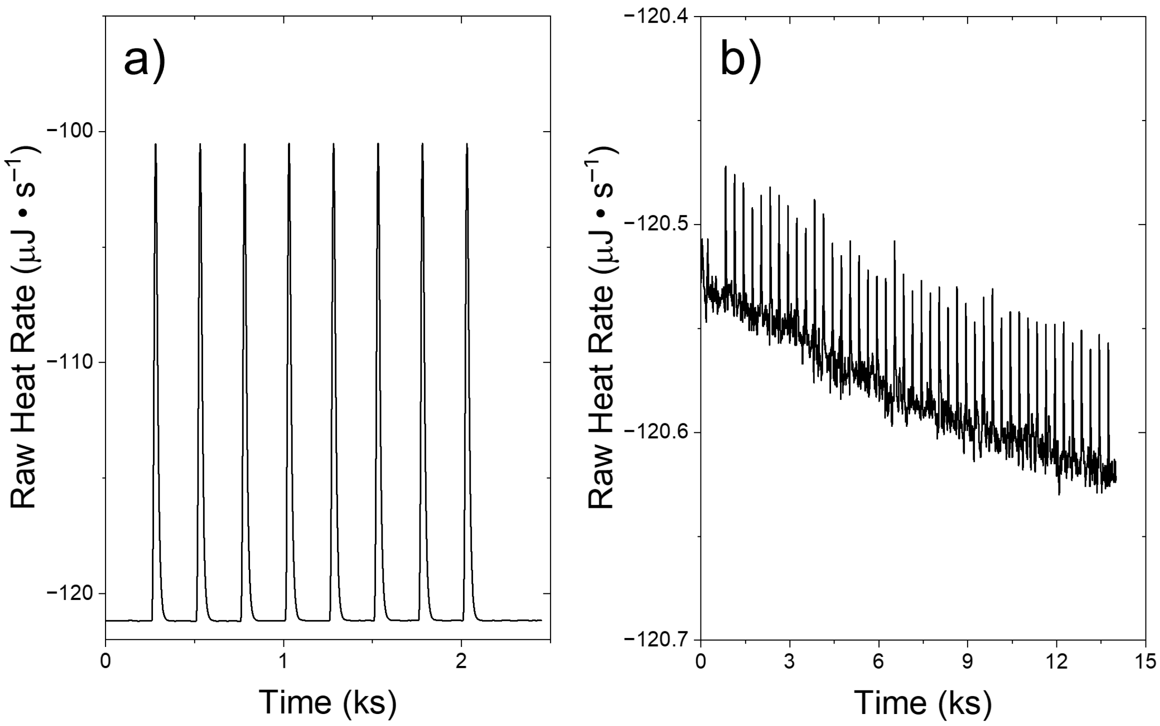

Figure S29. ITC thermograms from a) electrical calibration of the NanoITC instrument and b) water-water adsorption to verify cell cleanliness.

Figure S29 shows the results of instrument calibration and verification of cell cleanliness for isothermal titration calorimetry (ITC) experiments described in **Section 2.4** of the main text. Electrical calibration is performed to yield an instrument calibration factor and success was assumed as long as calibration values exceeded 0.99. **Figure S29b** shows the results of the water-water injection verification, which resulted in absolute peak areas below 3 μJ for each 2.5 μL injection.

The remaining **Figures S30–S40** depict the thermograms and corresponding heats released as a function of coverage for the adsorption measurements depicted in **Figure S21**. Average heats released were calculated from data points depicted in black. Outlier data points depicted in grey are likely due to evaporation or diffusion from the syringe resulting in titrant quantity inaccuracies.

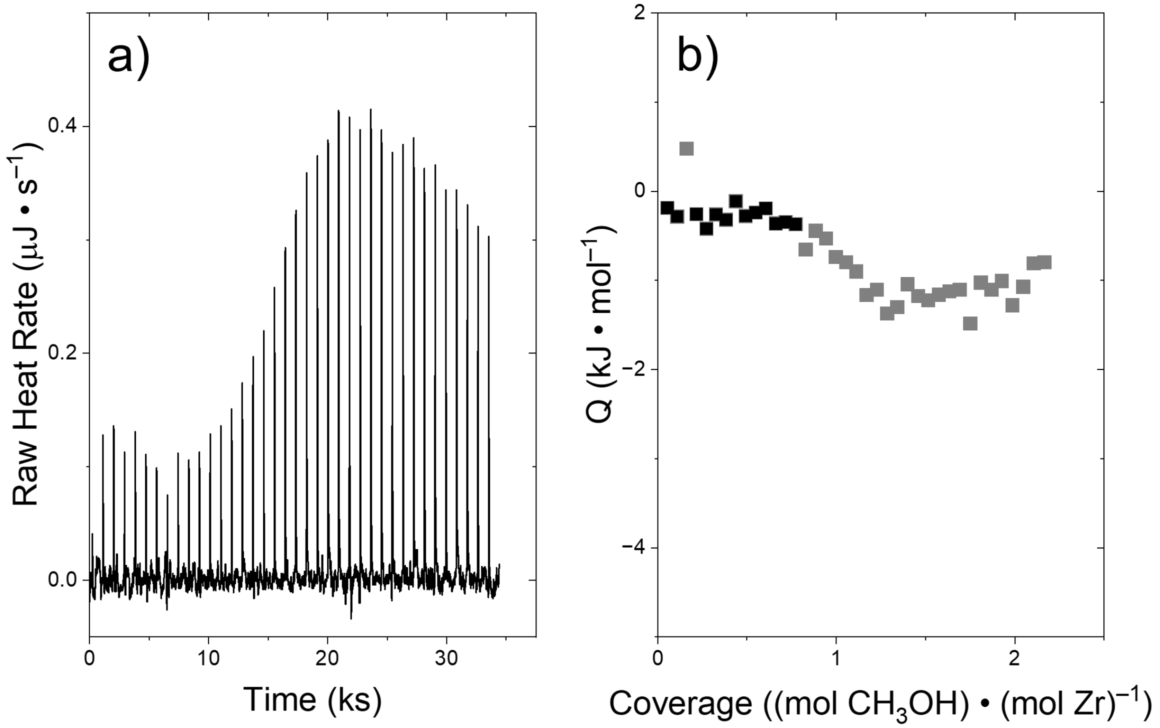

Figure S30. ITC a) thermogram and b) associated heats released as a function of titrant injected for the titration of Zr-BEA with MeOH (0.005 M MeOH in CH3CN, 308 K, 2.5 μL per injection).

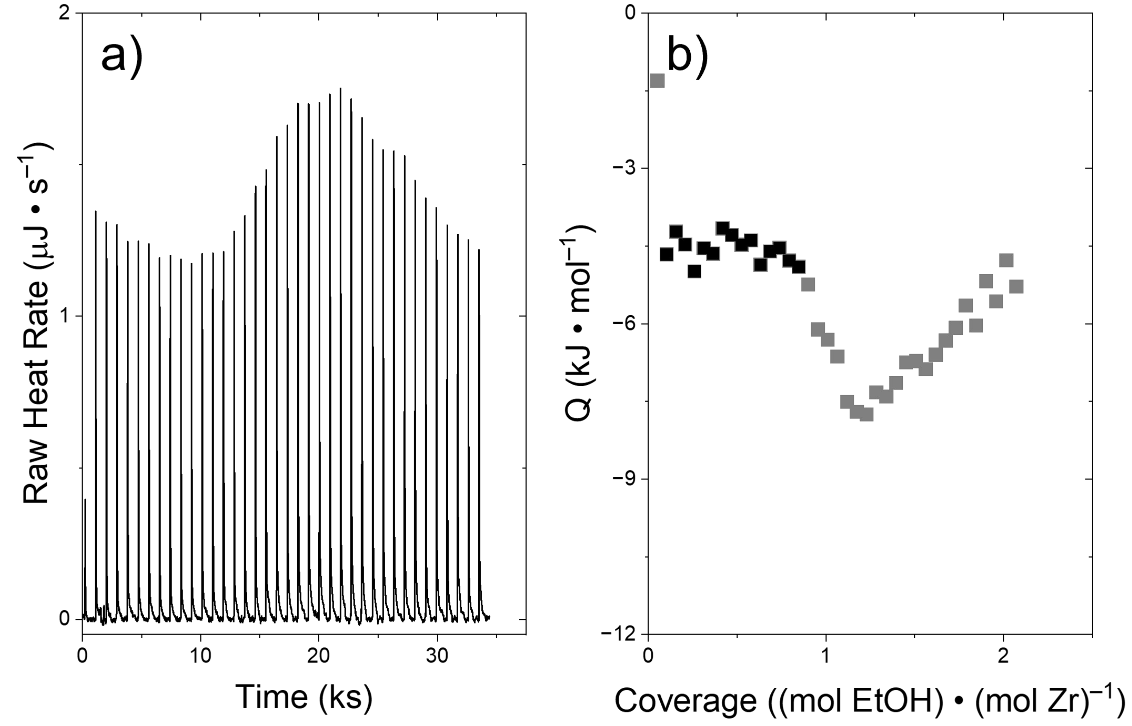

Figure S31. ITC a) thermogram and b) associated heats released as a function of titrant injected for the titration of Zr-BEA with EtOH (0.005 M EtOH in CH3CN, 308 K, 2.5 μL per injection).

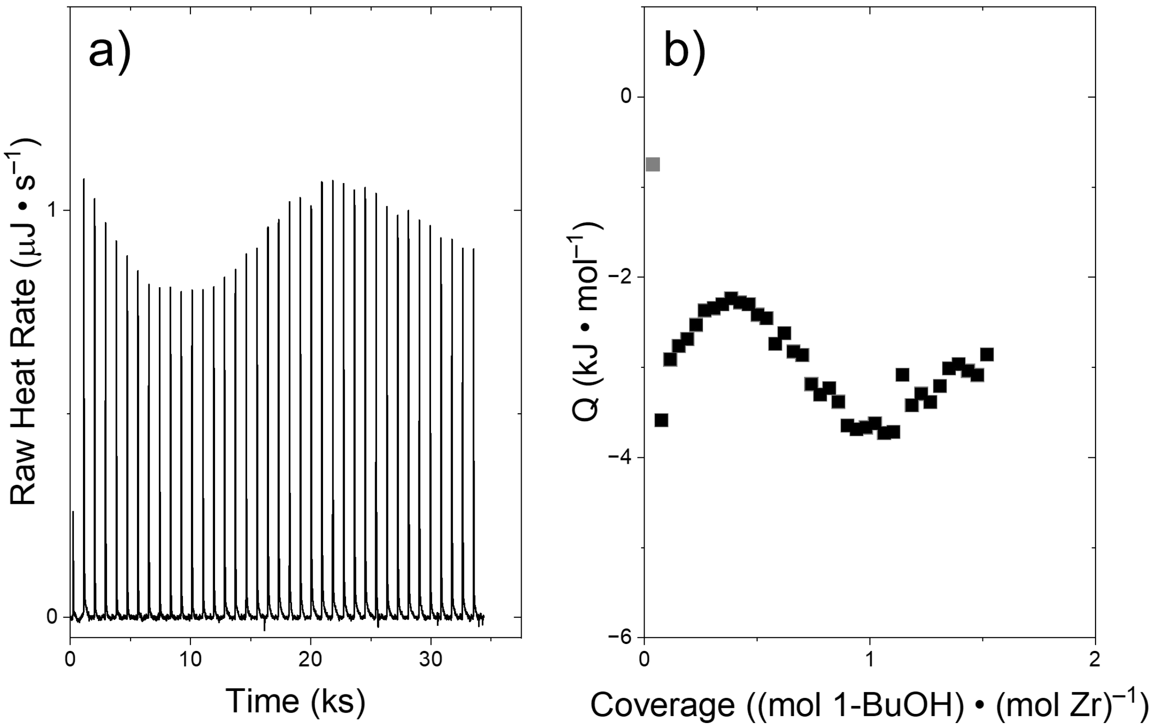

Figure S32. ITC a) thermogram and b) associated heats released as a function of titrant injected for the titration of Zr-BEA with 1-BuOH (0.005 M 1-BuOH in CH3CN, 308 K, 2.5 µL per injection).

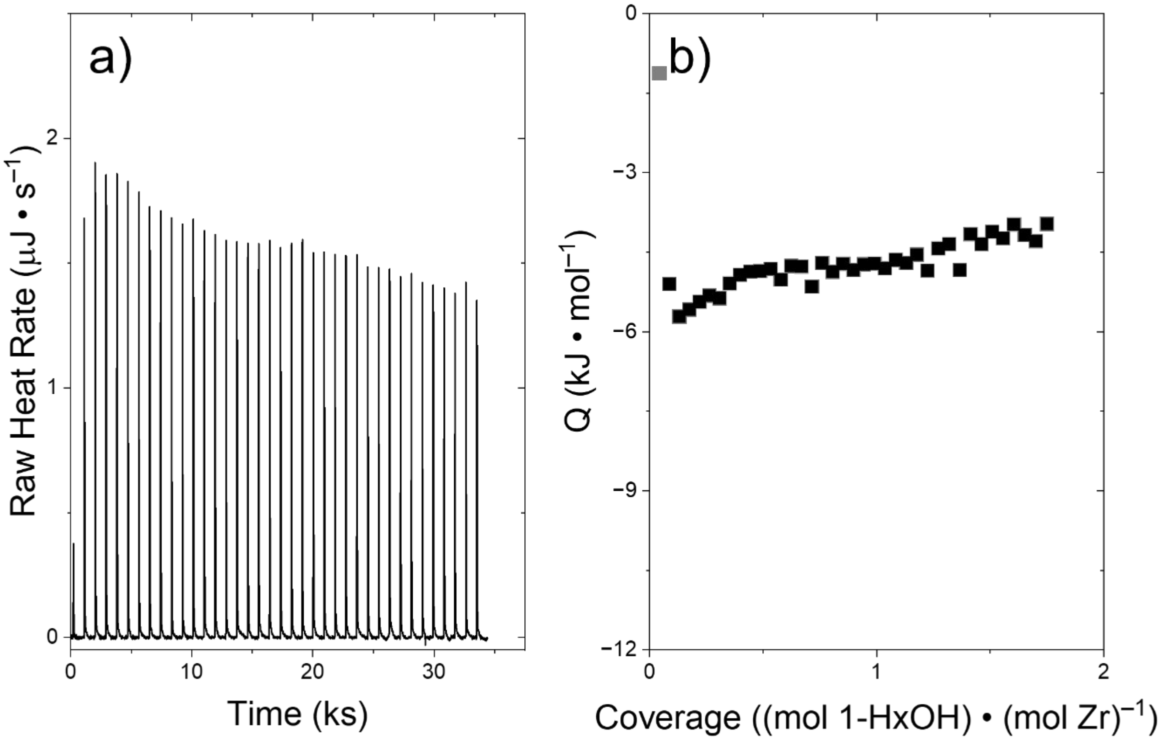

Figure S33. ITC a) thermogram and b) associated heats released as a function of titrant injected for the titration of Zr-BEA with 1-HxOH (0.005 M 1-HxOH in CH3CN, 308 K, 2.5 µL per injection).

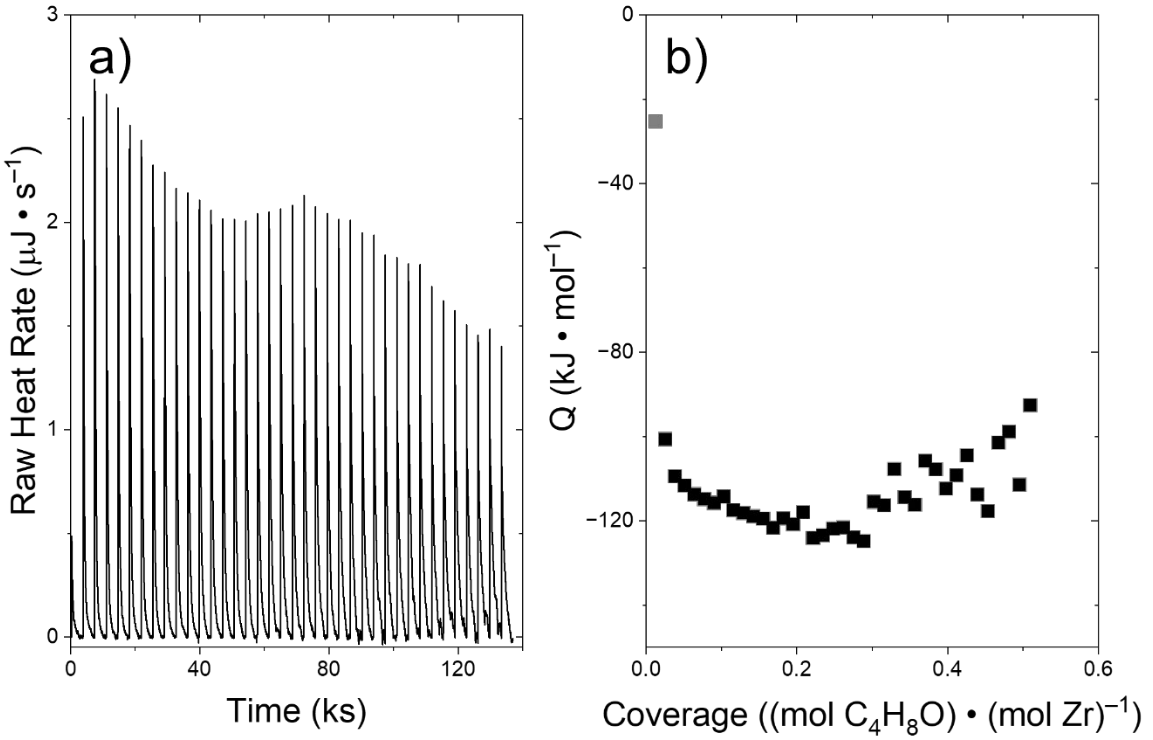

Figure S34. ITC a) thermogram and b) associated heats released as a function of titrant injected for the titration of Zr-BEA with C_4H_8O (0.005 M C_4H_8O in CH3CN, 308 K, 2.5 μL per injection).

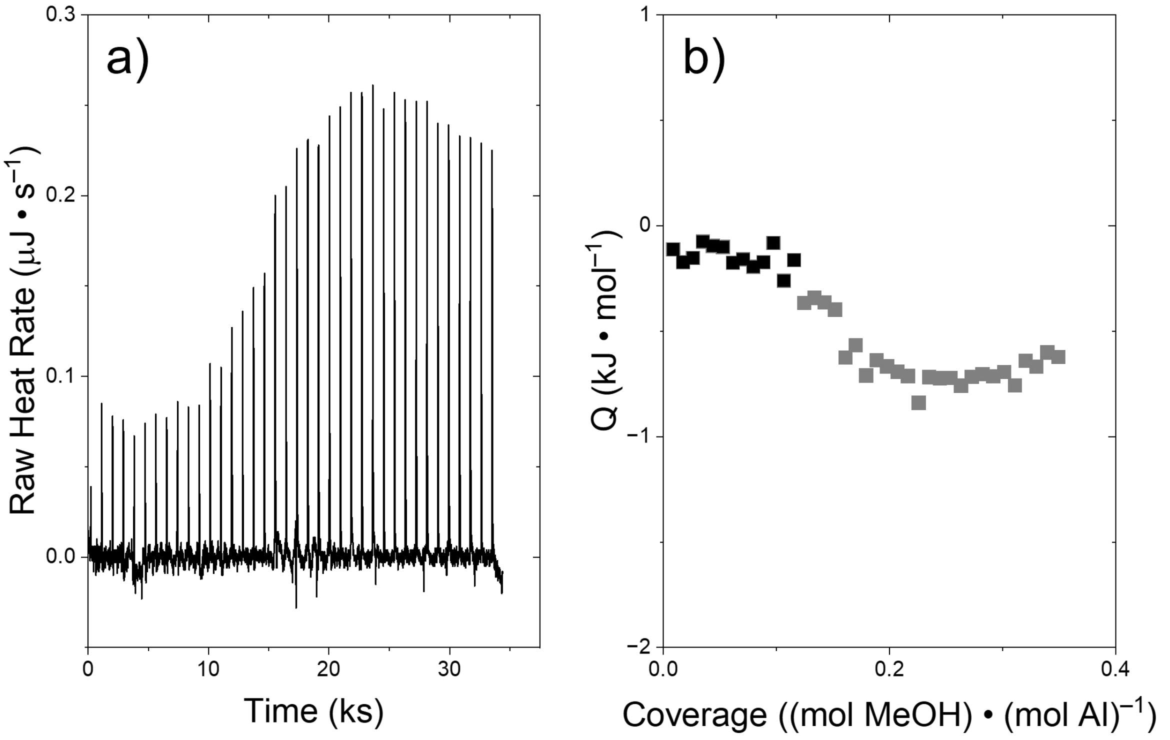

Figure S35. ITC a) thermogram and b) associated heats released as a function of titrant injected for the titration of Al-BEA with MeOH (0.005 M MeOH in CH3CN, 308 K, 2.5 μL per injection).

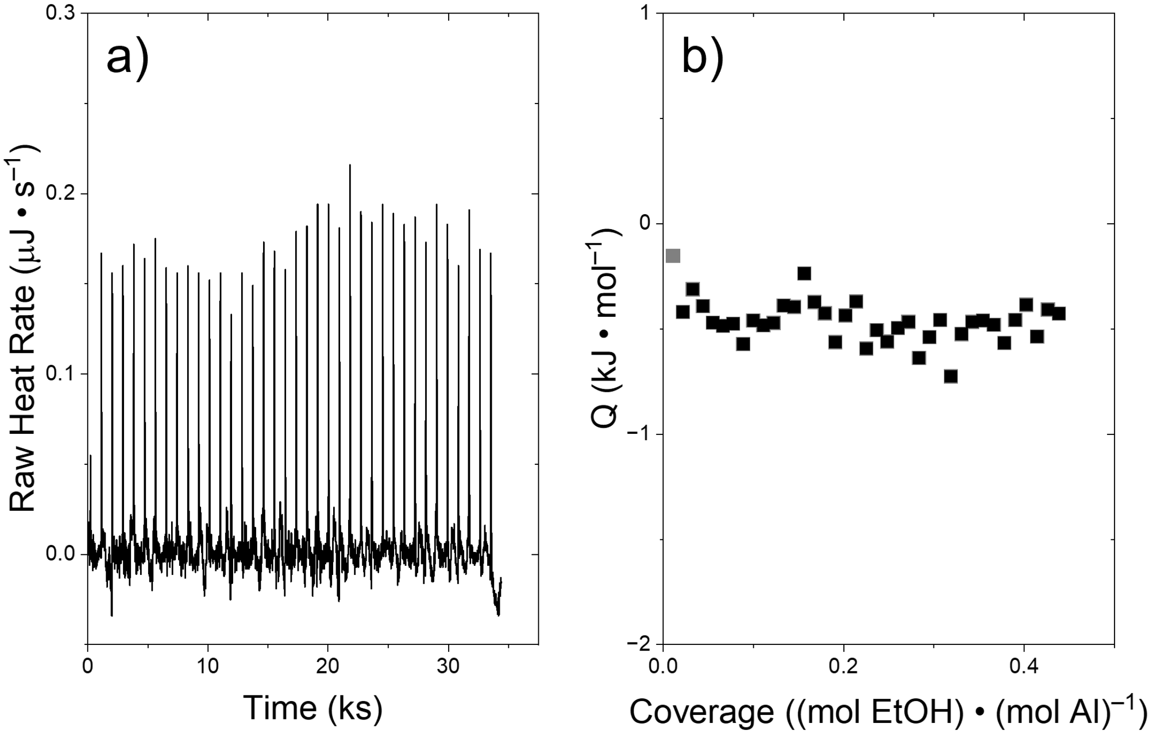

Figure S36. ITC a) thermogram and b) associated heats released as a function of titrant injected for the titration of Al-BEA with EtOH (0.005 M EtOH in CH3CN, 308 K, 2.5 μL per injection).

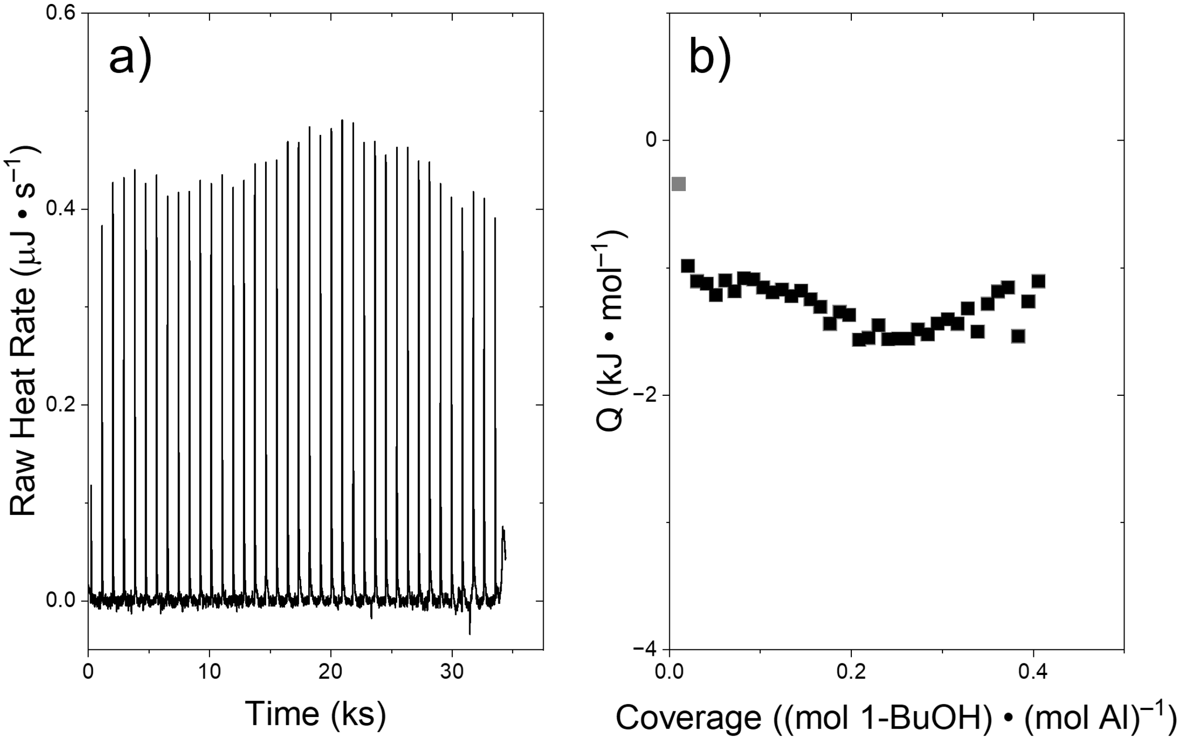

Figure S37. ITC a) thermogram and b) associated heats released as a function of titrant injected for the titration of Al-BEA with 1-BuOH (0.005 M 1-BuOH in CH3CN, 308 K, 2.5 μL per injection).

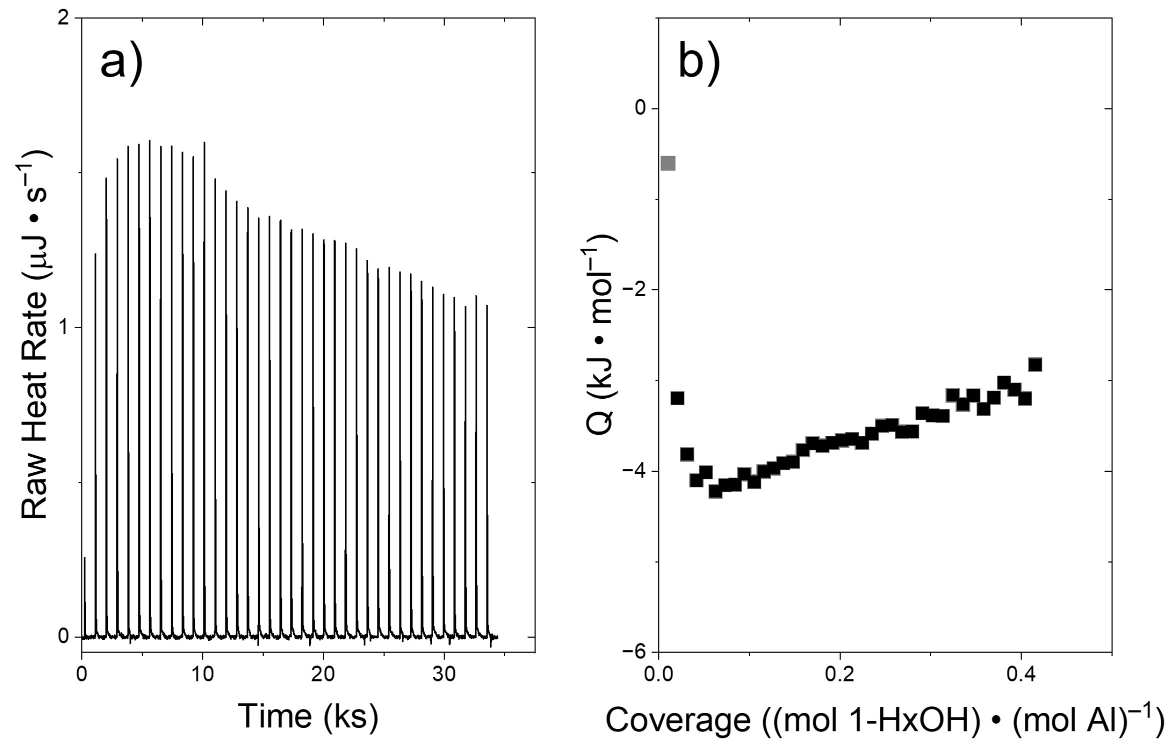

Figure S38. ITC a) thermogram and b) associated heats released as a function of titrant injected for the titration of Al-BEA with 1-HxOH (0.005 M 1-HxOH in CH3CN, 308 K, 2.5 μL per injection).

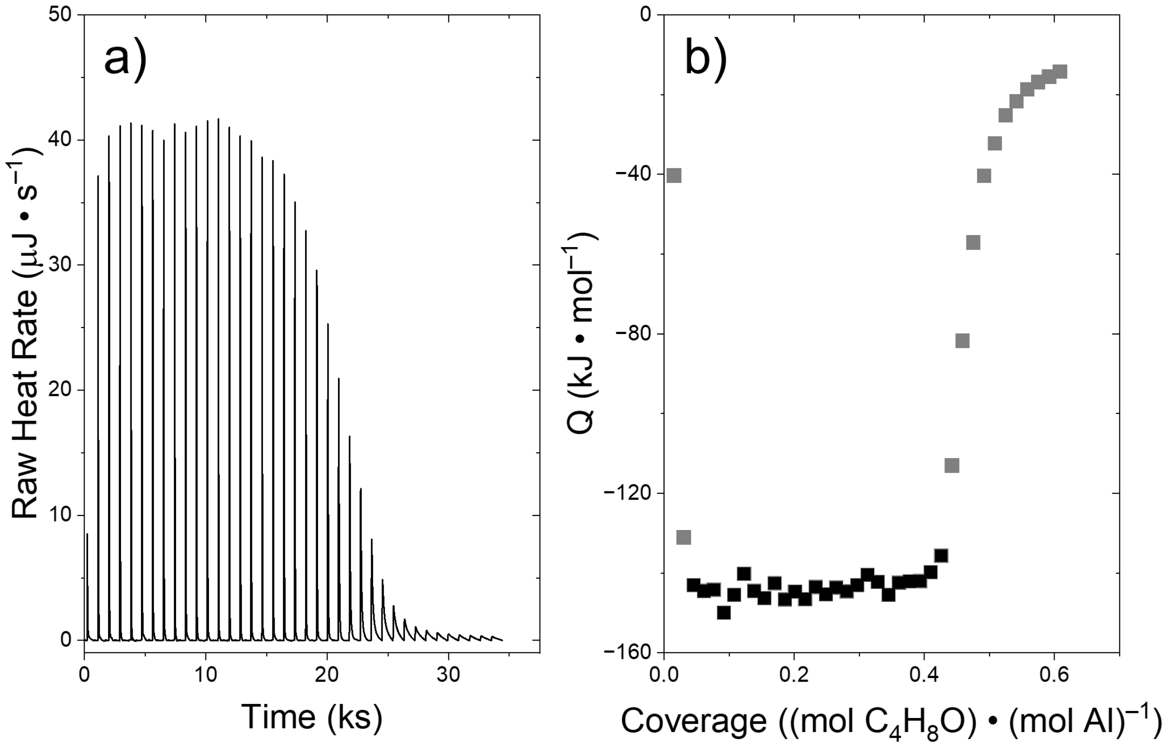

Figure S39. ITC a) thermogram and b) associated heats released as a function of titrant injected for the titration of Al-BEA with C_4H_8O (0.005 M C_4H_8O in CH3CN, 308 K, 2.5 μL per injection).

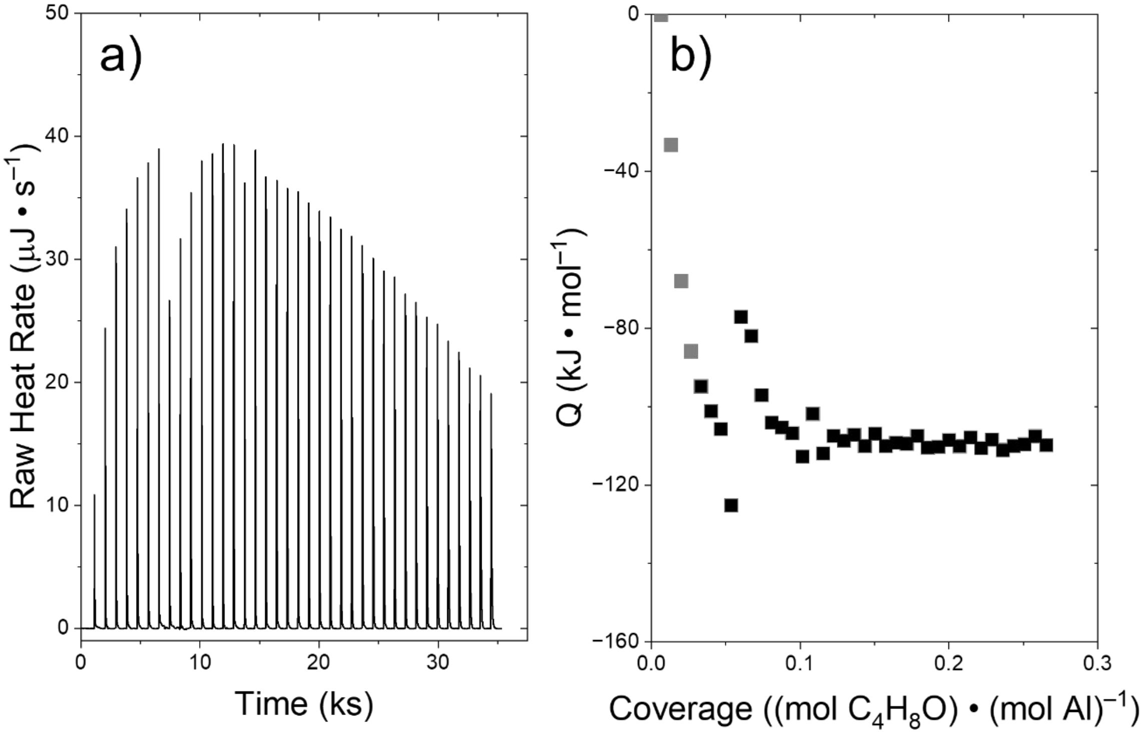

Figure S40. ITC a) thermogram and b) associated heats released as a function of titrant injected for the titration of Al-FAU (Thermo Scientific, Si/Al = 120) with C_4H_8O (0.005 M C_4H_8O in CH3CN, 308 K, 2.5 μL per injection).

References

(1) Newsam, J. M.; Treacy, M. M. J.; Koetsier, W. T.; Gruyter, C. B. D.; Thomas, J. M. Structural characterization of zeolite beta. *Proceedings of the Royal Society of London. A. Mathematical and Physical Sciences* **1988**, *420* (1859), 375-405. DOI: doi:10.1098/rspa.1988.0131.

(2) Filatova, E. O.; Konashuk, A. S. Interpretation of the Changing the Band Gap of Al2O3 Depending on Its Crystalline Form: Connection with Different Local Symmetries. *The Journal of Physical Chemistry C* **2015**, *119* (35), 20755-20761. DOI: 10.1021/acs.jpcc.5b06843.

(3) Mo, S.-D.; Xu, Y.-N.; Ching, W.-Y. Electronic and Structural Properties of Bulk γ-Al2O3. *Journal of the American Ceramic Society* **1997**, *80* (5), 1193-1197. DOI: 10.1111/j.1151-2916.1997.tb02963.x.

(4) Tosheva, L.; Mihailova, B.; Valtchev, V.; Sterte, J. Zeolite beta spheres. *Microporous and Mesoporous Materials* **2001**, *48* (1), 31-37. DOI: 10.1016/S1387-1811(01)00327-4.

(5) Mihailova, B.; Valtchev, V.; Mintova, S.; Faust, A. C.; Petkov, N.; Bein, T. Interlayer stacking disorder in zeolite beta family: a Raman spectroscopic study. *Physical Chemistry Chemical Physics* **2005**, *7* (14), 2756-2763. DOI: 10.1039/B503150H.

(6) Inagaki, S.; Nakatsuyama, K.; Saka, Y.; Kikuchi, E.; Kohara, S.; Matsukata, M. Elucidation of Medium-Range Structure in a Dry Gel-Forming *BEA-Type Zeolite. *The Journal of Physical Chemistry C* **2007**, *111* (28), 10285-10293. DOI: 10.1021/jp0668044.

(7) Majano, G.; Mintova, S.; Ovsitser, O.; Mihailova, B.; Bein, T. Zeolite Beta nanosized assemblies. *Microporous and Mesoporous Materials* **2005**, *80* (1), 227-235. DOI: 10.1016/j.micromeso.2004.12.019.

(8) Knops-Gerrits, P.-P.; De Vos, D. E.; Feijen, E. J. P.; Jacobs, P. A. Raman spectroscopy on zeolites. *Microporous Materials* **1997**, *8* (1), 3-17. DOI: 10.1016/S0927-6513(96)00088-0.

(9) Yu, Y.; Xiong, G.; Li, C.; Xiao, F.-S. Characterization of aluminosilicate zeolites by UV Raman spectroscopy. *Microporous and Mesoporous Materials* **2001**, *46* (1), 23-34. DOI: 10.1016/S1387-1811(01)00271-2.

(10) Aminzadeh, A. Excitation Frequency Dependence and Fluorescence in the Raman Spectra of Al2O3. *Applied Spectroscopy* **1997**, *51* (6), 817-819. DOI: 10.1366/0003702971941331.

(11) Aminzadeh, A.; Sarikhani-fard, H. Raman spectroscopic study of Ni/Al2O3 catalyst. *Spectrochimica Acta Part A: Molecular and Biomolecular Spectroscopy* **1999**, *55* (7), 1421-1425. DOI: 10.1016/S1386-1425(98)00312-6.

(12) Porto, S. P. S.; Krishnan, R. S. Raman Effect of Corundum. *The Journal of Chemical Physics* **1967**, *47* (3), 1009-1012. DOI: 10.1063/1.1711980.

(13) Purohit, R. D.; Saha, S.; Tyagi, A. K. Combustion synthesis of nanocrystalline ZrO2 powder: XRD, Raman spectroscopy and TEM studies. *Materials Science and Engineering: B* **2006**, *130* (1), 57-60. DOI: 10.1016/j.mseb.2006.02.041.

(14) Yi, M.; Zhang, Y.; Xu, J.; Deng, D.; Mao, Z.; Meng, X.; Shi, X.; Zhao, B. Surface-Enhanced Raman Scattering Activity of ZrO2 Nanoparticles: Effect of Tetragonal and Monoclinic Phases. *Nanomaterials* **2021**, *11* (9), 2162. DOI: 10.3390/nano11092162.

(15) Kim, D.-J.; Jung, H.-J.; Yang, I.-S. Raman Spectroscopy of Tetragonal Zirconia Solid Solutions. *Journal of the American Ceramic Society* **1993**, *76* (8), 2106-2108. DOI: 10.1111/j.1151-2916.1993.tb08341.x.

(16) Li, C.; Xiong, G.; Liu, J.; Ying, P.; Xin, Q.; Feng, Z. Identifying Framework Titanium in TS-1 Zeolite by UV Resonance Raman Spectroscopy. *The Journal of Physical Chemistry B* **2001**, *105* (15), 2993-2997. DOI: 10.1021/jp0042359.

(17) Bordiga, S.; Damin, A.; Bonino, F.; Ricchiardi, G.; Zecchina, A.; Tagliapietra, R.; Lamberti, C. Resonance Raman effects in TS-1: the structure of Ti(iv) species and reactivity towards H2O, NH3 and H2O2: an in situ study. *Physical Chemistry Chemical Physics* **2003**, *5* (20), 4390-4393, 10.1039/B306041C. DOI: 10.1039/B306041C.
(18) Signorile, M.; Crocellà, V.; Damin, A.; Rossi, B.; Lamberti, C.; Bonino, F.; Bordiga, S. Effect of Ti Speciation on Catalytic Performance of TS-1 in the Hydrogen Peroxide to Propylene Oxide Reaction. *The Journal of Physical Chemistry C* **2018**, *122* (16), 9021-9034. DOI: 10.1021/acs.jpcc.8b01401.
(19) Ricchiardi, G.; Damin, A.; Bordiga, S.; Lamberti, C.; Spanò, G.; Rivetti, F.; Zecchina, A. Vibrational Structure of Titanium Silicate Catalysts. A Spectroscopic and Theoretical Study. *Journal of the American Chemical Society* **2001**, *123* (46), 11409-11419. DOI: 10.1021/ja010607v.
(20) Zhao, Z.; Xu, S.; Hu, M. Y.; Bao, X.; Peden, C. H. F.; Hu, J. Investigation of Aluminum Site Changes of Dehydrated Zeolite H-Beta during a Rehydration Process by High-Field Solid-State NMR. *The Journal of Physical Chemistry C* **2015**, *119* (3), 1410-1417. DOI: 10.1021/jp509982r.
(21) van Bokhoven, J. A.; van der Eerden, A. M. J.; Koningsberger, D. C. Flexible aluminium coordination of zeolites as function of temperature and water content, an in-situ method to determine aluminium coordinations. In *Studies in Surface Science and Catalysis*, Aiello, R., Giordano, G., Testa, F. Eds.; Vol. 142; Elsevier, 2002; pp 1885-1890.
(22) Wouters, B. H.; Chen, T. H.; Grobet, P. J. Reversible Tetrahedral−Octahedral Framework Aluminum Transformation in Zeolite Y. *Journal of the American Chemical Society* **1998**, *120* (44), 11419-11425. DOI: 10.1021/ja982082l.
(23) Chen, X.; Yu, T. Simulating Crystal Structure, Acidity, Proton Distribution, and IR Spectra of Acid Zeolite HSAPO-34: A High Accuracy Study. *Molecules* **2023**, *28* (24), 8087. DOI: 10.3390/molecules28248087.
(24) Kester, P. M.; Crum, J. T.; Li, S.; Schneider, W. F.; Gounder, R. Effects of Brønsted acid site proximity in chabazite zeolites on OH infrared spectra and protolytic propane cracking kinetics. *Journal of Catalysis* **2021**, *395*, 210-226. DOI: 10.1016/j.jcat.2020.12.038.
(25) Ward, J. W. A spectroscopic study of the surface of zeolite Y: the adsorption of pyridine. *Journal of Colloid and Interface Science* **1968**, *28* (2), 269-278. DOI: 10.1016/0021-9797(68)90130-6.
(26) Lefrancois, M.; Malbois, G. The nature of the acidic sites on mordenite: Characterization of adsorbed pyridine and water by infrared study. *Journal of Catalysis* **1971**, *20* (3), 350-358. DOI: 10.1016/0021-9517(71)90097-2.
(27) Kataoka, T.; Dumesic, J. A. Acidity of unsupported and silica-supported vanadia, molybdena, and titania as studied by pyridine adsorption. *Journal of Catalysis* **1988**, *112* (1), 66-79. DOI: 10.1016/0021-9517(88)90121-2.
(28) Parry, E. P. An infrared study of pyridine adsorbed on acidic solids. Characterization of surface acidity. *Journal of Catalysis* **1963**, *2* (5), 371-379. DOI: 10.1016/0021-9517(63)90102-7.
(29) Potts, D. S.; Komar, J. K.; Locht, H.; Flaherty, D. W. Understanding Rates and Regioselectivities for Epoxide Methanolysis within Zeolites: Mechanism and Roles of Covalent and Non-covalent Interactions. *ACS Catalysis* **2023**, *13* (22), 14928-14944. DOI: 10.1021/acscatal.3c04103.
(30) Scanlon, J. T.; Willis, D. E. Calculation of Flame Ionization Detector Relative Response Factors Using the Effective Carbon Number Concept. *Journal of Chromatographic Science* **1985**, *23* (8), 333-340. DOI: 10.1093/chromsci/23.8.333.

(31) Jorgensen, A. D.; Picel, K. C.; Stamoudis, V. C. Prediction of gas chromatography flame ionization detector response factors from molecular structures. *Analytical Chemistry* **1990**, *62* (7), 683-689. DOI: 10.1021/ac00206a007.
(32) Potts, D. S.; Komar, J. K.; Jacobson, M. A.; Locht, H.; Flaherty, D. W. Consequences of Pore Polarity and Solvent Structure on Epoxide Ring-Opening in Lewis and Brønsted Acid Zeolites. *JACS Au* **2024**, *4* (9), 3501-3518. DOI: 10.1021/jacsau.4c00398.
(33) Marcus, Y. The effectiveness of solvents as hydrogen bond donors. *Journal of Solution Chemistry* **1991**, *20* (9), 929-944. DOI: 10.1007/BF01074953.
(34) Gude, M.; Teja, A. S. Vapor-liquid critical properties of elements and compounds. 4. Aliphatic alkanols. *Journal of Chemical and Engineering Data* **1995**, *40* (5), 1025-1036. DOI: 10.1021/je00021a001.
(35) Hunter, E. P. L.; Lias, S. G. Evaluated Gas Phase Basicities and Proton Affinities of Molecules: An Update. *Journal of Physical and Chemical Reference Data* **1998**, *27* (3), 413-656. DOI: 10.1063/1.556018.
(36) Chao, J.; Rossini, F. Heats of Combustion, Formation, and Isomerization of Nineteen Alkanols. *Journal of Chemical and Engineering Data* **1965**, *10* (4), 374-379. DOI: 10.1021/je60027a022.
(37) Mosselman, C.; Dekker, H. Enthalpies of formation of n-alkan-1-ols. *Journal of the Chemical Society, Faraday Transactions 1: Physical Chemistry in Condensed Phases* **1975**, *71* (0), 417. DOI: 10.1039/f19757100417.
(38) Holmes, J. L.; Aubry, C.; Mayer, P. M. Proton Affinities of Primary Alkanols: An Appraisal of the Kinetic Method. *The Journal of Physical Chemistry A* **1999**, *103* (6), 705-709. DOI: 10.1021/jp984094z.
(39) Chao, J.; Rossini, F. D. Heats of Combustion, Formation, and Isomerization of Nineteen Alkanols. *Journal of Chemical & Engineering Data* **1965**, *10* (4), 374-379. DOI: 10.1021/je60027a022.
(40) Edwards, J. O.; Pearson, R. G. The Factors Determining Nucleophilic Reactivities. *Journal of the American Chemical Society* **1962**, *84* (1), 16-24. DOI: 10.1021/ja00860a005.
(41) Jencks, W. P.; Carriuolo, J. Reactivity of nucleophilic reagents toward esters. *Journal of the American Chemical Society* **1960**, *82* (7), 1778-1786. DOI: 10.1021/ja01492a058.
(42) Potts, D. S.; Jeyaraj, V. S.; Kwon, O.; Ghosh, R.; Mironenko, A. V.; Flaherty, D. W. Effect of Interactions between Alkyl Chains and Solvent Structures on Lewis Acid Catalyzed Epoxidations. *ACS Catalysis* **2022**, *12* (21), 13372-13393. DOI: 10.1021/acscatal.2c03493.
(43) Gottlieb, H. E.; Kotlyar, V.; Nudelman, A. NMR chemical shifts of common laboratory solvents as trace impurities. *Journal of organic chemistry* **1997**, *62* (21), 7512-7515. DOI: 10.1021/jo971176v.
